# Temporal relationship among adiposity, gut microbiota, and insulin resistance in a longitudinal human cohort

**DOI:** 10.1186/s12916-022-02376-3

**Published:** 2022-05-19

**Authors:** Kui Deng, Menglei Shuai, Zheqing Zhang, Zengliang Jiang, Yuanqing Fu, Luqi Shen, Ju-Sheng Zheng, Yu-ming Chen

**Affiliations:** 1 Guangdong Provincial Key Laboratory of Food, Nutrition and Health, Department of Epidemiology, School of Public Health, Sun Yat-sen University, Guangzhou, China; 2 Institute of Basic Medical Sciences, Westlake Institute for Advanced Study, Hangzhou, China; 3 Key Laboratory of Growth Regulation and Translational Research of Zhejiang Province, School of Life Sciences, Westlake University, 18 Shilongshan Rd, Cloud Town, Hangzhou, China; 4 Department of Nutrition and Food Hygiene, Guangdong Provincial Key Laboratory of Tropical Disease Research, School of Public Health, Southern Medical University, Guangzhou, China; 5 Westlake Intelligent Biomarker Discovery Lab, Westlake Laboratory of Life Sciences and Biomedicine, Hangzhou, China

**Keywords:** Adiposity, Gut microbiota, Insulin resistance, Longitudinal cohort study, Obesity, Temporal relationship, Weight change

## Abstract

**Background:**

The temporal relationship between adiposity and gut microbiota was unexplored. Whether some gut microbes lie in the pathways from adiposity to insulin resistance is less clear. Our study aims to reveal the temporal relationship between adiposity and gut microbiota and investigate whether gut microbiota may mediate the association of adiposity with insulin resistance in a longitudinal human cohort study.

**Methods:**

We obtained repeated-measured gut shotgun metagenomic and anthropometric data from 426 Chinese participants over ~3 years of follow-up. Cross-lagged path analysis was used to examine the temporal relationship between BMI and gut microbial features. The associations between the gut microbes and insulin resistance-related phenotypes were examined using a linear mixed-effect model. We examined the mediation effect of gut microbes on the association between adiposity and insulin resistance-related phenotypes. Replication was performed in the HMP cohort.

**Results:**

Baseline BMI was prospectively associated with levels of ten gut microbial species. Among them, results of four species (*Adlercreutzia equolifaciens, Parabacteroides unclassified, Lachnospiraceae bacterium 3 1 57FAA CT1, Lachnospiraceae bacterium 7 1 58FAA*) were replicated in the independent HMP cohort. *Lachnospiraceae bacterium 3 1 57FAA CT1* was inversely associated with HOMA-IR and fasting insulin. *Lachnospiraceae bacterium 3 1 57FAA CT1* mediated the association of overweight/obesity with HOMA-IR (FDR<0.05). Furthermore, *Lachnospiraceae bacterium 3 1 57FAA CT1* was positively associated with the butyrate-producing pathway PWY-5022 (*p* < 0.001).

**Conclusions:**

Our study identified one potentially beneficial microbe *Lachnospiraceae bacterium 3 1 57FAA CT1*, which might mediate the effect of adiposity on insulin resistance. The identified microbes are helpful for the discovery of novel therapeutic targets, as to mitigate the impact of adiposity on insulin resistance.

**Supplementary Information:**

The online version contains supplementary material available at 10.1186/s12916-022-02376-3.

## Background

Adiposity/obesity has become a major public health issue, with a global prevalence of 13% in 2016 [1]. One of the major threats of adiposity is its detrimental impact on the insulin resistance [2]. Identification of novel intervention strategies, as to mitigate the strong link between adiposity and insulin resistance, is an emerging research topic.

Gut microbiota is closely involved in the etiology of adiposity and insulin resistance based on evidence from rodent models and human studies [3–5]. Yet, whether some gut microbial features lie in the pathways from adiposity to insulin resistance is less clear. More fundamentally, it has not been clarified in humans whether and how adiposity status affects gut microbiota. There have been some cross-sectional studies describing the correlations between adiposity indicators (BMI, weight, etc.) and gut microbial profiles, while few studies tried to investigate the direction of the association in humans [3, 6–8]. Demonstration of the longitudinal association of adiposity with the gut microbiota appears to be an essential step to understand whether gut microbes could be good intervention targets to alleviate the impact of adiposity on insulin resistance.

A major challenge in the field is to repeatedly collect adiposity and gut microbiota data over time, which would enable temporal analysis between the adiposity and gut microbiota. Although such datasets are available in several European and American cohorts [9–11], none of them reported the temporal bidirectional relationships among adiposity and gut microbiota. Moreover, given the high heterogeneity in the gut microbiota across different populations with diverse dietary cultures and ethnicities [12, 13], description of the above relationships in other ethnic groups is of important value.

Therefore, in the present study, we aimed to investigate the temporal bidirectional relationship between adiposity and gut microbiota (shotgun metagenome) over ~3 years, and to identify gut microbes that were affected by adiposity and long-term weight change among a Chinese middle-aged and elderly population. In addition, we aimed to assess whether gut microbiota could mediate the association between adiposity and insulin resistance-related phenotypes.

## Methods

### Study design and participants

**Fig. 1:**
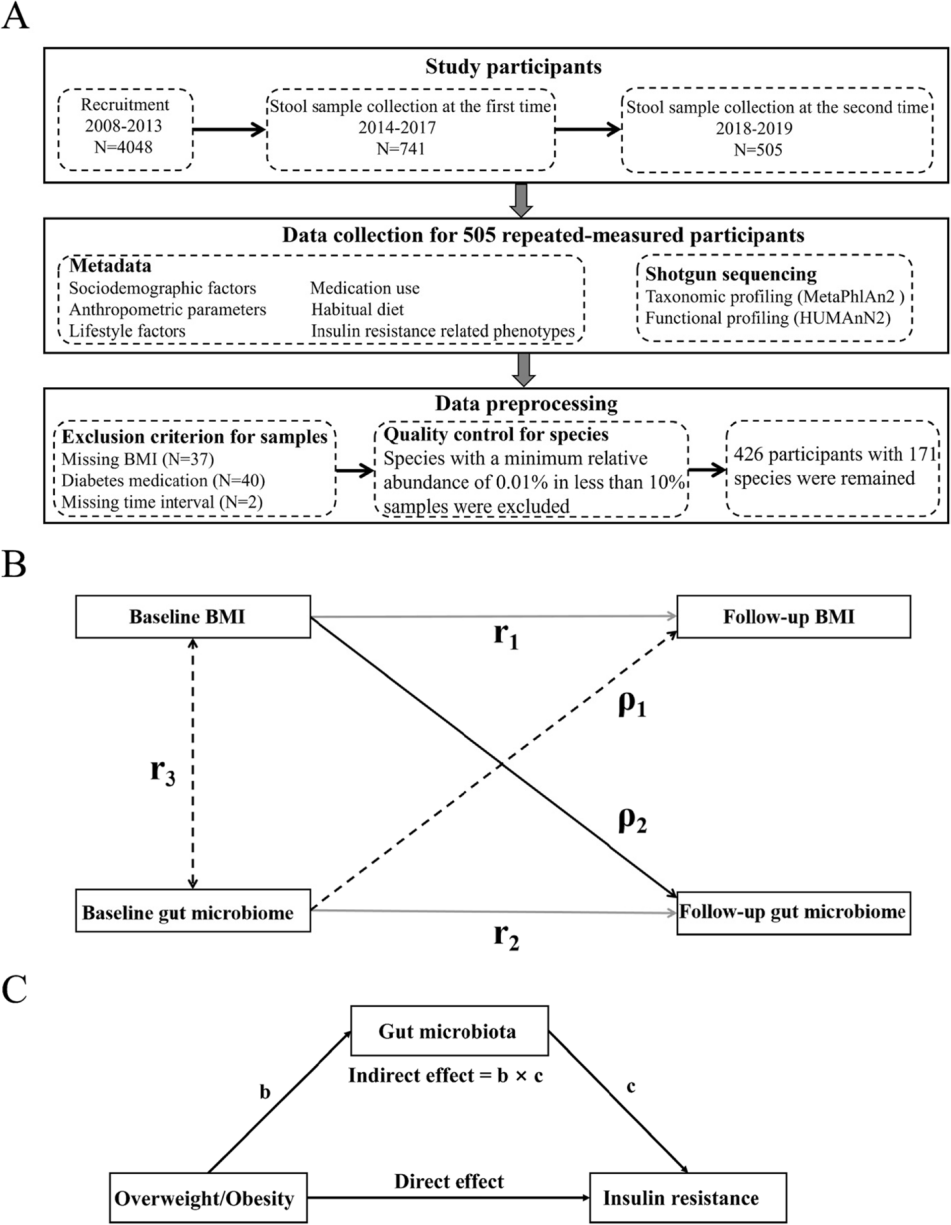
Study design and analytical method. **A** This study was based on the Guangzhou Nutrition and Health Study. A total of 741 stool samples were first collected between 2014 and 2017. Among them, 505 follow-up stool samples were repeatedly collected between 2018 and 2019. Metadata including sociodemographic factors, anthropometric parameters, lifestyle factors, medication use, habitual diet, and insulin resistance-related phenotypes were collected for the 505 participants. Shotgun metagenomic sequencing was performed for these 505 paired stool samples. After excluding participants who met the exclusion criterion and performing quality control for species, 426 participants with 171 species remained for subsequent analysis. **B** The cross-lagged panel analysis of BMI and gut microbiota. ρ1 and ρ2 are cross-lagged path coefficients; r1 and r2 are tracking correlations; r3 is the synchronous correlation between BMI and gut microbiota at baseline. **C** Gut microbiota mediates the association between overweight/obesity and insulin resistance

This study was based on the Guangzhou Nutrition and Health Study (GNHS), involving 4048 Chinese participants aged 40–75 years who lived in Guangzhou, China, for at least 5 years and were recruited between 2008 and 2013. Stool samples from 741 GNHS participants were collected between 2014 and 2017 (as baseline for the current study). Among them, stool samples were repeatedly collected in 505 participants between 2018 and 2019 (a follow-up collection). The median follow-up time was 3.15 years. We excluded participants with missing information on BMI at baseline or follow-up (*n* = 37), missing follow-up time (*n* = 2), or who had diabetes medications at baseline or follow-up (*n* = 40). Finally, 426 participants remained for subsequent analysis. The study design of this study is showed in Fig. 1A. This study was approved by the Ethics Committee of the School of Public Health at Sun Yat-sen University and Ethics Committee of Westlake University. All participants involved in this study provided written informed consent.

### Measurement of BMI, covariates, and insulin resistance-related phenotypes

Anthropometric parameters, including height, weight, and waist circumference (WC), were measured by trained staff on site, and BMI was calculated as weight (kg)/height (m)^2^. The information of sociodemographic, lifestyle covariates, and medication use was collected using a structured questionnaire during face-to-face interviews. Physical activity was assessed as total metabolic equivalent for task (MET) hours per day by a validated physical activity questionnaire which included 19 items [14]. Total energy intake was calculated based on the habitual dietary intakes over the past 12 months, which were collected using a validated food frequency questionnaire including 79 items [15]. Bristol stool score was immediately recorded in the stool sampler by the participants.

Fasting venous blood samples were drawn at both baseline and follow-up. Fasting insulin was measured by electrochemiluminescence immunoassay using a Roche Cobas 8000/e602 immunoanalyzer (Roche Diagnostics, Shanghai, China). Fasting glucose was measured through colorimetric methods using a Roche cobas 8000 c702 automated analyzer (Roche Diagnostics, Shanghai, China). Glycated hemoglobin A1c (HbA1c) was measured by high performance liquid chromatography using Bole D-10 Hemoglobin A1c Program on Bole D-10 Hemoglobin Testing System. Homeostasis model assessment of insulin resistance (HOMA-IR) was calculated based on fasting insulin and fasting glucose, which measures insulin resistance by using the mathematical model [16].

### Shotgun metagenomic sequencing, and taxonomic and functional profiling

The same protocol for stool sample collection and processing was used at baseline and follow-up visits. Fecal samples from all participants were collected on the examination day at the site, and then stored at – 80 °C within 4 h. Fecal DNA extraction and metagenomic sequencing followed the same protocol at the two time points. Microbial DNA was isolated using the QIAamp DNA Stool Mini Kit (Qiagen, Hilden, Germany) based on the manufacturer’s instruction. DNA concentrations were determined by the Qubit quantification system (Thermo Scientific, Delaware, USA). The shotgun metagenome was sequenced through the Illumina HiSeq platform (Illumina Inc., CA, USA) using the 2 × 300-bp paired-end read protocol. We obtained an average of 44.6 million (min, 22.1 million; max, 100 million) paired-end raw reads for each sample. The detailed information on bioinformatics analysis of the metagenome data could be found in our previous paper [17]. PRINSEQ (version 0.20.447) was employed to filter the reads with low-quality scores, with the following filtering parameters: (1) trim the reads by quality score from the 5′ end and 3′ end with a quality threshold of 20; (2) remove read pairs when either read was < 60 bp, contained “N” bases or quality score mean bellow 30; and (3) deduplicate the reads. Reads that could be aligned to the human genome (*H. sapiens*, UCSC hg19) were removed (aligned with Bowtie2 v2.2.5 using –reorder –no-contain –dovetail) [18]. Taxonomic profiling of the metagenomic samples was performed using MetaPhlAn2 (version 2.6.02) which used a library of clade-specific markers to provide pan-microbial (bacterial, archaeal, viral and eukaryotic) quantification at the species level [19]. MetaPhlAn2 was run using default settings. Microbial species with a minimum detective relative abundance of 0.01% in at least 10% of the samples were included, which yielded 171 species. Functional profiling of the metagenomic samples was performed using HUMAnN2 (version 2.8.1) using default settings [20], which generated microbial pathways based on MetaCyc metabolic pathway database [21, 22]. We applied log-transformation for the relative abundances of both species and pathway features before subsequent analysis. Before log-transformation, we replaced the zeros with a very small value (0.00001 for gut microbiota data and 1e−09 for functional pathway data), which may not substantially change the distribution of the data.

### Statistical analysis

The characteristics of the participants were presented as mean (SD) and median (quartile 1 [Q1], quartile 3 [Q3]) for continuous variables with normal and skewed distribution, respectively, and as frequency (percentage) for categorical variables. The differences of participant characteristics between baseline and follow-up were tested using paired *t* test and Wilcoxon signed rank test for continuous variables with normal and skewed distribution, respectively, and using the chi-squared test for categorical variables.

We used a cross-lagged panel design to investigate the bidirectional relationship between BMI and gut microbial features (Fig. 1B). Cross-lagged path analysis is a form of path analysis that examines reciprocal, longitudinal relationships among a set of inter-correlated variables [23–25]. This method tested the effect of baseline gut microbiota on subsequent BMI (ρ1 in Fig. 1B) and the effect of baseline BMI on subsequent gut microbiota (ρ2 in Fig. 1B) simultaneously, adjusted for autoregressive effects. Before performing the cross-lagged path analysis, we performed the linear regression analysis and got the residual of the baseline and follow-up values of BMI, adjusted for potential confounders, including age, sex, smoking status, alcohol status, education, income, physical activity, and total energy intake and then standardized the residual into *Z*-scores; gut microbial features were processed in the same manner with an additional adjustment for Bristol stool score. Pearson correlation coefficients of *Z*-transformed BMI and gut microbial features at baseline and follow-up were calculated, adjusted for the time interval (years) between two time points. The cross-lag path coefficients (ρ1 and ρ2) showed in Fig. 1B were estimated simultaneously based on the correlation matrix. All parameters in the cross-lagged path analysis were estimated through constructing structural equation model by R package lavaan (version 0.6–8) [26]. The validity of model fitting was evaluated by the standardized root mean square residual (SRMR) and comparative fit index (CFI) [27].

We performed a principal coordinates (PCo) analysis based on Bray-Curtis dissimilarity using metagenomic data at the species level, and the first two PCo (PCo1 and PCo2) were obtained to reflect the β-diversity of the gut microbiota. We calculated α-diversity indices (Observed species, Shannon index, Simpson index, Pielou’s evenness) based on the relative abundance of the species by using the R package vegan (version 2.5-7) [28]. We examined the temporal relationships between BMI and α-diversity, β-diversity, and individual microbes using the cross-lagged path analysis. The Benjamini-Hochberg (BH) method was used to control the false discovery rate (FDR). Given that high-dimensional tests were performed, associations with FDR < 0.25 were considered statistically significant for per-species test. The identified BMI-associated species were selected for subsequent analysis. The stratified analysis by sex for the association of BMI with the identified species was performed, and the heterogeneity in the effect sizes between females and males were tested using the Cochran-Q test [29]. We further did sensitivity analysis to investigate the temporal relationship between WC and gut microbiota. We assessed the prospective associations between dietary factors (vegetable intake, fruit intake, fish intake, red and processed meat intake, and dairy intake) and identified gut microbes using the multivariable linear regression models, adjusted for age, sex, BMI, smoking status, alcohol status, education, income, physical activity, total energy intake, Bristol stool score, time interval, and corresponding baseline microbe abundance. Each dietary factor was divided into higher and lower groups based on the median value.

To replicate the results from the above GNHS participants, we used the repeated-measured fecal metagenome data available 1 year apart from 43 healthy participants aged 18–40 years in the HMP cohort [9, 11]. We obtained the relative abundance of microbiota data through the R package curatedMetagenomicData (version 1.10.2) [30], and the corresponding phenotype data from the dbGaP (https://dbgap.ncbi.nlm.nih.gov/; study accession: phs000228.v4.p1) [31, 32]. The relative abundances of the species were log-transformed before formal analysis. Since BMI was only available at baseline, we assessed the prospective association of baseline BMI with follow-up microbes using multivariable linear regression models, adjusted for age, sex, race (white/not white), time interval, and corresponding baseline microbe abundance. We used the meta-analysis with a random effects model and inverse-variance weights to integrate the results from GNHS and HMP cohorts, and assessed the heterogeneity between them using *I*^2^ and Cochran-Q test [29]. We considered the association between baseline BMI and follow-up microbes to be replicable if *p*_meta_<0.05, *p*_heterogeneity_>0.05, *I*^2^<50%, and the same direction of associations between the two cohorts. The meta-analysis and Cochran-Q test were conducted using the R package metafor (version 3.0-2) [33].

In the GNHS, to explore the impact of long-term weight change on gut microbiota, participants were divided into four weight change patterns: stable normal (*n* = 205), normal to adiposity (*n* = 23), adiposity to normal (*n* = 21), and stable adiposity (*n* = 156). Participants who were underweight at either time point were excluded (*n* = 21). Adiposity was defined as overweight or obesity in this study. According to the suggestion of Working Group On Obesity In China for Chinese populations [34], underweight, normal weight, overweight, and obesity were defined as BMI<18.5, 18.5≤BMI≤23.9, 24≤BMI≤27.9, and BMI≥28, respectively. The influence of weight change pattern on follow-up microbial features (α-diversity: Observed species, Shannon index, Simpson index, Pielou’s evenness; β-diversity: PCo1 and PCo2; and microbes) was assessed using multivariable linear regression models, adjusted for age, sex, smoking status, alcohol status, education, income, physical activity, total energy intake, Bristol stool score, time interval, and corresponding baseline microbial features. We only included BMI-associated microbes in the analysis of the association between weight change pattern and gut microbes. The stable normal group was served as the reference group when conducting the above analyses. The potential collinearity among covariates was assessed by variance inflation factor (VIF) using R function vif in R package car (version 3.0-12), and VIF > 10 indicates collinearity among variables. After fitting 16 multivariable linear regression models for microbial features (10 identified BMI-associated microbes plus Observed species, Shannon index, Simpson index, Pielou’s evenness, PCo1 and PCo2), we obtained 16 VIF for each covariate and the ranges of them were as follows: age (1.175–1.187), sex (1.486–1.525), smoking status (1.336–1.343), alcohol status (1.167–1.180), education (1.306–1.321), income (1.384–1.408), physical activity (1.068–1.074), total energy intake (1.130–1.137), Bristol stool score (1.086–1.124), time interval (1.064–1.077), corresponding baseline microbial features (1.029–1.102), which confirmed that there was no collinearity among covariates.

We explored the associations between the above identified species and HOMA-IR using linear mixed-effect models by R package lme4 (version 1.1-27.1) [35], adjusted for age, sex, smoking status, alcohol status, education, income, physical activity, and total energy intake. In the secondary analyses, we analyzed the association of identified species with fasting insulin, fasting glucose, and HbA1c using linear mixed-effect models, adjusted for the same covariates. We log-transformed insulin resistance-related phenotypes with skewed distribution (HOMA-IR, fasting insulin, and fasting glucose) before analysis. All phenotypes and microbes were then standardized into *Z*-scores. We also assessed whether dietary factors (vegetable intake, fruit intake, fish intake, red and processed meat intake, and dairy intake) contributed to the insulin resistance-related phenotypes by using multivariable linear regression models, adjusted for age, sex, BMI, smoking status, alcohol status, education, income, physical activity, total energy intake, time interval, and corresponding baseline levels of insulin resistance-related phenotypes. Each dietary factor was divided into higher and lower groups based on the median value.

To investigate whether there were potential mediation effects of gut microbes on the association between adiposity and insulin resistance, we performed a mediation analysis using the R package mediation (version 4.5.0) [36]. The baseline overweight/obesity status was served as the exposure, and follow-up gut microbes and insulin resistance-related phenotypes were served as mediators and outcomes, respectively (Fig. 1C). The corresponding baseline gut microbes and insulin resistance-related phenotypes were adjusted in the statistical models. The covariates included age, sex, smoking status, alcohol status, education, income, physical activity, total energy intake, Bristol stool score, and time interval. Before performing mediation analysis, we examined the prospective associations between weight group (underweight, normal weight, and adiposity) and insulin resistance-related phenotypes (HOMA-IR, fasting insulin, fasting glucose, and HbA1c) using multivariable linear regression models, adjusted for age, sex, smoking status, alcohol status, education, income, physical activity, total energy intake, time interval, and corresponding baseline levels of insulin resistance-related phenotypes.

One identified species *Lachnospiraceae bacterium 3 1 57FAA CT1* belonged to Lachnospiraceae which is involved in the production of short-chain fatty acid (SCFA) through fermenting plant polysaccharides [37, 38]. In addition, *Lachnospiraceae bacterium 3 1 57FAA CT1* has phylogenetic similarity to known butyrogenic gut bacteria [39]. The butyrate-producing pathway PWY-5022 (4-aminobutanoate degradation V) was extracted from the pathway data obtained from functional profiling of the metagenomic samples. We used a linear mixed-effect model to examine the association between *Lachnospiraceae bacterium 3 1 57FAA CT1* and PWY-5022, adjusted for age, sex, smoking status, alcohol status, education, income, physical activity, total energy intake, and Bristol stool score. Additionally, the associations between *Lachnospiraceae bacterium 3 1 57FAA CT1* and other pathways in addition to PWY-5022 were also assessed using linear mixed-effect models, adjusted for the same covariates described above. We only included microbial pathways with a minimum detective relative abundance of 0.01% in at least 10% of the samples for this analysis. Unless otherwise noted, FDR<0.05 was considered statistically significant in this study.

## Results

### Characteristics of study participants

The mean (SD) age of these participants was 63.6 (5.4) years (31.9% men) when their first batch of stool samples were collected at baseline (Table 1). BMI was slightly increased from baseline to follow-up (*p* = 0.002), while WC was not statistically different between two time points (Additional file 1: Table S1). The prevalence of adiposity (overweight or obesity) for the baseline and follow-up is 41.5% and 42%, respectively. Insulin resistance-related phenotypes, including HOMA-IR, fasting insulin, HbA1c, and fasting glucose, were increased from baseline to follow-up (*p*<0.05; Additional file 1: Table S1).

**Table 1:** Characteristics of the study participants at the baseline^a^

Characteristics	Female (***n*** = 290)	Male (***n*** = 136)
Age, years, mean (SD)	62.7 (4.4)	65.7 (6.7)
BMI, kg/m^2^, mean (SD)	23.1 (3.0)	24.2 (3.3)
Weight group, *n* (%)		
Lean	9 (3.1)	5 (3.7)
Normal	172 (59.3)	55 (40.4)
Overweight	92 (31.7)	60 (44.1)
Obesity	17 (5.9)	16 (11.8)
Adiposity (overweight or obesity), *n* (%)	102 (35.2)	75 (55.1)
Weight change pattern, *n* (%)		
Stable normal	154 (56)	51 (39.2)
Normal to adiposity	19 (6.9)	4 (3.1)
Adiposity to normal	12 (4.4)	9 (6.9)
Stable adiposity	90 (32.7)	66 (50.8)
Waist circumference, cm, mean (SD)	84.4 (8.6)	87.1 (9.0)
Current smoker, *n* (%)	0 (0)	31 (22.8)
Current alcohol drinker, *n* (%)	8 (2.8)	27 (19.9)
Education, *n* (%)		
Middle school or lower	70 (24.1)	30 (22.1)
High school or professional college	152 (52.4)	52 (38.2)
University	68 (23.4)	54 (39.7)
Income level, *n* (%)		
Extremely low (≤500 ¥/month)	2 (0.7)	3 (2.2)
Low (501–1500 ¥/month)	66 (22.8)	24 (17.6)
Middle (1501–3000 ¥/month)	179 (61.7)	94 (69.1)
High (>3000 ¥/month)	43 (14.8)	15 (11.0)
Fasting insulin, μU/mL, median (Q1, Q3)	6.6 (4.6, 9.6)	6.7 (4.2, 9.6)
HOMA-IR, median (Q1, Q3)	1.5 (1.0, 2.2)	1.5 (1.0, 2.2)
HbA1c, %, mean (SD)	5.6 (0.4)	5.7 (0.5)
Fasting glucose, mmol/L, median (Q1, Q3)	5.1 (4.8, 5.5)	5.1 (4.7, 5.5)
Physical activity, MET, mean (SD)	41.5 (14.1)	39.2 (12.6)
Total energy intake, kcal/day, mean (SD)	1709.7 (454.2)	1868.9 (454.2)
Vegetable intake, g/day, median (Q1, Q3)	366.9 (264.2, 492.4)	328.3 (246.9, 478.4)
Fruit intake, g/day, median (Q1, Q3)	144.0 (85.1, 218.4)	103.6 (57.4, 167.5)
Fish intake, g/day, median (Q1, Q3)	42.9 (23.9, 68.0)	39.0 (23.8, 64.6)
Red and processed meat intake, g/day, median (Q1, Q3)	76.6 (50.9, 107.7)	76.9 (53.0, 108.9)
Dairy intake, g/day, median (Q1, Q3)	16.61 (7.20, 27.51)	9.87 (2.25, 21.08)
Years of follow-up	3.2 (0.3)	3.1 (0.3)

^a^Data are expressed as mean (SD) and median (Q1, Q3) for continuous variables with normal and skewed distribution, respectively, and as frequency (percentage) for categorical variables

*SD* standard deviation, *Q1* quartile 1, *Q3* quartile3, *HOMA-IR* homeostasis model assessment of insulin resistance, *HbA1c* hemoglobin A1c, *MET* metabolic equivalent for task

### The temporal relationship between BMI and gut microbiota

**Fig. 2:**
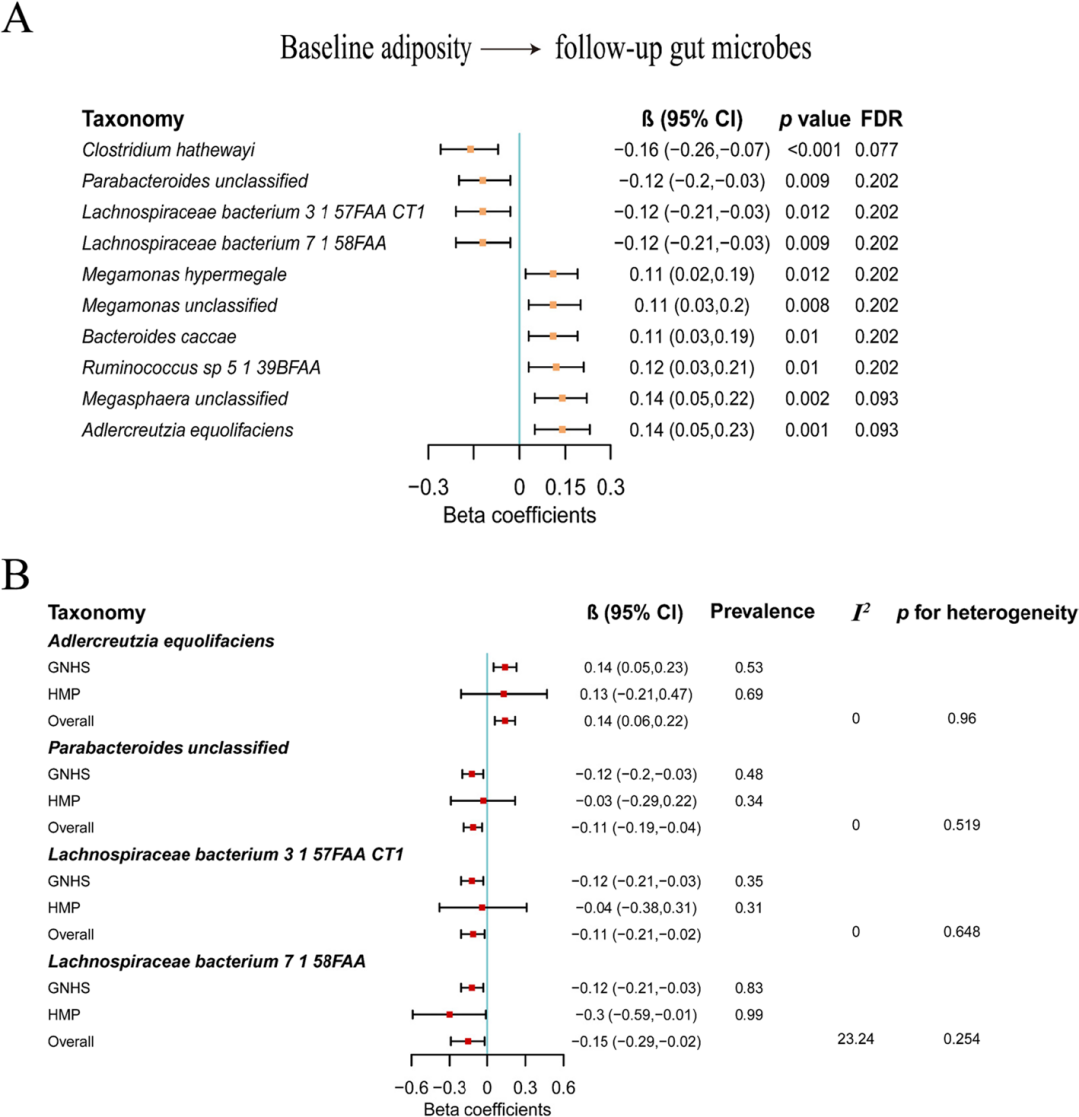
The prospective association between BMI and gut microbiota. **A** The associations between baseline adiposity and follow-up gut microbes. The cross-lagged path analysis was used to estimate the difference in the abundance of gut microbes (in SD unit of the log-transformed abundance) per 1-SD difference of BMI, adjusted for age, sex, smoking status, alcohol status, education, income, physical activity, total energy intake, Bristol stool score, and time interval. **B** Replication of the prospective associations of BMI with microbes in the HMP cohort. Multivariable linear regression models were used to estimate the difference in the abundance of gut microbes (in SD unit of the log-transformed abundance) per 1-SD difference in BMI, adjusted for age, sex, race (white/not white), time interval, and corresponding baseline microbe abundance. The meta-analysis with a random effects model was used to integrate the results from GNHS and HMP cohorts, and the heterogeneity was assessed using *I*^2^ and Cochran-Q test

The associations between the BMI and gut microbial α-diversity and β-diversity were not statistically significant in either direction (Additional file 1: Fig. S1A, Fig. S2A and Table S2). For the temporal relationship between BMI and individual gut microbes, we did not find any statistically significant associations for the path coefficients (ρ_1_) from baseline gut microbes to follow-up BMI (Additional file 1: Fig. S1B and Table S3). For the path coefficients (ρ_2_) from baseline BMI to follow-up gut microbes, ten species were identified (FDR<0.25; Fig. 2A and Additional file 1: Table S3). Among them, with the increase of BMI, the abundances of four species (*Clostridium hathewayi*, *Parabacteroides unclassified*, *Lachnospiraceae bacterium 3 1 57FAA CT1*, *Lachnospiraceae bacterium 7 1 58FAA*) were decreased, and the abundances of the other species (*Megamonas hypermegale*, *Megamonas unclassified*, *Bacteroides caccae*, *Ruminococcus sp 5 1 39BFAA*, *Megasphaera unclassified*, *Adlercreutzia equolifaciens*) were increased. *Adlercreutzia equolifaciens* belonged to Actinobacteria, *Parabacteroides unclassified* and *Bacteroides caccae* belonged to Bacteroidetes, and other species belonged to Firmicutes. When we used a more stringent cut-off value of 0.2 for FDR values, all identified ten microbes based on FDR<0.25 were also statistically significant (all FDR values less than 0.2 or very close to 0.2; Fig. 2A). All ten species had relatively high prevalence in our cohort (0.28–0.83; Additional file 1: Table S9). There were low to moderate correlations among these species (Additional file 1: Fig. S3). In addition, there were moderate correlations (from 0.12 to 0.46) of identified species between two time points (Additional file 1: Table S4). The stratified analysis by sex for the association of BMI with identified species suggested that there was no heterogeneity of the regression coefficients between females and males (FDR_heterogeneity_>0.05; Additional file 1: Table S5) and there was a strong correlation between the regression coefficients for females and those for males (*r* = 0.815; Additional file 1: Fig. S4). The association of WC with gut microbes were similar compared with those of BMI with gut microbes, with correlation coefficients 0.88 and 0.99 for all gut microbial features and the identified microbes, respectively (Additional file 1: Fig. S5, Table S6 and S7). For the contributions of dietary factors (vegetable intake, fruit intake, fish intake, red and processed meat intake, and dairy intake) to the identified microbes, we only found that *Ruminococcus sp 5 1 39BFAA* was associated with fish intake (FDR<0.05; Additional file 1: Table S8), while other microbes were not correlated with dietary factors (FDR>0.05; Additional file 1: Table S8).

In the Human Microbiome Project (HMP) cohort, the prevalence of *Megamonas hypermegale*, *Megamonas unclassified*, and *Megasphaera unclassified* was very low (0.05, 0.06, and 0.09, respectively; Additional file 1: Table S9). Results of the prospective associations between baseline BMI and four species (*Adlercreutzia equolifaciens, Parabacteroides unclassified, Lachnospiraceae bacterium 3 1 57FAA CT1, Lachnospiraceae bacterium 7 1 58FAA*) were replicated in the HMP cohort, with a *p*-value for meta-analysis across the two studies < 0.05, *p*_*heterogeneity*_>0.05, and *I*^2^<50% (Fig. 2B; Additional file 1: Table S9).

### Prospective association of long-term weight change pattern with gut microbiota

**Fig. 3:**
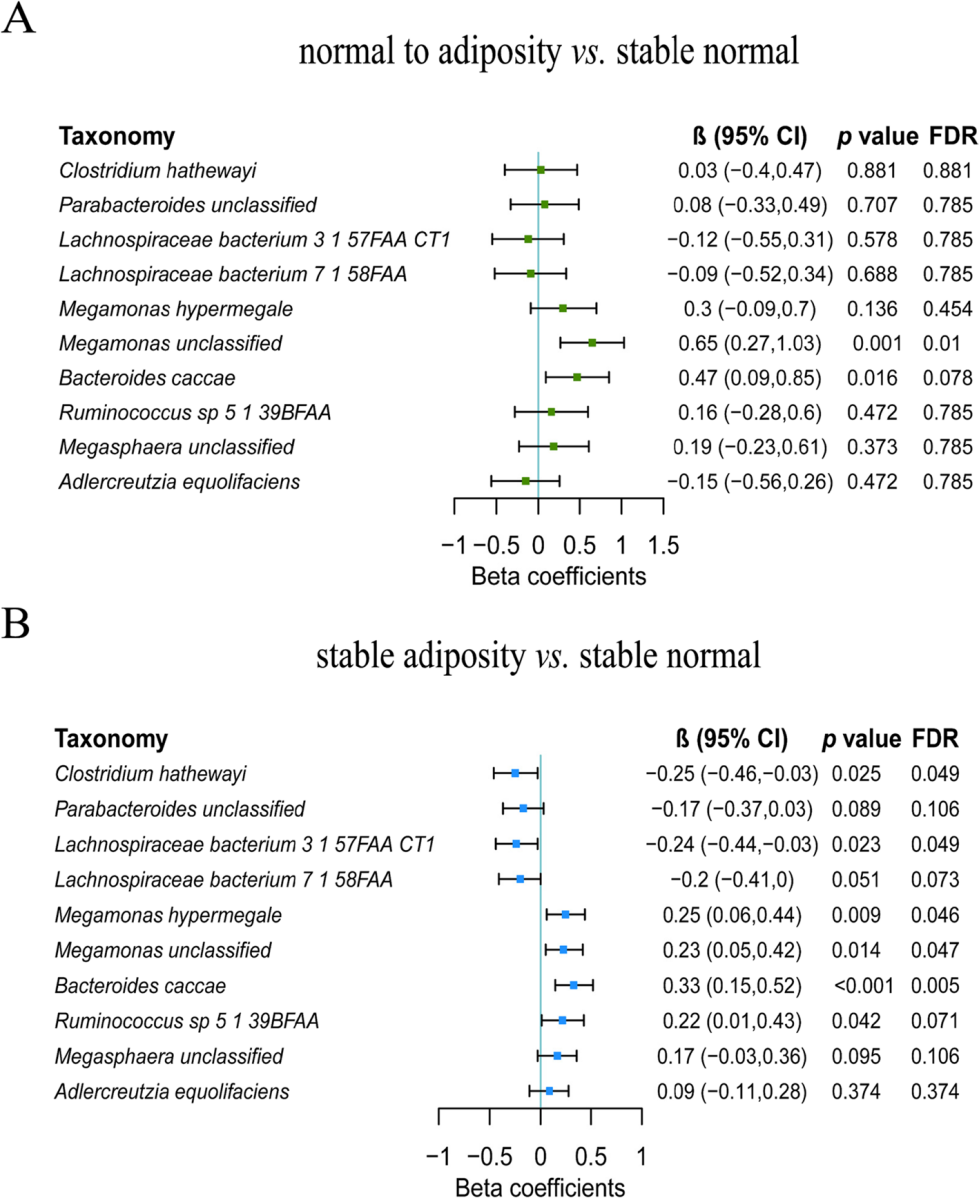
The prospective association between long-term weight change and gut microbiota. **A** Gut microbes that were associated with the normal to adiposity group compared with the stable normal group. Multivariable linear regression models were used to estimate the difference in the abundance of gut microbes (in SD unit of the log-transformed abundance) comparing the normal to adiposity group with the stable normal group, adjusted for age, sex, smoking status, alcohol status, education, income, physical activity, total energy intake, Bristol stool score, time interval, and corresponding baseline microbe abundance. **B** Gut microbes that were associated with the stable adiposity group compared with the stable normal group. Multivariable linear regression models were used to estimate the difference in the abundance of gut microbes (in SD unit of the log-transformed abundance) comparing the stable adiposity group with the stable normal group, adjusted for age, sex, smoking status, alcohol status, education, income, physical activity, total energy intake, Bristol stool score, time interval, and corresponding baseline microbe abundance. CI, confidence interval; FDR, false discovery rate

The prospective association of long-term weight change pattern with gut microbial β-diversity was statistically significant with reduced PCo2 comparing the normal to adiposity group with the stable normal group (Additional file 1: Fig. S1C). The long-term weight change pattern was not associated with any of the α-diversity indices (Additional file 1: Fig. S1C and Fig. S2B). Among microbes associated with the BMI, *Megamonas unclassified* was significantly higher in the normal to adiposity group compared with the stable normal group (FDR=0.01; Fig. 3A). Compared with the stable normal group, the abundances of *Clostridium hathewayi* and *Lachnospiraceae bacterium 3 1 57FAA CT1* were significantly lower, and the abundances of *Megamonas hypermegale*, *Megamonas unclassified*, and *Bacteroides caccae* were higher in the stable adiposity group (FDR<0.05; Fig. 3B). The mean abundance of identified microbes was 0.13%, 0.03%, 0.24%, 2.04%, and 0.92% for *Clostridium hathewayi*, *Lachnospiraceae bacterium 3 1 57FAA CT1*, *Megamonas hypermegale*, *Megamonas unclassified*, and *Bacteroides caccae*, respectively.

### The association between gut microbes and insulin resistance-related phenotypes

**Fig. 4:**
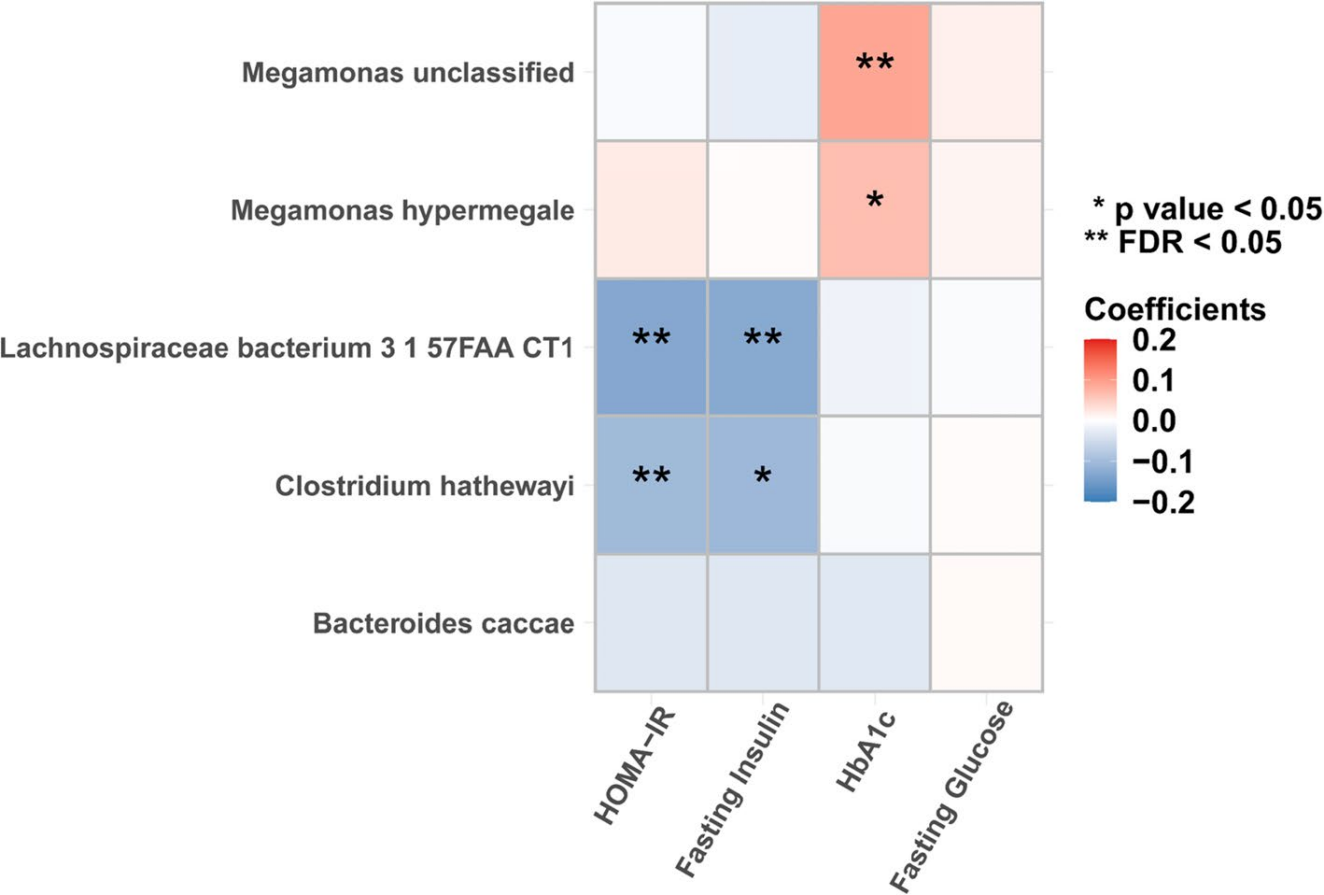
The associations between the identified microbes and insulin resistance-related phenotypes. Linear mixed-effect models were used to estimate the difference in insulin resistance-related phenotypes (in SD unit) per 1-SD difference in the log-transformed abundance of gut microbes, adjusted for age, sex, smoking status, alcohol status, education, income, physical activity, and total energy intake. Fasting insulin, HOMA-IR, and fasting glucose were log-transformed. HOMA-IR, homeostasis model assessment of insulin resistance; HbA1c, hemoglobin A1c; FDR, false discovery rate

*Lachnospiraceae bacterium 3 1 57FAA CT1* [*β* = − 0.13, 95% CI (− 0.22, − 0.05), FDR = 0.014] and *Clostridium hathewayi* [*β* = − 0.1, 95% CI (− 0.18, − 0.02), FDR = 0.03] were negatively associated with HOMA-IR (Fig. 4 and Additional file 1: Table S10). In the secondary analyses, *Lachnospiraceae bacterium 3 1 57FAA CT1* was negatively associated with fasting insulin [*β* = − 0.13, 95% CI (− 0.22, − 0.04), FDR = 0.037], and *Megamonas unclassified* had a positive association with HbA1c [*β* = 0.09, 95% CI (0.03, 0.15), FDR = 0.037] (Fig. 4 and Additional file 1: Table S10). For the contributions of dietary factors (vegetable intake, fruit intake, fish intake, red and processed meat intake, and dairy intake) to insulin resistance, we did not find any dietary factor that was prospectively associated with insulin resistance-related phenotypes (FDR > 0.05; Additional file 1: Table S11).

### The mediation effects of the gut microbes on the association between adiposity and insulin resistance-related phenotypes

**Fig. 5:**
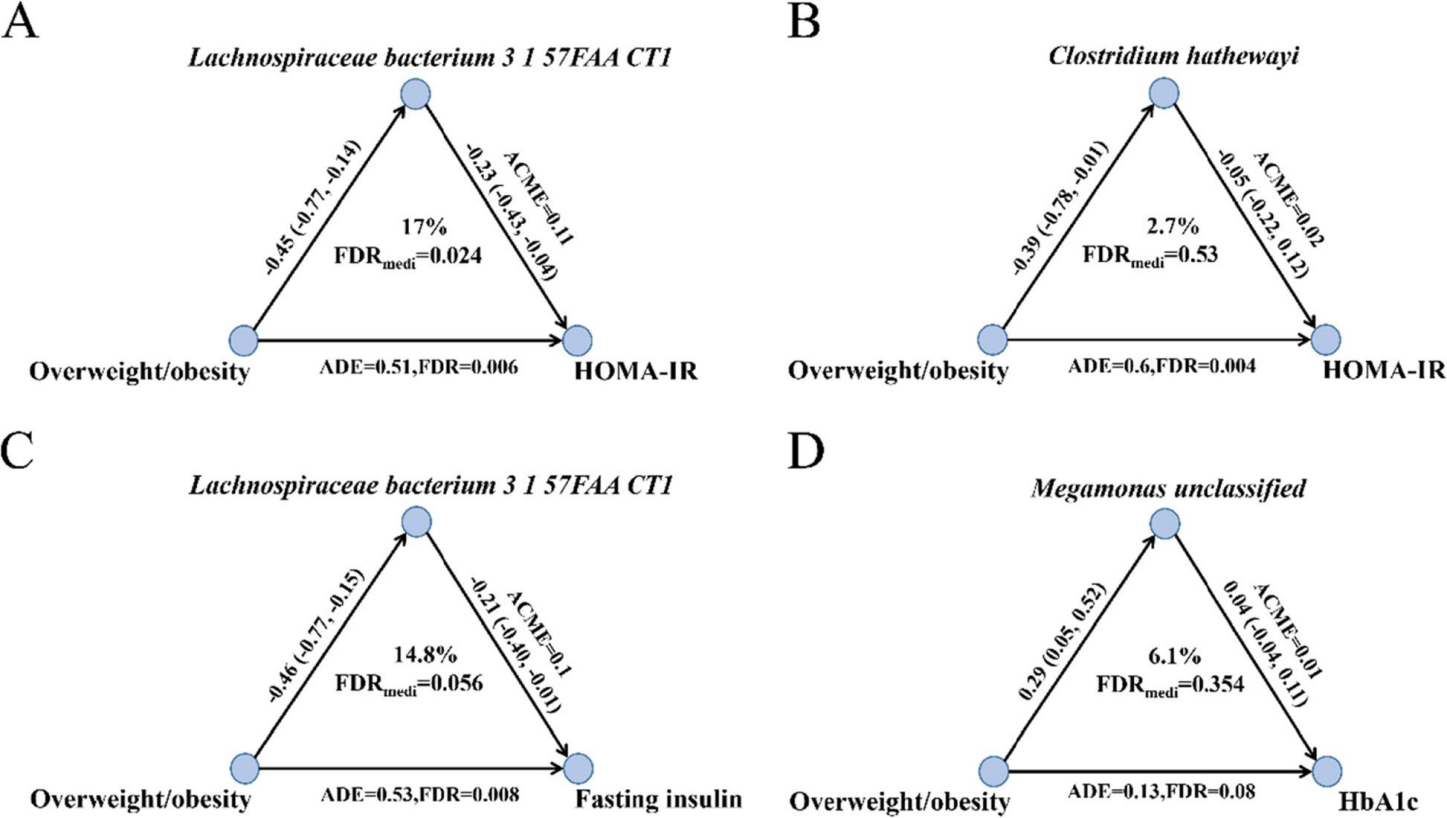
Mediation analysis for the role of gut microbes in the association between adiposity and insulin resistance-related phenotypes. **A** The mediation effect of *Lachnospiraceae bacterium 3 1 57FAA CT1* on the association between adiposity and HOMA-IR. **B** The mediation effect of *Clostridium hathewayi* on the association between adiposity and HOMA-IR. **C** The mediation effect of *Lachnospiraceae bacterium 3 1 57FAA CT1* on the association between adiposity and fasting insulin. **D** The mediation effect of *Megamonas unclassified* on the association between adiposity and HbA1c. In the mediation analysis, age, sex, smoking status, alcohol status, education, income, physical activity, total energy intake, Bristol stool score, time interval, and corresponding baseline gut microbes and insulin resistance-related phenotypes were adjusted. ACME, average causal mediation effects; ADE, average direct effect; FDR, false discovery rate; HOMA-IR, homeostasis model assessment of insulin resistance; HbA1c, hemoglobin A1c

We first examined the associations between weight group (underweight, normal weight, and adiposity) and insulin resistance. We found that adiposity was positively associated with several insulin resistance-related phenotypes including HOMA-IR, fasting insulin, and HbA1c compared with the normal weight (FDR<0.05; Additional file 1: Table S12). The results of mediation analysis showed that the *Lachnospiraceae bacterium 3 1 57FAA CT1* mediated the association of adiposity with HOMA-IR (17%, FDR = 0.024; Fig. 5A). *Clostridium hathewayi* did not have the mediation role in the association between adiposity and HOMA-IR (2.7%, FDR = 0.53; Fig. 5B). In the secondary analyses, the mediation effect of *Lachnospiraceae bacterium 3 1 57FAA CT1* on the association between adiposity and fasting insulin was borderline significant (14.8%, FDR = 0.056; Fig. 5C). The mediation role of *Megamonas unclassified* on the association of adiposity with HbA1c was not statistically significant (6.1%, FDR = 0.354; Fig. 5D). As all average causal mediation effects (ACME) and average direct effect (ADE) were larger than zero, the mediation effects and direct effects in the overweight/obesity group were larger than those in the normal group for all above mediation analyses (Fig. 5). We further found that *Lachnospiraceae bacterium 3 1 57FAA CT1* was associated with a higher level of the butyrate-producing pathway PWY-5022 [*β* = 0.12, *p* < 0.001; Additional file 1: Table S13). In addition, *Lachnospiraceae bacterium 3 1 57FAA CT1* was also associated with several other microbial functional pathways, such as amino acid biosynthesis and degradation pathways (HISDEG-PWY, PWY-4981, PWY-5101, DAPLYSINESYN-PWY, PWY-5030, PWY-2942, PWY-5154, HSERMETANA-PWY, PWY-6630, PWY-5097, PWY-6629), anaerobic energy metabolism (PWY-7383), lipid metabolism (PWY-5667, PWY0-1319), and gluconeogenesis (PWY66-399) (FDR < 0.05; Additional file 1: Table S14).

## Discussion

In the present longitudinal cohort study with repeated-measured gut microbiota and adiposity data, we identified ten microbes that were prospectively associated with the baseline BMI, and no baseline microbes were found to be associated with future BMI. Among them, associations of BMI with four microbes were replicated in the HMP cohort. In addition, there were significant associations between the identified gut microbes and several insulin resistance-related phenotypes (HOMA-IR, fasting insulin, and HbA1c). We demonstrated that *Lachnospiraceae bacterium 3 1 57FAA CT1* may mediate the association of adiposity with insulin resistance.

The associations between adiposity and human gut microbiota were explored in several cross-sectional studies over the past few years [3, 6, 7]. Most of these studies focused on identifying gut microbes that could affect the development of adiposity [40]. Although the causal role of gut microbiota in adiposity development has been established in mice [4], how and what gut microbes may be longitudinally associated with adiposity status in humans was not clear. On the other hand, adiposity has huge impacts on the diversity of gut microbiota, as shown in mice [41]. However, few studies explored the association of adiposity with gut microbiota in human longitudinal cohorts, which might be helpful for the discovery of new therapeutic targets for adiposity-associated diseases. The temporal relationship between adiposity and gut microbiota was unexplored, due to the scarcity of dynamic microbiota data in large human studies. The direction of their associations could be resolved using repeated collected fecal microbiota data overtime, such datasets are currently available among several Western and American populations [9–11]. Our study, to the best of our knowledge, presented the first repeated-measured gut metagenome data in Chinese populations, which could be helpful for revealing the dynamic change of gut microbiota overtime. In addition, prior cross-sectional studies explored the association between BMI and gut microbiota, mostly using data from 16S rRNA sequencing [6, 7]. In the present study, metagenomic data were used to detect gut microbes at species level, which improved the taxonomic precision.

In our assessment of the temporal relationship between BMI and gut microbiota, we did not find any baseline gut microbes that were associated with future BMI levels. Instead, we identified several gut microbes longitudinally associated with baseline BMI in the present study. The inverse association between BMI and *Parabacteroides* was also observed in a cross-sectional study among Chinese college students using 16S rRNA sequencing [6]. Results from another cross-sectional study showed lower levels of *Parabacteroides* and higher levels of *Megasphaera* in overweight/obesity patients compared with normal weight controls among Italian adults using 16S rRNA sequencing [7]. The association between BMI and *Adlercreutzia equolifaciens* was also identified in a study using metagenome data from the northern Netherland [9]. However, the directions of the effects could not be clarified in the above prior studies. Furthermore, we replicated the results of the prospective associations of baseline BMI with four follow-up microbes in the HMP cohort that was mainly composed of Caucasians in the US and had completely different age coverage with our GNHS cohort. The identified associations that could not validated in the HMP cohort may be specific for Chinese populations. However, given that the sample size of the HMP cohort was small (only 43 participant), it should be further validated whether the identified associations in our cohort are specific for Chinese populations in the future larger studies.

Previous studies demonstrated that stable adiposity and weight gain were associated with increased risk of cardiovascular disease and mortality [42, 43]. In this study, we found that long-term stable adiposity status was significantly associated with the abundance of gut microbes. We discovered that the abundance of *Megamonas unclassified* was higher in the normal to adiposity group compared with the stable normal group. Our study showed that the abundance of *Megamonas unclassified* was associated with higher levels of HbA1c. As adiposity is a risk factor for diabetes, *Megamonas unclassified* may contribute to the development of diabetes accompanying the development of overweight/obesity. The metabolic function of *Megamonas unclassified* was unexplored in the literature. Ling et al. reported that *Megamonas* was significantly correlated with systemic inflammatory cytokines (e.g., IL-6) [44]. As the inflammation has an important role in diabetes [45], *Megamonas unclassified* may be involved in the development of diabetes through the inflammation pathways. Intervention study conducted by Pisanu et al. demonstrated that a moderately hypocaloric Mediterranean diet could decrease the relative abundance of *Megamonas* for obese and overweight patients [46]. We noted that *Megamonas hypermegale*, *Megamonas unclassified*, and *Megasphaera unclassified* had higher prevalence in our cohort compared with the HMP cohort (36% *vs.* 5%, 52% *vs.* 6%, and 38% *vs.* 9%, respectively). As gut microbiome is mainly determined by genetics and environment, and there were diverse dietary cultures and ethnicities between Chinese (GNHS cohort) and Western populations (HMP cohort), the higher prevalence of these species in the GNHS cohort compared with the HMP cohort may be caused by the combined influence of genetics and diet. For example, we found that there was a positive association between fish intake and *Megamonas hypermegale* (*β* = 0.21, *p* = 0.021; Additional file 1: Table S8).

We identified a potentially beneficial microbe named *Lachnospiraceae bacterium 3 1 57FAA CT1*, which was associated with lower levels of HOMA-IR and fasting insulin. Long-term adiposity status was associated with a lower abundance of *Lachnospiraceae bacterium 3 1 57FAA CT1*. The mediation analysis further suggested that *Lachnospiraceae bacterium 3 1 57FAA CT1* mediated the association of adiposity with insulin resistance. In other words, adiposity may potentially induce insulin resistance through reducing the abundance of *Lachnospiraceae bacterium 3 1 57FAA CT1.* Therefore, *Lachnospiraceae bacterium 3 1 57FAA CT1* may serve as a potential intervention target for overweight/obese individuals to prevent and treat obesity-related insulin resistance. Many species in *Lachnospiraceae* were involved in the production of SCFA [37], and *Lachnospiraceae bacterium 3 1 57FAA CT1* had phylogenetic similarity to known butyrogenic gut bacteria [39]. In the present study, we speculated that *Lachnospiraceae bacterium 3 1 57FAA CT1* may be involved in the gut production of the butyrate through examining the association of this bacterium with butyrate-producing pathway PWY-5022. A prior study conducted by Sanna et al. showed that PWY-5022 was causally associated with improved insulin response following an oral glucose test and the reverse relationship was not significant [47]. In addition, Gao et al. showed that supplementation of butyrate could decrease the fasting insulin level in mice fed a high-fat diet [48]. Therefore, we hypothesize that *Lachnospiraceae bacterium 3 1 57FAA CT1* may drive the fasting insulin level and the potential beneficial association of *Lachnospiraceae bacterium 3 1 57FAA CT1* with insulin sensitivity may be explained by its potential to produce butyrate in the gut.

For the contributions of dietary factors to gut microbiota and insulin resistance-related phenotypes, most associations (except the association between fish intake and *Megamonas hypermegale*) were not significant in our samples. These null results do not mean that dietary factors are not important for the gut microbiome or insulin resistance, but rather due to the very limited sample size we used in this study. Usually for the study of FFQ-based dietary factors with microbiome or diseases, we need thousands of participants to increase the statistical power, as we did previously [49].

Our study has some strengths. First, it is based on a longitudinal cohort study with a median follow-up time of 3.15 years. Second, we, for the first time, apply the cross-lagged panel analysis with structural equation model to microbiota data, which could help reveal the temporal relationship between adiposity and gut microbiota. We admit that our study has some limitations. First, although we have adjusted several important confounding factors, we are unable to fully rule out the influence of residual confounding on our results due to the observational nature of our study. Second, our study only included one small replication cohort (HMP) as the repeated-measured fecal metagenome data was rare. There are only 43 participants in the cohort, which limits the power to fully replicate the present results. Larger replication cohorts are needed to make our findings more solid. Meanwhile, as the information of insulin resistance-related phenotypes is not provided in the HMP cohort, other findings in our study could not be validated. Therefore, the results in the present study should be further validated in other large repeated-measured cohorts or experimental studies in the future. Third, several weight change pattern groups have relatively small sample size (normal to adiposity group 23; adiposity to normal group 21), which limits the statistical power to detect significant associations. Given that there is only a small number of individuals who changed weight pattern groups, our data is mainly based on a small number of individuals even though the total data set is large.

## Conclusions

In summary, our study explored the temporal bidirectional relationship between adiposity and gut microbiota and identified ten microbes that were prospectively associated with baseline BMI. Three identified microbes (*Lachnospiraceae bacterium 3 1 57FAA CT1*, *Clostridium hathewayi*, and *Megamonas unclassified*) were associated with several insulin resistance-related phenotypes. We further discovered one potentially beneficial bacterium *Lachnospiraceae bacterium 3 1 57FAA CT1*, which might mediate the effect of adiposity on insulin resistance. These findings suggest that gut microbiota may lie in the pathway linking adiposity and insulin resistance, and the identified microbe *Lachnospiraceae bacterium 3 1 57FAA CT1* could be potentially served as a therapeutic target for insulin resistance.

## Supplementary Information

**Additional file 1: Table S1**. [Characteristics of the study participants between baseline and follow-up]. **Table S2**. [The temporal relationship between adiposity and α-diversity and β-diversity]. **Table S3**. [The temporal relationship between adiposity and gut microbes]. **Table S4**. [The correlation of identified microbes between two time points]. **Table S5**. [The differences in the associations of BMI with identified gut microbes between females and males]. **Table S6**. [The temporal relationship between WC and α-diversity and β-diversity]. **Table S7**. [The temporal relationship between WC and gut microbes]. **Table S8**. [The prospective associations between dietary factors and identified microbes]. **Table S9**. [Replication of the associations between baseline BMI and follow-up microbes in the HMP cohort]. **Table S10**. [The association between gut microbes and insulin resistance related phenotypes]. **Table S11**. [The prospective associations between dietary factors and insulin resistance related phenotypes]. **Table S12**. [The prospective association between weight group and insulin resistance related phenotypes]. **Table S13**. [The association between *Lachnospiraceae bacterium 3 1 57FAA CT1* and PWY-5022 pathway]. **Table S14**. [The associations between *Lachnospiraceae bacterium 3 1 57FAA CT1* and other pathways apart from PWY-5022]. **Figure S1**. [The associations between BMI/long-term weight change and gut microbiota]. **Figure S2**. [The associations between BMI/long-term weight change and α-diversity further adjusting for sequencing depths]. **Figure S3**. [The correlations among identified BMI-associated microbes]. **Figure S4**. [The correlation between the regression coefficients for females and those for males]. **Figure S5**. [Global comparison of 1-SD difference of BMI versus WC associated with difference (in SD unit) of all gut microbial features and ten identified gut microbes].

## Data Availability

The raw data of metagenomic sequencing from the GNHS are available in the CNSA (https://db.cngb.org/cnsa/) of CNGBdb at accession number CNP0001510. The raw metagenomic sequencing data of the Human Microbiome Project is available via http://www.hmpdacc.org. The metadata of the Human Microbiome Project is obtained by applying from the dbGaP (https://dbgap.ncbi.nlm.nih.gov/). Other datasets used and/or analyzed during the current study are available from the corresponding author on reasonable request.

## Abbreviations

ACME: Average causal mediation effects
ADE: Average direct effect
BH: Benjamini-Hochberg
CFI: Comparative fit index
CI: Confidence interval
FDR: False discovery rate
HbA1c: Hemoglobin A1c
HMP: Human microbiome project
HOMA-IR: Homeostasis model assessment of insulin resistance
MET: Metabolic equivalent for task
PCo: Principal coordinates
Q1: Quartile 1
Q3: Quartile3
SD: Standard deviation
SE: Standard error
SRMR: Standardized root mean square residual
WC: Waist circumference
