# Supplementary material for: Temporal relationship among adiposity, gut microbiota, and insulin resistance in a longitudinal human cohort
Source: BMC Med. 2022 May 19;20:171. doi: 10.1186/s12916-022-02376-3 (PMC9118787; doi:10.1186/s12916-022-02376-3)

# Supplementary tables

# Table S1. Characteristics of the study participants between baseline and follow-up*

| **Characteristics** | **Baseline (n=426)** | **Follow-up (n=426)** | ***P*** |
| --- | --- | --- | --- |
| Age, years, mean (SD) | 63.6 (5.4) | 66.8 (5.4) | <0.001 |
| Male, *n* (%) | 136 (31.9) |  |  |
| Female, *n* (%) | 290 (68.1) |  |  |
| BMI, kg/m^2^, mean (SD) | 23.4 (3.1) | 23.6 (3.3) | 0.002 |
| Adiposity (overweight or obesity), *n* (%) | 177 (41.5) | 179 (42) | <0.001 |
| Waist circumference, cm, mean (SD) | 85.3 (8.8) | 84.9 (9.0) | 0.116 |
| Current smoker, *n* (%) | 31 (7.3) | 29 (6.8) | 0.724 |
| Current alcohol drinker, *n* (%) | 35 (8.2) | 30 (7.0) | 0.441 |
| Education, *n* (%) |  |  |  |
| Middle school or lower | 100 (23.5) |  |  |
| High school or professional college | 204 (47.9) |  |  |
| University | 122 (28.6) |  |  |
| Income level, *n* (%) |  |  |  |
| Extremely Low (≤500 ¥/month) | 5 (1.2%) |  |  |
| Low (501-1500 ¥/month) | 90 (21.1) |  |  |
| Middle (1501-3000 ¥/month) | 273 (64.1) |  |  |
| High (>3000 ¥/month) | 58 (13.6) |  |  |
| Fasting Insulin, μU/mL, median (Q1, Q3) | 6.6 (4.4, 9.6) | 8.3 (5.9, 11.7) | <0.001 |
| HOMA-IR, median (Q1, Q3) | 1.5 (1.0, 2.2) | 2.0 (1.3, 2.9) | <0.001 |
| HbA1c, %, mean (SD) | 5.6 (0.4) | 5.7 (0.6) | <0.001 |
| Fasting glucose, mmol/L, median (Q1, Q3) | 5.1 (4.8, 5.5) | 5.4 (5.1, 5.8) | <0.001 |
| Physical activity, MET, mean (SD) | 40.8 (13.7) |  |  |
| Total energy intake, kcal/day, mean (SD) | 1760.6 (459.7) |  |  |

*Data are expressed as mean (SD) and median (Q1, Q3) for continuous variables with normal and skewed distribution, respectively, and as frequency (percentage) for categorical variables. The differences of participant characteristics between baseline and follow-up were tested using paired *t* test and Wilcoxon signed rank test for continuous variables with normal and skewed distribution, respectively, and using the Chi-squared test for categorical variables. SD, standard deviation；Q1, quartile 1; Q3, quartile3; HOMA-IR, homeostasis model assessment of insulin resistance; HbA1c, hemoglobin A1c; MET, metabolic equivalent for task.

# Table S2. The temporal relationship between adiposity and α-diversity and β-diversity*

| **Direction** | **ρ_1_** | **CI**  **lower** | **CI**  **upper** | ***p*** | **Direction** | **ρ_2_** | **CI**  **lower** | **CI**  **upper** | ***p*** | **CFI** | **SRMR** |
| --- | --- | --- | --- | --- | --- | --- | --- | --- | --- | --- | --- |
| Observed species on BMI | -0.01 | -0.05 | 0.02 | 0.492 | BMI on Observed species | 0.06 | -0.03 | 0.14 | 0.208 | 1 | 4.84929E-09 |
| Shannon index on BMI | 0 | -0.04 | 0.03 | 0.800 | BMI on Shannon index | 0.01 | -0.09 | 0.1 | 0.874 | 1 | 2.51283E-09 |
| Simpson index on BMI | -0.01 | -0.04 | 0.03 | 0.781 | BMI on Simpson index | -0.01 | -0.11 | 0.08 | 0.792 | 1 | 1.59322E-09 |
| Pielou's evenness on BMI | 0 | -0.04 | 0.03 | 0.828 | BMI on Pielou's evenness | -0.01 | -0.11 | 0.08 | 0.807 | 1 | 1.11108E-08 |
| PCo1 on BMI | 0 | -0.04 | 0.04 | 0.894 | BMI on PCo1 | 0 | -0.09 | 0.08 | 0.914 | 1 | 1.30209E-09 |
| PCo2 on BMI | 0 | -0.03 | 0.04 | 0.873 | BMI on PCo2 | -0.07 | -0.16 | 0.02 | 0.137 | 1 | 1.09204E-08 |

*The cross-lagged path analysis was used to estimate the difference in α- and β-diversity (in SD unit) per 1-SD difference in BMI, and the difference in BMI (in SD unit) per 1-SD difference in α- and β-diversity, adjusted for age, sex, smoking status, alcohol status, education, income, physical activity, total energy intake, Bristol stool score and time interval. PCo,Principal coordinates; CI, confidence interval; CFI, comparative fit index; SRMR, standardized root mean square residual.

# Table S3. The temporal relationship between adiposity and gut microbes*

| **direction** | **ρ_1_** | **CI**  **lower** | **CI**  **upper** | ***p*** | **FDR** | **direction** | **ρ_2_** | **CI**  **lower** | **CI**  **upper** | ***p*** | **FDR** | **CFI** | **SRMR** |
| --- | --- | --- | --- | --- | --- | --- | --- | --- | --- | --- | --- | --- | --- |
| Clostridium hathewayi on BMI | 0 | -0.04 | 0.03 | 0.836 | 0.994 | BMI on Clostridium hathewayi | -0.16 | -0.26 | -0.07 | 0.000 | ***0.077*** | 1 | 2.60917E-09 |
| Megasphaera unclassified on BMI | 0.01 | -0.03 | 0.05 | 0.611 | 0.994 | BMI on Megasphaera unclassified | 0.14 | 0.05 | 0.22 | 0.002 | ***0.093*** | 1 | 2.88053E-09 |
| Adlercreutzia equolifaciens on BMI | 0 | -0.04 | 0.04 | 0.931 | 0.994 | BMI on Adlercreutzia equolifaciens | 0.14 | 0.05 | 0.23 | 0.001 | ***0.093*** | 1 | 1.25979E-09 |
| Parabacteroides unclassified on BMI | -0.02 | -0.06 | 0.02 | 0.306 | 0.994 | BMI on Parabacteroides unclassified | -0.12 | -0.2 | -0.03 | 0.009 | ***0.202*** | 1 | 1.00729E-08 |
| Lachnospiraceae bacterium 3 1 57FAA CT1 on BMI | 0 | -0.04 | 0.04 | 0.989 | 0.994 | BMI on Lachnospiraceae bacterium 3 1 57FAA CT1 | -0.12 | -0.21 | -0.03 | 0.012 | ***0.202*** | 1 | 5.39677E-09 |
| Lachnospiraceae bacterium 7 1 58FAA on BMI | 0 | -0.04 | 0.04 | 0.986 | 0.994 | BMI on Lachnospiraceae bacterium 7 1 58FAA | -0.12 | -0.21 | -0.03 | 0.009 | ***0.202*** | 1 | 1.39543E-09 |
| Ruminococcus sp 5 1 39BFAA on BMI | -0.01 | -0.04 | 0.03 | 0.762 | 0.994 | BMI on Ruminococcus sp 5 1 39BFAA | 0.12 | 0.03 | 0.21 | 0.011 | ***0.202*** | 1 | 1.07551E-08 |
| Megamonas hypermegale on BMI | 0.01 | -0.03 | 0.05 | 0.596 | 0.994 | BMI on Megamonas hypermegale | 0.11 | 0.02 | 0.19 | 0.012 | ***0.202*** | 1 | 4.01927E-09 |
| Megamonas unclassified on BMI | 0.03 | -0.01 | 0.06 | 0.161 | 0.994 | BMI on Megamonas unclassified | 0.11 | 0.03 | 0.2 | 0.008 | ***0.202*** | 1 | 4.4004E-09 |
| Bacteroides caccae on BMI | -0.02 | -0.06 | 0.02 | 0.302 | 0.994 | BMI on Bacteroides caccae | 0.11 | 0.03 | 0.19 | 0.010 | ***0.202*** | 1 | 1.88336E-09 |
| Blautia producta on BMI | -0.01 | -0.05 | 0.03 | 0.667 | 0.994 | BMI on Blautia producta | -0.11 | -0.2 | -0.02 | 0.021 | 0.277 | 1 | 4.66869E-09 |
| Coprococcus sp ART55 1 on BMI | -0.01 | -0.04 | 0.03 | 0.774 | 0.994 | BMI on Coprococcus sp ART55 1 | -0.11 | -0.19 | -0.02 | 0.020 | 0.277 | 1 | 1.14728E-09 |
| Collinsella tanakaei on BMI | -0.02 | -0.06 | 0.01 | 0.231 | 0.994 | BMI on Collinsella tanakaei | 0.1 | 0.02 | 0.18 | 0.018 | 0.277 | 1 | 3.01471E-09 |
| Odoribacter splanchnicus on BMI | 0 | -0.04 | 0.04 | 0.930 | 0.994 | BMI on Odoribacter splanchnicus | 0.1 | 0.01 | 0.18 | 0.025 | 0.300 | 1 | 1.32833E-09 |
| Clostridium citroniae on BMI | -0.02 | -0.06 | 0.02 | 0.355 | 0.994 | BMI on Clostridium citroniae | -0.1 | -0.19 | 0 | 0.039 | 0.412 | 1 | 4.50137E-09 |
| Bifidobacterium pseudocatenulatum on BMI | 0.02 | -0.01 | 0.06 | 0.222 | 0.994 | BMI on Bifidobacterium pseudocatenulatum | 0.1 | 0.01 | 0.19 | 0.037 | 0.412 | 1 | 7.09504E-09 |
| Paraprevotella clara on BMI | -0.01 | -0.04 | 0.03 | 0.700 | 0.994 | BMI on Paraprevotella clara | 0.06 | 0 | 0.12 | 0.042 | 0.415 | 1 | 3.29519E-10 |
| Alistipes onderdonkii on BMI | 0 | -0.04 | 0.04 | 0.988 | 0.994 | BMI on Alistipes onderdonkii | 0.08 | 0 | 0.15 | 0.045 | 0.415 | 1 | 6.15245E-09 |
| Scardovia wiggsiae on BMI | -0.01 | -0.04 | 0.03 | 0.754 | 0.994 | BMI on Scardovia wiggsiae | -0.09 | -0.19 | 0 | 0.046 | 0.415 | 1 | 1.2065E-09 |
| Bacteroides ovatus on BMI | 0 | -0.04 | 0.03 | 0.858 | 0.994 | BMI on Bacteroides ovatus | -0.09 | -0.18 | 0.01 | 0.068 | 0.435 | 1 | 1.19638E-08 |
| Bacteroides thetaiotaomicron on BMI | -0.03 | -0.07 | 0.01 | 0.146 | 0.994 | BMI on Bacteroides thetaiotaomicron | -0.08 | -0.18 | 0.01 | 0.076 | 0.435 | 1 | 5.52607E-09 |
| Streptococcus mitis oralis pneumoniae on BMI | 0 | -0.04 | 0.03 | 0.889 | 0.994 | BMI on Streptococcus mitis oralis pneumoniae | 0.08 | -0.01 | 0.18 | 0.071 | 0.435 | 1 | 1.06571E-09 |
| Streptococcus vestibularis on BMI | -0.02 | -0.06 | 0.02 | 0.269 | 0.994 | BMI on Streptococcus vestibularis | 0.09 | 0 | 0.18 | 0.060 | 0.435 | 1 | 1.44977E-09 |
| Eubacterium ramulus on BMI | -0.03 | -0.07 | 0.01 | 0.102 | 0.994 | BMI on Eubacterium ramulus | 0.08 | -0.01 | 0.16 | 0.076 | 0.435 | 1 | 2.1924E-10 |
| Ruminococcus callidus on BMI | 0.03 | -0.01 | 0.06 | 0.157 | 0.994 | BMI on Ruminococcus callidus | 0.09 | 0 | 0.18 | 0.056 | 0.435 | 1 | 4.84561E-10 |
| Acidaminococcus intestini on BMI | 0 | -0.03 | 0.04 | 0.880 | 0.994 | BMI on Acidaminococcus intestini | 0.08 | 0 | 0.17 | 0.062 | 0.435 | 1 | 2.12445E-09 |
| Megamonas funiformis on BMI | 0.01 | -0.03 | 0.05 | 0.596 | 0.994 | BMI on Megamonas funiformis | 0.08 | -0.01 | 0.16 | 0.074 | 0.435 | 1 | 1.07737E-08 |
| Megamonas rupellensis on BMI | 0.03 | -0.01 | 0.07 | 0.095 | 0.994 | BMI on Megamonas rupellensis | 0.08 | -0.01 | 0.17 | 0.073 | 0.435 | 1 | 1.44263E-08 |
| Fusobacterium varium on BMI | 0.02 | -0.02 | 0.06 | 0.239 | 0.994 | BMI on Fusobacterium varium | -0.09 | -0.18 | 0 | 0.057 | 0.435 | 1 | 7.00869E-09 |
| Burkholderiales bacterium 1 1 47 on BMI | -0.01 | -0.04 | 0.03 | 0.745 | 0.994 | BMI on Burkholderiales bacterium 1 1 47 | 0.08 | -0.01 | 0.17 | 0.076 | 0.435 | 1 | 2.54096E-09 |
| Parabacteroides merdae on BMI | -0.01 | -0.04 | 0.03 | 0.730 | 0.994 | BMI on Parabacteroides merdae | 0.07 | -0.01 | 0.15 | 0.085 | 0.439 | 1 | 2.37377E-09 |
| Paraprevotella unclassified on BMI | -0.01 | -0.05 | 0.03 | 0.693 | 0.994 | BMI on Paraprevotella unclassified | 0.05 | -0.01 | 0.12 | 0.083 | 0.439 | 1 | 3.49441E-09 |
| Clostridium bolteae on BMI | 0 | -0.04 | 0.03 | 0.870 | 0.994 | BMI on Clostridium bolteae | -0.08 | -0.16 | 0.01 | 0.084 | 0.439 | 1 | 3.71998E-09 |
| Clostridium symbiosum on BMI | 0.01 | -0.03 | 0.05 | 0.641 | 0.994 | BMI on Clostridium symbiosum | -0.08 | -0.17 | 0.01 | 0.100 | 0.489 | 1 | 9.78858E-10 |
| Roseburia inulinivorans on BMI | -0.01 | -0.05 | 0.02 | 0.453 | 0.994 | BMI on Roseburia inulinivorans | 0.08 | -0.01 | 0.17 | 0.100 | 0.489 | 1 | 4.22932E-09 |
| Clostridium asparagiforme on BMI | -0.01 | -0.04 | 0.03 | 0.728 | 0.994 | BMI on Clostridium asparagiforme | -0.07 | -0.16 | 0.02 | 0.126 | 0.563 | 1 | 1.17354E-08 |
| Coprococcus comes on BMI | -0.01 | -0.05 | 0.03 | 0.592 | 0.994 | BMI on Coprococcus comes | 0.06 | -0.02 | 0.15 | 0.129 | 0.563 | 1 | 1.9399E-08 |
| Lachnospiraceae bacterium 2 1 58FAA on BMI | 0.03 | -0.01 | 0.06 | 0.179 | 0.994 | BMI on Lachnospiraceae bacterium 2 1 58FAA | 0.07 | -0.02 | 0.17 | 0.127 | 0.563 | 1 | 5.2771E-09 |
| Holdemania filiformis on BMI | 0.01 | -0.03 | 0.05 | 0.649 | 0.994 | BMI on Holdemania filiformis | 0.07 | -0.02 | 0.16 | 0.127 | 0.563 | 1 | 7.61432E-10 |
| Granulicatella unclassified on BMI | -0.02 | -0.06 | 0.02 | 0.280 | 0.994 | BMI on Granulicatella unclassified | 0.07 | -0.02 | 0.16 | 0.142 | 0.608 | 1 | 8.34199E-10 |
| Eubacterium ventriosum on BMI | -0.04 | -0.07 | 0 | 0.053 | 0.994 | BMI on Eubacterium ventriosum | 0.07 | -0.02 | 0.16 | 0.150 | 0.615 | 1 | 9.78087E-09 |
| Veillonella unclassified on BMI | 0.01 | -0.03 | 0.05 | 0.651 | 0.994 | BMI on Veillonella unclassified | -0.06 | -0.15 | 0.02 | 0.151 | 0.615 | 1 | 4.10932E-09 |
| Prevotella copri on BMI | 0.01 | -0.03 | 0.05 | 0.533 | 0.994 | BMI on Prevotella copri | 0.06 | -0.02 | 0.13 | 0.157 | 0.622 | 1 | 1.37534E-09 |
| Bacteroides coprocola on BMI | -0.01 | -0.05 | 0.03 | 0.700 | 0.994 | BMI on Bacteroides coprocola | -0.05 | -0.13 | 0.02 | 0.168 | 0.651 | 1 | 3.62577E-10 |
| Roseburia unclassified on BMI | 0.02 | -0.02 | 0.05 | 0.430 | 0.994 | BMI on Roseburia unclassified | -0.07 | -0.16 | 0.03 | 0.172 | 0.652 | 1 | 2.67864E-09 |
| Flavonifractor plautii on BMI | -0.01 | -0.05 | 0.03 | 0.566 | 0.994 | BMI on Flavonifractor plautii | -0.06 | -0.15 | 0.03 | 0.178 | 0.657 | 1 | 2.86145E-09 |
| Ruminococcus gnavus on BMI | 0 | -0.03 | 0.04 | 0.873 | 0.994 | BMI on Ruminococcus gnavus | -0.06 | -0.14 | 0.03 | 0.181 | 0.657 | 1 | 4.89873E-09 |
| Eubacterium hallii on BMI | -0.01 | -0.04 | 0.03 | 0.745 | 0.994 | BMI on Eubacterium hallii | 0.06 | -0.03 | 0.15 | 0.193 | 0.689 | 1 | 2.72322E-09 |
| Bacteroides finegoldii on BMI | -0.01 | -0.05 | 0.03 | 0.635 | 0.994 | BMI on Bacteroides finegoldii | 0.04 | -0.05 | 0.12 | 0.391 | 0.736 | 1 | 2.41052E-09 |
| Bacteroides salyersiae on BMI | -0.01 | -0.05 | 0.03 | 0.627 | 0.994 | BMI on Bacteroides salyersiae | 0.04 | -0.04 | 0.11 | 0.353 | 0.736 | 1 | 1.96425E-09 |
| Bacteroides uniformis on BMI | 0.02 | -0.02 | 0.05 | 0.406 | 0.994 | BMI on Bacteroides uniformis | -0.04 | -0.14 | 0.05 | 0.346 | 0.736 | 1 | 5.34558E-09 |
| Bacteroidales bacterium ph8 on BMI | -0.03 | -0.06 | 0.01 | 0.152 | 0.994 | BMI on Bacteroidales bacterium ph8 | 0.04 | -0.04 | 0.12 | 0.304 | 0.736 | 1 | 9.98186E-09 |
| Barnesiella intestinihominis on BMI | 0 | -0.04 | 0.04 | 0.946 | 0.994 | BMI on Barnesiella intestinihominis | 0.03 | -0.04 | 0.11 | 0.412 | 0.736 | 1 | 1.65723E-09 |
| Parabacteroides distasonis on BMI | 0.02 | -0.01 | 0.06 | 0.195 | 0.994 | BMI on Parabacteroides distasonis | 0.04 | -0.05 | 0.13 | 0.363 | 0.736 | 1 | 3.76374E-10 |
| Actinomyces odontolyticus on BMI | 0 | -0.04 | 0.03 | 0.860 | 0.994 | BMI on Actinomyces odontolyticus | -0.05 | -0.15 | 0.04 | 0.272 | 0.736 | 1 | 2.23101E-09 |
| Paraprevotella xylaniphila on BMI | 0 | -0.04 | 0.04 | 0.893 | 0.994 | BMI on Paraprevotella xylaniphila | 0.03 | -0.03 | 0.1 | 0.283 | 0.736 | 1 | 5.70973E-10 |
| Alistipes finegoldii on BMI | -0.03 | -0.06 | 0.01 | 0.173 | 0.994 | BMI on Alistipes finegoldii | -0.04 | -0.11 | 0.03 | 0.295 | 0.736 | 1 | 3.61031E-09 |
| Alistipes senegalensis on BMI | 0 | -0.03 | 0.04 | 0.796 | 0.994 | BMI on Alistipes senegalensis | -0.04 | -0.12 | 0.05 | 0.397 | 0.736 | 1 | 7.44931E-09 |
| Alistipes shahii on BMI | 0 | -0.04 | 0.04 | 0.948 | 0.994 | BMI on Alistipes shahii | -0.04 | -0.12 | 0.05 | 0.397 | 0.736 | 1 | 4.10174E-09 |
| Alistipes unclassified on BMI | 0.03 | -0.01 | 0.06 | 0.166 | 0.994 | BMI on Alistipes unclassified | -0.04 | -0.13 | 0.05 | 0.349 | 0.736 | 1 | 9.25055E-10 |
| Streptococcus parasanguinis on BMI | -0.03 | -0.07 | 0.01 | 0.147 | 0.994 | BMI on Streptococcus parasanguinis | 0.05 | -0.03 | 0.13 | 0.245 | 0.736 | 1 | 3.80029E-09 |
| Clostridiaceae bacterium JC118 on BMI | 0 | -0.04 | 0.04 | 0.966 | 0.994 | BMI on Clostridiaceae bacterium JC118 | -0.06 | -0.15 | 0.03 | 0.222 | 0.736 | 1 | 2.95782E-10 |
| Clostridium clostridioforme on BMI | 0.03 | -0.01 | 0.06 | 0.173 | 0.994 | BMI on Clostridium clostridioforme | -0.05 | -0.14 | 0.04 | 0.303 | 0.736 | 1 | 2.56769E-09 |
| Clostridium sp ATCC BAA 442 on BMI | 0.01 | -0.03 | 0.05 | 0.641 | 0.994 | BMI on Clostridium sp ATCC BAA 442 | -0.05 | -0.15 | 0.04 | 0.255 | 0.736 | 1 | 2.12E-08 |
| Clostridiales bacterium 1 7 47FAA on BMI | 0 | -0.03 | 0.04 | 0.810 | 0.994 | BMI on Clostridiales bacterium 1 7 47FAA | -0.05 | -0.15 | 0.04 | 0.246 | 0.736 | 1 | 1.40497E-08 |
| Eubacterium rectale on BMI | -0.01 | -0.04 | 0.03 | 0.731 | 0.994 | BMI on Eubacterium rectale | 0.04 | -0.05 | 0.13 | 0.385 | 0.736 | 1 | 5.58554E-09 |
| Eubacterium siraeum on BMI | 0 | -0.04 | 0.04 | 0.953 | 0.994 | BMI on Eubacterium siraeum | -0.03 | -0.12 | 0.05 | 0.430 | 0.736 | 1 | 4.70401E-09 |
| Anaerostipes unclassified on BMI | 0.02 | -0.02 | 0.05 | 0.420 | 0.994 | BMI on Anaerostipes unclassified | -0.05 | -0.14 | 0.05 | 0.330 | 0.736 | 1 | 9.3597E-09 |
| Coprococcus catus on BMI | 0 | -0.04 | 0.04 | 0.942 | 0.994 | BMI on Coprococcus catus | 0.04 | -0.05 | 0.12 | 0.381 | 0.736 | 1 | 1.27949E-08 |
| Dorea longicatena on BMI | -0.02 | -0.06 | 0.02 | 0.358 | 0.994 | BMI on Dorea longicatena | 0.05 | -0.04 | 0.14 | 0.296 | 0.736 | 1 | 3.20662E-09 |
| Lachnospiraceae bacterium 1 1 57FAA on BMI | 0.01 | -0.03 | 0.05 | 0.615 | 0.994 | BMI on Lachnospiraceae bacterium 1 1 57FAA | 0.04 | -0.05 | 0.13 | 0.410 | 0.736 | 1 | 3.35442E-09 |
| Lachnospiraceae bacterium 3 1 46FAA on BMI | -0.02 | -0.05 | 0.02 | 0.398 | 0.994 | BMI on Lachnospiraceae bacterium 3 1 46FAA | 0.04 | -0.05 | 0.13 | 0.383 | 0.736 | 1 | 7.42714E-10 |
| Roseburia hominis on BMI | 0 | -0.04 | 0.04 | 0.943 | 0.994 | BMI on Roseburia hominis | 0.05 | -0.04 | 0.14 | 0.298 | 0.736 | 1 | 3.38095E-09 |
| Roseburia intestinalis on BMI | 0.01 | -0.03 | 0.04 | 0.746 | 0.994 | BMI on Roseburia intestinalis | -0.04 | -0.13 | 0.05 | 0.363 | 0.736 | 1 | 1.23345E-09 |
| Anaerotruncus colihominis on BMI | 0 | -0.04 | 0.03 | 0.854 | 0.994 | BMI on Anaerotruncus colihominis | -0.04 | -0.13 | 0.05 | 0.424 | 0.736 | 1 | 6.85719E-10 |
| Anaerotruncus unclassified on BMI | -0.02 | -0.06 | 0.02 | 0.318 | 0.994 | BMI on Anaerotruncus unclassified | -0.04 | -0.13 | 0.06 | 0.422 | 0.736 | 1 | 3.45053E-09 |
| Subdoligranulum sp 4 3 54A2FAA on BMI | -0.03 | -0.07 | 0 | 0.080 | 0.994 | BMI on Subdoligranulum sp 4 3 54A2FAA | -0.05 | -0.14 | 0.04 | 0.245 | 0.736 | 1 | 2.95007E-09 |
| Clostridium ramosum on BMI | 0 | -0.04 | 0.04 | 0.900 | 0.994 | BMI on Clostridium ramosum | 0.04 | -0.05 | 0.13 | 0.429 | 0.736 | 1 | 4.89055E-09 |
| Eubacterium biforme on BMI | 0 | -0.04 | 0.04 | 0.994 | 0.994 | BMI on Eubacterium biforme | 0.04 | -0.03 | 0.12 | 0.261 | 0.736 | 1 | 3.44104E-09 |
| Holdemania unclassified on BMI | 0 | -0.04 | 0.04 | 0.901 | 0.994 | BMI on Holdemania unclassified | -0.04 | -0.13 | 0.05 | 0.409 | 0.736 | 1 | 4.14651E-09 |
| Acidaminococcus unclassified on BMI | 0.01 | -0.03 | 0.04 | 0.738 | 0.994 | BMI on Acidaminococcus unclassified | 0.05 | -0.03 | 0.13 | 0.248 | 0.736 | 1 | 1.3502E-09 |
| Veillonella dispar on BMI | -0.02 | -0.06 | 0.02 | 0.315 | 0.994 | BMI on Veillonella dispar | 0.04 | -0.05 | 0.12 | 0.368 | 0.736 | 1 | 4.63848E-09 |
| Veillonella parvula on BMI | -0.01 | -0.05 | 0.02 | 0.510 | 0.994 | BMI on Veillonella parvula | -0.04 | -0.12 | 0.05 | 0.414 | 0.736 | 1 | 6.28455E-09 |
| Fusobacterium mortiferum on BMI | 0.01 | -0.02 | 0.05 | 0.488 | 0.994 | BMI on Fusobacterium mortiferum | 0.04 | -0.05 | 0.13 | 0.358 | 0.736 | 1 | 9.28501E-09 |
| Fusobacterium ulcerans on BMI | 0.01 | -0.03 | 0.04 | 0.708 | 0.994 | BMI on Fusobacterium ulcerans | -0.05 | -0.14 | 0.05 | 0.315 | 0.736 | 1 | 4.20956E-09 |
| Parasutterella excrementihominis on BMI | 0 | -0.04 | 0.03 | 0.810 | 0.994 | BMI on Parasutterella excrementihominis | 0.05 | -0.05 | 0.14 | 0.332 | 0.736 | 1 | 6.99618E-10 |
| Sutterella wadsworthensis on BMI | 0.01 | -0.03 | 0.05 | 0.538 | 0.994 | BMI on Sutterella wadsworthensis | 0.04 | -0.04 | 0.12 | 0.337 | 0.736 | 1 | 4.08217E-09 |
| Bilophila unclassified on BMI | 0.01 | -0.02 | 0.05 | 0.448 | 0.994 | BMI on Bilophila unclassified | 0.04 | -0.05 | 0.13 | 0.419 | 0.736 | 1 | 3.22008E-09 |
| Citrobacter freundii on BMI | 0 | -0.04 | 0.03 | 0.813 | 0.994 | BMI on Citrobacter freundii | -0.05 | -0.15 | 0.04 | 0.275 | 0.736 | 1 | 1.65485E-09 |
| Enterobacter cloacae on BMI | 0 | -0.03 | 0.04 | 0.858 | 0.994 | BMI on Enterobacter cloacae | -0.04 | -0.13 | 0.05 | 0.426 | 0.736 | 1 | 1.76963E-09 |
| Escherichia coli on BMI | 0 | -0.03 | 0.04 | 0.874 | 0.994 | BMI on Escherichia coli | -0.04 | -0.13 | 0.05 | 0.368 | 0.736 | 1 | 4.77236E-10 |
| Bifidobacterium bifidum on BMI | 0.03 | -0.01 | 0.07 | 0.137 | 0.994 | BMI on Bifidobacterium bifidum | 0.05 | -0.05 | 0.14 | 0.315 | 0.736 | 1 | 5.84892E-09 |
| Klebsiella pneumoniae on BMI | 0.02 | -0.02 | 0.05 | 0.376 | 0.994 | BMI on Klebsiella pneumoniae | -0.05 | -0.14 | 0.04 | 0.294 | 0.736 | 1 | 1.02966E-09 |
| Haemophilus parainfluenzae on BMI | 0.01 | -0.02 | 0.05 | 0.456 | 0.994 | BMI on Haemophilus parainfluenzae | -0.04 | -0.13 | 0.04 | 0.334 | 0.736 | 1 | 6.66477E-09 |
| Pyramidobacter piscolens on BMI | 0 | -0.04 | 0.04 | 0.940 | 0.994 | BMI on Pyramidobacter piscolens | 0.06 | -0.04 | 0.15 | 0.233 | 0.736 | 1 | 6.9417E-10 |
| Akkermansia muciniphila on BMI | -0.02 | -0.06 | 0.02 | 0.293 | 0.994 | BMI on Akkermansia muciniphila | 0.04 | -0.05 | 0.13 | 0.410 | 0.736 | 1 | 7.00232E-09 |
| Atopobium parvulum on BMI | -0.01 | -0.05 | 0.02 | 0.440 | 0.994 | BMI on Atopobium parvulum | 0.05 | -0.04 | 0.14 | 0.290 | 0.736 | 1 | 2.34442E-09 |
| Collinsella aerofaciens on BMI | -0.02 | -0.06 | 0.02 | 0.353 | 0.994 | BMI on Collinsella aerofaciens | 0.05 | -0.03 | 0.14 | 0.214 | 0.736 | 1 | 1.4811E-10 |
| Coriobacteriaceae bacterium phI on BMI | -0.02 | -0.06 | 0.02 | 0.350 | 0.994 | BMI on Coriobacteriaceae bacterium phI | -0.04 | -0.13 | 0.05 | 0.371 | 0.736 | 1 | 1.77972E-09 |
| Bacteroides coprophilus on BMI | 0.03 | -0.01 | 0.07 | 0.160 | 0.994 | BMI on Bacteroides coprophilus | -0.04 | -0.11 | 0.03 | 0.236 | 0.736 | 1 | 6.92764E-10 |
| Subdoligranulum unclassified on BMI | -0.01 | -0.05 | 0.03 | 0.692 | 0.994 | BMI on Subdoligranulum unclassified | -0.04 | -0.12 | 0.05 | 0.436 | 0.738 | 1 | 1.03677E-08 |
| Peptostreptococcaceae noname unclassified on BMI | 0.06 | 0.02 | 0.1 | 0.002 | 0.270 | BMI on Peptostreptococcaceae noname unclassified | 0.04 | -0.06 | 0.13 | 0.459 | 0.770 | 1 | 5.93436E-09 |
| Ruminococcus obeum on BMI | 0 | -0.04 | 0.04 | 0.919 | 0.994 | BMI on Ruminococcus obeum | 0.03 | -0.06 | 0.12 | 0.475 | 0.788 | 1 | 5.32832E-09 |
| Megasphaera micronuciformis on BMI | 0.01 | -0.02 | 0.05 | 0.479 | 0.994 | BMI on Megasphaera micronuciformis | -0.03 | -0.12 | 0.06 | 0.493 | 0.810 | 1 | 1.32605E-08 |
| Lachnospiraceae bacterium 1 4 56FAA on BMI | -0.02 | -0.06 | 0.02 | 0.262 | 0.994 | BMI on Lachnospiraceae bacterium 1 4 56FAA | 0.03 | -0.06 | 0.12 | 0.532 | 0.849 | 1 | 2.62254E-09 |
| Oscillibacter unclassified on BMI | -0.01 | -0.05 | 0.03 | 0.579 | 0.994 | BMI on Oscillibacter unclassified | -0.03 | -0.12 | 0.06 | 0.529 | 0.849 | 1 | 4.16497E-09 |
| Citrobacter unclassified on BMI | 0.01 | -0.03 | 0.05 | 0.660 | 0.994 | BMI on Citrobacter unclassified | -0.03 | -0.13 | 0.06 | 0.524 | 0.849 | 1 | 1.61012E-09 |
| Bacteroides fragilis on BMI | -0.02 | -0.06 | 0.02 | 0.276 | 0.994 | BMI on Bacteroides fragilis | -0.03 | -0.11 | 0.06 | 0.551 | 0.851 | 1 | 3.62918E-09 |
| Bacteroides massiliensis on BMI | 0.02 | -0.02 | 0.06 | 0.348 | 0.994 | BMI on Bacteroides massiliensis | -0.02 | -0.09 | 0.05 | 0.550 | 0.851 | 1 | 7.70723E-09 |
| Alistipes indistinctus on BMI | 0.02 | -0.02 | 0.06 | 0.290 | 0.994 | BMI on Alistipes indistinctus | -0.03 | -0.12 | 0.06 | 0.551 | 0.851 | 1 | 1.63961E-09 |
| Streptococcus anginosus on BMI | 0 | -0.04 | 0.04 | 0.932 | 0.994 | BMI on Streptococcus anginosus | 0.03 | -0.06 | 0.12 | 0.553 | 0.851 | 1 | 5.22382E-10 |
| Dorea formicigenerans on BMI | -0.02 | -0.06 | 0.02 | 0.339 | 0.994 | BMI on Dorea formicigenerans | 0.03 | -0.06 | 0.12 | 0.562 | 0.858 | 1 | 2.08009E-09 |
| Coprobacter fastidiosus on BMI | 0.05 | 0.01 | 0.09 | 0.006 | 0.539 | BMI on Coprobacter fastidiosus | 0.02 | -0.06 | 0.11 | 0.583 | 0.868 | 1 | 1.03086E-08 |
| Bacteroides faecis on BMI | -0.02 | -0.06 | 0.01 | 0.205 | 0.994 | BMI on Bacteroides faecis | 0.02 | -0.06 | 0.11 | 0.584 | 0.868 | 1 | 2.37799E-09 |
| Parabacteroides johnsonii on BMI | -0.04 | -0.08 | 0 | 0.030 | 0.994 | BMI on Parabacteroides johnsonii | -0.02 | -0.1 | 0.06 | 0.581 | 0.868 | 1 | 2.30718E-10 |
| Anaerostipes hadrus on BMI | 0.01 | -0.03 | 0.05 | 0.553 | 0.994 | BMI on Anaerostipes hadrus | 0.02 | -0.07 | 0.12 | 0.596 | 0.879 | 1 | 3.09496E-10 |
| Slackia piriformis on BMI | 0.01 | -0.03 | 0.05 | 0.678 | 0.994 | BMI on Slackia piriformis | 0.02 | -0.06 | 0.1 | 0.615 | 0.898 | 1 | 6.45196E-10 |
| Clostridium nexile on BMI | -0.02 | -0.06 | 0.02 | 0.308 | 0.994 | BMI on Clostridium nexile | 0.02 | -0.07 | 0.11 | 0.624 | 0.904 | 1 | 1.19903E-09 |
| Bacteroides stercoris on BMI | -0.02 | -0.06 | 0.02 | 0.303 | 0.994 | BMI on Bacteroides stercoris | -0.02 | -0.1 | 0.06 | 0.632 | 0.905 | 1 | 4.78283E-09 |
| Bacteroides cellulosilyticus on BMI | 0 | -0.03 | 0.04 | 0.874 | 0.994 | BMI on Bacteroides cellulosilyticus | -0.02 | -0.12 | 0.07 | 0.635 | 0.905 | 1 | 7.48821E-10 |
| Ruminococcus torques on BMI | -0.02 | -0.05 | 0.02 | 0.416 | 0.994 | BMI on Ruminococcus torques | -0.02 | -0.12 | 0.07 | 0.646 | 0.907 | 1 | 6.1843E-09 |
| Lachnospiraceae bacterium 9 1 43BFAA on BMI | -0.02 | -0.05 | 0.02 | 0.372 | 0.994 | BMI on Lachnospiraceae bacterium 9 1 43BFAA | 0.02 | -0.07 | 0.11 | 0.649 | 0.907 | 1 | 7.14866E-09 |
| Oxalobacter formigenes on BMI | 0 | -0.03 | 0.04 | 0.873 | 0.994 | BMI on Oxalobacter formigenes | -0.02 | -0.11 | 0.07 | 0.653 | 0.907 | 1 | 2.38667E-09 |
| Gordonibacter pamelaeae on BMI | 0 | -0.04 | 0.04 | 0.957 | 0.994 | BMI on Gordonibacter pamelaeae | 0.02 | -0.07 | 0.11 | 0.660 | 0.910 | 1 | 3.14289E-09 |
| Lachnospiraceae bacterium 5 1 57FAA on BMI | 0.02 | -0.02 | 0.06 | 0.253 | 0.994 | BMI on Lachnospiraceae bacterium 5 1 57FAA | 0.02 | -0.07 | 0.11 | 0.668 | 0.914 | 1 | 6.45102E-10 |
| Ruminococcus bromii on BMI | 0 | -0.03 | 0.04 | 0.870 | 0.994 | BMI on Ruminococcus bromii | -0.02 | -0.11 | 0.07 | 0.673 | 0.914 | 1 | 4.90159E-09 |
| Rothia mucilaginosa on BMI | 0.01 | -0.03 | 0.05 | 0.556 | 0.994 | BMI on Rothia mucilaginosa | -0.02 | -0.11 | 0.07 | 0.684 | 0.921 | 1 | 6.44062E-10 |
| Dorea unclassified on BMI | 0 | -0.04 | 0.04 | 0.891 | 0.994 | BMI on Dorea unclassified | -0.02 | -0.11 | 0.08 | 0.715 | 0.956 | 1 | 2.89917E-09 |
| Clostridium leptum on BMI | 0 | -0.03 | 0.04 | 0.891 | 0.994 | BMI on Clostridium leptum | 0.02 | -0.07 | 0.11 | 0.734 | 0.956 | 1 | 4.56808E-09 |
| Eubacterium eligens on BMI | 0 | -0.04 | 0.04 | 0.907 | 0.994 | BMI on Eubacterium eligens | 0.02 | -0.08 | 0.11 | 0.731 | 0.956 | 1 | 4.83115E-09 |
| Bifidobacterium adolescentis on BMI | -0.01 | -0.04 | 0.03 | 0.763 | 0.994 | BMI on Bifidobacterium adolescentis | 0.02 | -0.07 | 0.1 | 0.735 | 0.956 | 1 | 1.12826E-09 |
| Eggerthella unclassified on BMI | 0 | -0.04 | 0.04 | 0.980 | 0.994 | BMI on Eggerthella unclassified | -0.01 | -0.1 | 0.07 | 0.738 | 0.956 | 1 | 1.02684E-09 |
| Eubacterium limosum on BMI | -0.03 | -0.07 | 0.01 | 0.098 | 0.994 | BMI on Eubacterium limosum | 0.01 | -0.08 | 0.11 | 0.770 | 0.962 | 1 | 3.41144E-09 |
| Clostridium bartlettii on BMI | 0 | -0.03 | 0.04 | 0.815 | 0.994 | BMI on Clostridium bartlettii | -0.01 | -0.11 | 0.08 | 0.763 | 0.962 | 1 | 6.20652E-09 |
| Veillonella atypica on BMI | 0 | -0.03 | 0.04 | 0.854 | 0.994 | BMI on Veillonella atypica | 0.01 | -0.07 | 0.1 | 0.753 | 0.962 | 1 | 1.41996E-08 |
| Desulfovibrio desulfuricans on BMI | 0 | -0.04 | 0.03 | 0.892 | 0.994 | BMI on Desulfovibrio desulfuricans | -0.01 | -0.1 | 0.08 | 0.773 | 0.962 | 1 | 2.4584E-09 |
| Enterobacter aerogenes on BMI | 0 | -0.04 | 0.03 | 0.797 | 0.994 | BMI on Enterobacter aerogenes | 0.01 | -0.08 | 0.11 | 0.788 | 0.962 | 1 | 9.43766E-10 |
| Escherichia unclassified on BMI | -0.01 | -0.04 | 0.03 | 0.788 | 0.994 | BMI on Escherichia unclassified | -0.01 | -0.11 | 0.08 | 0.770 | 0.962 | 1 | 3.24041E-09 |
| Klebsiella unclassified on BMI | 0 | -0.04 | 0.04 | 0.987 | 0.994 | BMI on Klebsiella unclassified | -0.01 | -0.11 | 0.08 | 0.785 | 0.962 | 1 | 6.31349E-09 |
| Bacteroides clarus on BMI | -0.01 | -0.04 | 0.03 | 0.718 | 0.994 | BMI on Bacteroides clarus | 0.01 | -0.07 | 0.09 | 0.788 | 0.962 | 1 | 9.15816E-10 |
| Butyricimonas synergistica on BMI | 0.01 | -0.02 | 0.05 | 0.442 | 0.994 | BMI on Butyricimonas synergistica | 0.01 | -0.08 | 0.1 | 0.796 | 0.965 | 1 | 1.12705E-09 |
| Alistipes sp AP11 on BMI | -0.02 | -0.06 | 0.01 | 0.203 | 0.994 | BMI on Alistipes sp AP11 | 0.01 | -0.07 | 0.09 | 0.832 | 0.973 | 1 | 5.35076E-09 |
| Streptococcus infantis on BMI | 0 | -0.04 | 0.04 | 0.942 | 0.994 | BMI on Streptococcus infantis | -0.01 | -0.1 | 0.08 | 0.839 | 0.973 | 1 | 1.46274E-09 |
| Lachnospiraceae bacterium 5 1 63FAA on BMI | 0.01 | -0.03 | 0.05 | 0.640 | 0.994 | BMI on Lachnospiraceae bacterium 5 1 63FAA | 0.01 | -0.08 | 0.1 | 0.836 | 0.973 | 1 | 3.21944E-09 |
| Faecalibacterium prausnitzii on BMI | 0 | -0.04 | 0.04 | 0.916 | 0.994 | BMI on Faecalibacterium prausnitzii | 0.01 | -0.08 | 0.1 | 0.811 | 0.973 | 1 | 2.89222E-09 |
| Ruminococcaceae bacterium D16 on BMI | -0.03 | -0.06 | 0.01 | 0.179 | 0.994 | BMI on Ruminococcaceae bacterium D16 | -0.01 | -0.1 | 0.08 | 0.824 | 0.973 | 1 | 1.39917E-09 |
| Ruminococcus lactaris on BMI | 0 | -0.04 | 0.04 | 0.924 | 0.994 | BMI on Ruminococcus lactaris | -0.01 | -0.1 | 0.08 | 0.842 | 0.973 | 1 | 3.25547E-09 |
| Collinsella intestinalis on BMI | -0.01 | -0.04 | 0.03 | 0.749 | 0.994 | BMI on Collinsella intestinalis | -0.01 | -0.1 | 0.08 | 0.836 | 0.973 | 1 | 1.08566E-08 |
| Bacteroides dorei on BMI | -0.05 | -0.08 | -0.01 | 0.016 | 0.899 | BMI on Bacteroides dorei | -0.01 | -0.07 | 0.06 | 0.872 | 0.975 | 1 | 1.65443E-09 |
| Bacteroides eggerthii on BMI | 0 | -0.04 | 0.04 | 0.941 | 0.994 | BMI on Bacteroides eggerthii | 0 | -0.08 | 0.07 | 0.945 | 0.975 | 1 | 1.65402E-09 |
| Bacteroides intestinalis on BMI | -0.02 | -0.06 | 0.02 | 0.342 | 0.994 | BMI on Bacteroides intestinalis | 0 | -0.08 | 0.09 | 0.913 | 0.975 | 1 | 5.68393E-09 |
| Bacteroides nordii on BMI | -0.03 | -0.06 | 0.01 | 0.163 | 0.994 | BMI on Bacteroides nordii | -0.01 | -0.1 | 0.08 | 0.859 | 0.975 | 1 | 3.03612E-09 |
| Bacteroides plebeius on BMI | -0.02 | -0.05 | 0.02 | 0.432 | 0.994 | BMI on Bacteroides plebeius | 0 | -0.08 | 0.07 | 0.922 | 0.975 | 1 | 4.08391E-09 |
| Bacteroides vulgatus on BMI | 0 | -0.04 | 0.04 | 0.971 | 0.994 | BMI on Bacteroides vulgatus | -0.01 | -0.09 | 0.08 | 0.909 | 0.975 | 1 | 6.98351E-09 |
| Bacteroides xylanisolvens on BMI | 0 | -0.04 | 0.04 | 0.912 | 0.994 | BMI on Bacteroides xylanisolvens | 0 | -0.1 | 0.09 | 0.939 | 0.975 | 1 | 7.39786E-09 |
| Alistipes putredinis on BMI | -0.01 | -0.05 | 0.02 | 0.497 | 0.994 | BMI on Alistipes putredinis | 0.01 | -0.08 | 0.09 | 0.901 | 0.975 | 1 | 5.69077E-10 |
| Streptococcus gordonii on BMI | -0.02 | -0.06 | 0.01 | 0.229 | 0.994 | BMI on Streptococcus gordonii | 0.01 | -0.08 | 0.1 | 0.903 | 0.975 | 1 | 5.04386E-10 |
| Streptococcus sanguinis on BMI | 0 | -0.03 | 0.04 | 0.859 | 0.994 | BMI on Streptococcus sanguinis | 0.01 | -0.09 | 0.1 | 0.901 | 0.975 | 1 | 1.7952E-09 |
| Lachnospiraceae bacterium 8 1 57FAA on BMI | 0.02 | -0.02 | 0.05 | 0.419 | 0.994 | BMI on Lachnospiraceae bacterium 8 1 57FAA | 0.01 | -0.09 | 0.1 | 0.870 | 0.975 | 1 | 4.90017E-09 |
| Dialister invisus on BMI | -0.02 | -0.05 | 0.02 | 0.361 | 0.994 | BMI on Dialister invisus | 0 | -0.1 | 0.09 | 0.946 | 0.975 | 1 | 1.77603E-08 |
| Bilophila wadsworthia on BMI | -0.01 | -0.05 | 0.03 | 0.624 | 0.994 | BMI on Bilophila wadsworthia | 0 | -0.09 | 0.08 | 0.941 | 0.975 | 1 | 1.05519E-09 |
| Desulfovibrio piger on BMI | -0.02 | -0.06 | 0.01 | 0.213 | 0.994 | BMI on Desulfovibrio piger | 0.01 | -0.07 | 0.08 | 0.880 | 0.975 | 1 | 1.20635E-08 |
| Bifidobacterium dentium on BMI | -0.01 | -0.05 | 0.02 | 0.441 | 0.994 | BMI on Bifidobacterium dentium | -0.01 | -0.1 | 0.08 | 0.856 | 0.975 | 1 | 3.80963E-09 |
| Bifidobacterium longum on BMI | 0.01 | -0.03 | 0.05 | 0.598 | 0.994 | BMI on Bifidobacterium longum | 0 | -0.08 | 0.09 | 0.926 | 0.975 | 1 | 9.27932E-09 |
| Murine osteosarcoma virus on BMI | 0 | -0.04 | 0.04 | 0.949 | 0.994 | BMI on Murine osteosarcoma virus | 0.01 | -0.09 | 0.1 | 0.890 | 0.975 | 1 | 1.36914E-09 |
| Eggerthella lenta on BMI | -0.01 | -0.05 | 0.03 | 0.562 | 0.994 | BMI on Eggerthella lenta | -0.01 | -0.1 | 0.09 | 0.908 | 0.975 | 1 | 2.9635E-09 |
| Parabacteroides goldsteinii on BMI | -0.02 | -0.06 | 0.02 | 0.350 | 0.994 | BMI on Parabacteroides goldsteinii | 0 | -0.09 | 0.09 | 0.970 | 0.985 | 1 | 1.1811E-08 |
| Streptococcus australis on BMI | 0.02 | -0.01 | 0.06 | 0.236 | 0.994 | BMI on Streptococcus australis | 0 | -0.09 | 0.09 | 0.982 | 0.985 | 1 | 2.18768E-09 |
| Streptococcus salivarius on BMI | 0 | -0.03 | 0.04 | 0.851 | 0.994 | BMI on Streptococcus salivarius | 0 | -0.09 | 0.09 | 0.978 | 0.985 | 1 | 3.119E-09 |
| Streptococcus thermophilus on BMI | -0.02 | -0.06 | 0.02 | 0.305 | 0.994 | BMI on Streptococcus thermophilus | 0 | -0.1 | 0.09 | 0.964 | 0.985 | 1 | 4.92945E-09 |
| Coprobacillus unclassified on BMI | -0.01 | -0.05 | 0.03 | 0.558 | 0.994 | BMI on Coprobacillus unclassified | 0 | -0.09 | 0.09 | 0.985 | 0.985 | 1 | 5.4784E-09 |

*The cross-lagged path analysis was used to estimate the difference in the abundance of gut microbes (in SD unit of the log-transformed abundance) per 1-SD difference in BMI, and the difference in BMI (in SD unit) per 1-SD difference in the log-transformed abundance of gut microbes, adjusted for age, sex, smoking status, alcohol status, education, income, physical activity, total energy intake, Bristol stool score and time interval. CI, confidence interval; FDR, false discovery rate; CFI, comparative fit index; SRMR, standardized root mean square residual.

# Table S4. The correlation of identified microbes between two time points*

| **Taxonomy** | ***r*** | ***p*** | **FDR** |
| --- | --- | --- | --- |
| *Megamonas hypermegale* | 0.46 | 7.66E-24 | 7.66E-23 |
| *Megamonas unclassified* | 0.45 | 7.61E-23 | 3.80E-22 |
| *Adlercreutzia equolifaciens* | 0.43 | 7.18E-21 | 2.39E-20 |
| *Bacteroides caccae* | 0.42 | 7.65E-20 | 1.91E-19 |
| *Megasphaera unclassified* | 0.40 | 8.21E-18 | 1.64E-17 |
| *Parabacteroides unclassified* | 0.37 | 1.53E-15 | 2.55E-15 |
| *Lachnospiraceae bacterium 3 1 57FAA CT1* | 0.31 | 5.12E-11 | 7.32E-11 |
| *Lachnospiraceae bacterium 7 1 58FAA* | 0.29 | 1.20E-09 | 1.50E-09 |
| *Clostridium hathewayi* | 0.27 | 2.28E-08 | 2.53E-08 |
| *Ruminococcus sp 5 1 39BFAA* | 0.12 | 0.014885 | 0.014885 |

*The correlation of identified microbes between two time points were estimated using Spearman correlation analysis.

# Table S5. The differences in the associations of BMI with identified gut microbes between females and males*

| **Taxonomy** | **Female** | | | **Male** | | | ***p*_heterogeneity_** | **FDR_heterogeneity_** |
| --- | --- | --- | --- | --- | --- | --- | --- | --- |
|  | **Estimate** | **CI**  **lower** | **CI**  **upper** | **Estimate** | **CI**  **lower** | **CI**  **upper** |  |  |
| *Parabacteroides unclassified* | -0.08 | -0.19 | 0.02 | -0.14 | -0.3 | 0.01 | 0.527 | 0.982 |
| *Clostridium hathewayi* | -0.15 | -0.27 | -0.04 | -0.15 | -0.31 | 0.02 | 0.958 | 0.982 |
| *Lachnospiraceae bacterium 3 1 57FAA CT1* | -0.15 | -0.26 | -0.04 | -0.04 | -0.2 | 0.12 | 0.254 | 0.928 |
| *Lachnospiraceae bacterium 7 1 58FAA* | -0.09 | -0.2 | 0.02 | -0.18 | -0.34 | -0.02 | 0.371 | 0.928 |
| *Ruminococcus sp 5 1 39BFAA* | 0.05 | -0.07 | 0.16 | 0.28 | 0.12 | 0.43 | 0.02 | 0.195 |
| *Megamonas hypermegale* | 0.09 | -0.01 | 0.2 | 0.14 | 0 | 0.27 | 0.602 | 0.982 |
| *Megamonas unclassified* | 0.12 | 0.01 | 0.22 | 0.12 | -0.02 | 0.26 | 0.982 | 0.982 |
| *Megasphaera unclassified* | 0.14 | 0.03 | 0.24 | 0.12 | -0.04 | 0.28 | 0.825 | 0.982 |
| *Adlercreutzia equolifaciens* | 0.1 | 0 | 0.21 | 0.2 | 0.05 | 0.34 | 0.308 | 0.928 |
| *Bacteroides caccae* | 0.09 | -0.01 | 0.18 | 0.11 | -0.05 | 0.26 | 0.843 | 0.982 |

* The cross-lagged path analysis was used to estimate the difference in the abundance of identified gut microbes (in SD unit of the log-transformed abundance) per 1-SD difference in BMI for females and males, respectively, adjusted for age, smoking status, alcohol status, education, income, physical activity, total energy intake, Bristol stool score and time interval. The differences in the regression coefficients between females and males were tested using the Cochran-Q test. CI, confidence interval; FDR, false discovery rate.

# Table S6. The temporal relationship between WC and α-diversity and β-diversity*

| **Direction of effects** | **ρ_1_** | **CI**  **lower** | **CI**  **upper** | ***p*** | **Direction of effects** | **ρ_2_** | **CI**  **lower** | **CI**  **upper** | ***p*** | **CFI** | **SRMR** |
| --- | --- | --- | --- | --- | --- | --- | --- | --- | --- | --- | --- |
| Observed species on WC | 0.03 | -0.03 | 0.08 | 0.340 | WC on Observed species | 0.04 | -0.05 | 0.12 | 0.415 | 1 | 2.19709E-09 |
| Shannon index on WC | 0.02 | -0.03 | 0.08 | 0.454 | WC on Shannon index | 0.01 | -0.09 | 0.1 | 0.908 | 1 | 3.35106E-09 |
| Simpson index on WC | 0 | -0.06 | 0.05 | 0.972 | WC on Simpson index | -0.03 | -0.13 | 0.06 | 0.471 | 1 | 9.66263E-10 |
| Pielou's evenness on WC | 0.01 | -0.04 | 0.07 | 0.624 | WC on Pielou's evenness | -0.01 | -0.1 | 0.08 | 0.830 | 1 | 8.71976E-10 |
| PCo1 on WC | 0.01 | -0.04 | 0.07 | 0.631 | WC on PCo1 | -0.01 | -0.1 | 0.08 | 0.847 | 1 | 4.36646E-11 |
| PCo2 on WC | -0.03 | -0.09 | 0.02 | 0.275 | WC on PCo2 | -0.06 | -0.16 | 0.03 | 0.172 | 1 | 1.96017E-09 |

*The cross-lagged path analysis was used to estimate the difference in α- and β-diversity (in SD unit) per 1-SD difference in WC, and the difference in WC (in SD unit) per 1-SD difference in α- and β-diversity, adjusted for age, sex, smoking status, alcohol status, education, income, physical activity, total energy intake, Bristol stool score and time interval. WC, waist circumference; PCo, Principal coordinates; CI, confidence interval; CFI, comparative fit index; SRMR, standardized root mean square residual.

# Table S7. The temporal relationship between WC and gut microbes*

| **direction** | **ρ_1_** | **CI**  **lower** | **CI**  **upper** | ***p*** | **FDR** | **direction** | **ρ_2_** | **CI**  **lower** | **CI**  **upper** | ***p*** | **FDR** | **CFI** | **SRMR** |
| --- | --- | --- | --- | --- | --- | --- | --- | --- | --- | --- | --- | --- | --- |
| Bacteroides dorei on WC | -0.01 | -0.07 | 0.04 | 0.647 | 0.972 | WC on Bacteroides dorei | -0.01 | -0.07 | 0.06 | 0.881 | 0.948 | 1 | 2.37044E-09 |
| Bacteroides eggerthii on WC | -0.01 | -0.07 | 0.05 | 0.708 | 0.972 | WC on Bacteroides eggerthii | -0.03 | -0.1 | 0.04 | 0.409 | 0.785 | 1 | 4.19742E-09 |
| Bacteroides faecis on WC | 0.04 | -0.01 | 0.1 | 0.125 | 0.972 | WC on Bacteroides faecis | 0.01 | -0.07 | 0.1 | 0.800 | 0.912 | 1 | 2.25755E-08 |
| Bacteroides finegoldii on WC | 0.01 | -0.04 | 0.07 | 0.667 | 0.972 | WC on Bacteroides finegoldii | 0 | -0.08 | 0.08 | 0.978 | 0.986 | 1 | 1.65055E-08 |
| Bacteroides fragilis on WC | -0.01 | -0.06 | 0.05 | 0.768 | 0.972 | WC on Bacteroides fragilis | -0.02 | -0.11 | 0.06 | 0.610 | 0.894 | 1 | 3.94653E-09 |
| Bacteroides intestinalis on WC | 0.04 | -0.01 | 0.1 | 0.127 | 0.972 | WC on Bacteroides intestinalis | 0.01 | -0.08 | 0.1 | 0.892 | 0.953 | 1 | 9.58173E-10 |
| Bacteroides massiliensis on WC | 0.01 | -0.05 | 0.06 | 0.771 | 0.972 | WC on Bacteroides massiliensis | -0.01 | -0.08 | 0.06 | 0.848 | 0.932 | 1 | 1.37392E-09 |
| Bacteroides nordii on WC | -0.04 | -0.09 | 0.02 | 0.204 | 0.972 | WC on Bacteroides nordii | 0.01 | -0.08 | 0.11 | 0.771 | 0.912 | 1 | 7.92106E-09 |
| Bacteroides ovatus on WC | 0 | -0.06 | 0.06 | 0.963 | 0.972 | WC on Bacteroides ovatus | -0.06 | -0.15 | 0.04 | 0.244 | 0.632 | 1 | 7.13842E-09 |
| Bacteroides plebeius on WC | 0.08 | 0.02 | 0.13 | 0.006 | 0.571 | WC on Bacteroides plebeius | -0.01 | -0.08 | 0.07 | 0.899 | 0.955 | 1 | 3.43515E-09 |
| Bacteroides salyersiae on WC | 0.03 | -0.03 | 0.08 | 0.354 | 0.972 | WC on Bacteroides salyersiae | 0.05 | -0.03 | 0.13 | 0.227 | 0.627 | 1 | 1.20752E-09 |
| Bacteroides stercoris on WC | 0.01 | -0.05 | 0.06 | 0.815 | 0.972 | WC on Bacteroides stercoris | 0 | -0.08 | 0.08 | 0.991 | 0.991 | 1 | 8.86137E-10 |
| Bacteroides thetaiotaomicron on WC | 0 | -0.06 | 0.05 | 0.914 | 0.972 | WC on Bacteroides thetaiotaomicron | -0.08 | -0.17 | 0.02 | 0.104 | 0.481 | 1 | 2.1393E-09 |
| Bacteroides uniformis on WC | 0.02 | -0.03 | 0.08 | 0.439 | 0.972 | WC on Bacteroides uniformis | -0.04 | -0.13 | 0.06 | 0.440 | 0.785 | 1 | 3.9547E-09 |
| Bacteroides vulgatus on WC | -0.02 | -0.08 | 0.04 | 0.462 | 0.972 | WC on Bacteroides vulgatus | -0.02 | -0.1 | 0.07 | 0.705 | 0.907 | 1 | 1.19614E-08 |
| Bacteroides xylanisolvens on WC | 0.01 | -0.04 | 0.07 | 0.621 | 0.972 | WC on Bacteroides xylanisolvens | -0.01 | -0.1 | 0.08 | 0.851 | 0.932 | 1 | 9.1145E-10 |
| Bacteroidales bacterium ph8 on WC | 0.02 | -0.04 | 0.07 | 0.593 | 0.972 | WC on Bacteroidales bacterium ph8 | 0.05 | -0.03 | 0.14 | 0.192 | 0.586 | 1 | 1.35323E-08 |
| Barnesiella intestinihominis on WC | 0.01 | -0.04 | 0.07 | 0.663 | 0.972 | WC on Barnesiella intestinihominis | 0.04 | -0.04 | 0.11 | 0.330 | 0.723 | 1 | 1.80476E-09 |
| Butyricimonas synergistica on WC | 0.02 | -0.04 | 0.07 | 0.541 | 0.972 | WC on Butyricimonas synergistica | 0.04 | -0.05 | 0.13 | 0.434 | 0.785 | 1 | 1.25441E-09 |
| Coprobacter fastidiosus on WC | 0.02 | -0.04 | 0.08 | 0.496 | 0.972 | WC on Coprobacter fastidiosus | 0.01 | -0.07 | 0.1 | 0.746 | 0.912 | 1 | 6.07787E-09 |
| Odoribacter splanchnicus on WC | 0.05 | -0.01 | 0.1 | 0.102 | 0.972 | WC on Odoribacter splanchnicus | 0.1 | 0.01 | 0.18 | 0.023 | 0.260 | 1 | 1.4597E-09 |
| Parabacteroides distasonis on WC | 0.04 | -0.01 | 0.1 | 0.136 | 0.972 | WC on Parabacteroides distasonis | 0.04 | -0.04 | 0.13 | 0.323 | 0.717 | 1 | 1.04665E-08 |
| Parabacteroides goldsteinii on WC | -0.01 | -0.07 | 0.04 | 0.689 | 0.972 | WC on Parabacteroides goldsteinii | 0.04 | -0.05 | 0.13 | 0.352 | 0.753 | 1 | 1.95007E-09 |
| Parabacteroides johnsonii on WC | 0.01 | -0.05 | 0.06 | 0.847 | 0.972 | WC on Parabacteroides johnsonii | -0.02 | -0.1 | 0.06 | 0.685 | 0.907 | 1 | 5.44753E-10 |
| Parabacteroides merdae on WC | 0.04 | -0.02 | 0.1 | 0.159 | 0.972 | WC on Parabacteroides merdae | 0.07 | -0.01 | 0.15 | 0.093 | 0.453 | 1 | 1.8671E-09 |
| Actinomyces odontolyticus on WC | -0.01 | -0.06 | 0.05 | 0.851 | 0.972 | WC on Actinomyces odontolyticus | -0.02 | -0.11 | 0.07 | 0.670 | 0.907 | 1 | 4.24367E-10 |
| Parabacteroides unclassified on WC | -0.01 | -0.07 | 0.05 | 0.722 | 0.972 | WC on Parabacteroides unclassified | -0.11 | -0.2 | -0.02 | 0.012 | 0.165 | 1 | 1.08101E-09 |
| Paraprevotella clara on WC | 0.02 | -0.03 | 0.08 | 0.409 | 0.972 | WC on Paraprevotella clara | 0.04 | -0.02 | 0.1 | 0.163 | 0.586 | 1 | 7.52775E-09 |
| Paraprevotella unclassified on WC | 0.02 | -0.04 | 0.08 | 0.494 | 0.972 | WC on Paraprevotella unclassified | 0.04 | -0.02 | 0.1 | 0.180 | 0.586 | 1 | 3.94604E-09 |
| Paraprevotella xylaniphila on WC | 0.02 | -0.04 | 0.07 | 0.545 | 0.972 | WC on Paraprevotella xylaniphila | 0.02 | -0.05 | 0.08 | 0.582 | 0.872 | 1 | 1.39831E-09 |
| Prevotella copri on WC | 0.02 | -0.03 | 0.08 | 0.461 | 0.972 | WC on Prevotella copri | 0.06 | -0.02 | 0.14 | 0.140 | 0.556 | 1 | 4.84333E-09 |
| Alistipes finegoldii on WC | 0.01 | -0.05 | 0.06 | 0.783 | 0.972 | WC on Alistipes finegoldii | -0.04 | -0.11 | 0.03 | 0.282 | 0.659 | 1 | 5.90882E-09 |
| Alistipes indistinctus on WC | 0.03 | -0.03 | 0.08 | 0.328 | 0.972 | WC on Alistipes indistinctus | -0.04 | -0.13 | 0.06 | 0.436 | 0.785 | 1 | 1.24721E-08 |
| Alistipes onderdonkii on WC | 0.05 | 0 | 0.11 | 0.063 | 0.972 | WC on Alistipes onderdonkii | 0.08 | 0 | 0.15 | 0.049 | 0.322 | 1 | 1.11826E-08 |
| Alistipes putredinis on WC | 0.01 | -0.05 | 0.06 | 0.768 | 0.972 | WC on Alistipes putredinis | -0.02 | -0.1 | 0.06 | 0.602 | 0.894 | 1 | 1.61301E-09 |
| Alistipes senegalensis on WC | 0.06 | 0 | 0.11 | 0.053 | 0.972 | WC on Alistipes senegalensis | -0.04 | -0.12 | 0.05 | 0.372 | 0.776 | 1 | 3.83801E-09 |
| Alistipes shahii on WC | 0.04 | -0.01 | 0.1 | 0.151 | 0.972 | WC on Alistipes shahii | 0.01 | -0.07 | 0.1 | 0.758 | 0.912 | 1 | 5.3765E-09 |
| Alistipes sp AP11 on WC | 0 | -0.06 | 0.05 | 0.869 | 0.972 | WC on Alistipes sp AP11 | 0.03 | -0.05 | 0.11 | 0.490 | 0.808 | 1 | 3.22541E-09 |
| Alistipes unclassified on WC | 0.02 | -0.04 | 0.08 | 0.476 | 0.972 | WC on Alistipes unclassified | -0.02 | -0.11 | 0.07 | 0.676 | 0.907 | 1 | 4.08261E-09 |
| Granulicatella unclassified on WC | -0.02 | -0.07 | 0.04 | 0.523 | 0.972 | WC on Granulicatella unclassified | -0.02 | -0.11 | 0.07 | 0.672 | 0.907 | 1 | 2.381E-09 |
| Streptococcus anginosus on WC | -0.01 | -0.06 | 0.05 | 0.816 | 0.972 | WC on Streptococcus anginosus | 0.02 | -0.07 | 0.11 | 0.660 | 0.907 | 1 | 1.64151E-09 |
| Streptococcus australis on WC | -0.04 | -0.1 | 0.02 | 0.165 | 0.972 | WC on Streptococcus australis | -0.03 | -0.13 | 0.06 | 0.463 | 0.792 | 1 | 1.06158E-08 |
| Streptococcus gordonii on WC | -0.03 | -0.09 | 0.02 | 0.221 | 0.972 | WC on Streptococcus gordonii | -0.02 | -0.11 | 0.07 | 0.725 | 0.912 | 1 | 9.25117E-09 |
| Streptococcus infantis on WC | -0.01 | -0.06 | 0.05 | 0.808 | 0.972 | WC on Streptococcus infantis | -0.06 | -0.15 | 0.03 | 0.220 | 0.617 | 1 | 2.00817E-09 |
| Streptococcus mitis oralis pneumoniae on WC | -0.02 | -0.08 | 0.03 | 0.410 | 0.972 | WC on Streptococcus mitis oralis pneumoniae | 0.05 | -0.04 | 0.15 | 0.255 | 0.632 | 1 | 8.46785E-09 |
| Streptococcus parasanguinis on WC | 0 | -0.05 | 0.06 | 0.952 | 0.972 | WC on Streptococcus parasanguinis | 0 | -0.09 | 0.08 | 0.969 | 0.986 | 1 | 3.98101E-09 |
| Streptococcus salivarius on WC | -0.01 | -0.06 | 0.05 | 0.815 | 0.972 | WC on Streptococcus salivarius | -0.05 | -0.14 | 0.04 | 0.321 | 0.717 | 1 | 3.15384E-09 |
| Streptococcus sanguinis on WC | -0.02 | -0.07 | 0.04 | 0.534 | 0.972 | WC on Streptococcus sanguinis | -0.01 | -0.11 | 0.08 | 0.761 | 0.912 | 1 | 3.77072E-09 |
| Streptococcus thermophilus on WC | -0.02 | -0.08 | 0.03 | 0.463 | 0.972 | WC on Streptococcus thermophilus | -0.01 | -0.11 | 0.08 | 0.772 | 0.912 | 1 | 9.95995E-09 |
| Streptococcus vestibularis on WC | -0.04 | -0.1 | 0.01 | 0.120 | 0.972 | WC on Streptococcus vestibularis | 0.04 | -0.06 | 0.13 | 0.455 | 0.785 | 1 | 3.13023E-09 |
| Clostridiaceae bacterium JC118 on WC | 0.01 | -0.04 | 0.07 | 0.622 | 0.972 | WC on Clostridiaceae bacterium JC118 | -0.02 | -0.11 | 0.07 | 0.626 | 0.894 | 1 | 1.53096E-09 |
| Clostridium asparagiforme on WC | 0.04 | -0.01 | 0.1 | 0.123 | 0.972 | WC on Clostridium asparagiforme | -0.07 | -0.16 | 0.02 | 0.151 | 0.575 | 1 | 1.50586E-09 |
| Clostridium bolteae on WC | -0.03 | -0.08 | 0.03 | 0.347 | 0.972 | WC on Clostridium bolteae | -0.07 | -0.16 | 0.02 | 0.107 | 0.481 | 1 | 6.79622E-09 |
| Clostridium citroniae on WC | -0.01 | -0.07 | 0.05 | 0.742 | 0.972 | WC on Clostridium citroniae | -0.04 | -0.13 | 0.05 | 0.430 | 0.785 | 1 | 1.9726E-09 |
| Clostridium clostridioforme on WC | 0.01 | -0.05 | 0.07 | 0.731 | 0.972 | WC on Clostridium clostridioforme | -0.01 | -0.1 | 0.08 | 0.808 | 0.915 | 1 | 2.42118E-09 |
| Clostridium hathewayi on WC | 0.01 | -0.05 | 0.06 | 0.861 | 0.972 | WC on Clostridium hathewayi | -0.12 | -0.22 | -0.03 | 0.009 | 0.146 | 1 | 8.01326E-09 |
| Clostridium leptum on WC | 0.04 | -0.01 | 0.1 | 0.116 | 0.972 | WC on Clostridium leptum | 0.03 | -0.06 | 0.12 | 0.491 | 0.808 | 1 | 3.60628E-09 |
| Clostridium nexile on WC | -0.05 | -0.1 | 0.01 | 0.092 | 0.972 | WC on Clostridium nexile | 0.03 | -0.06 | 0.12 | 0.541 | 0.855 | 1 | 2.66189E-09 |
| Clostridium sp ATCC BAA 442 on WC | 0.01 | -0.05 | 0.06 | 0.776 | 0.972 | WC on Clostridium sp ATCC BAA 442 | -0.07 | -0.17 | 0.02 | 0.148 | 0.574 | 1 | 6.4645E-10 |
| Clostridium symbiosum on WC | -0.03 | -0.08 | 0.03 | 0.369 | 0.972 | WC on Clostridium symbiosum | -0.06 | -0.15 | 0.03 | 0.192 | 0.586 | 1 | 4.74028E-09 |
| Clostridiales bacterium 1 7 47FAA on WC | -0.03 | -0.09 | 0.02 | 0.249 | 0.972 | WC on Clostridiales bacterium 1 7 47FAA | -0.06 | -0.15 | 0.03 | 0.218 | 0.617 | 1 | 2.04897E-09 |
| Flavonifractor plautii on WC | -0.02 | -0.07 | 0.04 | 0.544 | 0.972 | WC on Flavonifractor plautii | -0.06 | -0.15 | 0.03 | 0.214 | 0.617 | 1 | 4.49815E-10 |
| Eubacterium eligens on WC | 0.05 | 0 | 0.11 | 0.070 | 0.972 | WC on Eubacterium eligens | 0.01 | -0.08 | 0.11 | 0.782 | 0.912 | 1 | 2.44863E-09 |
| Eubacterium hallii on WC | 0.05 | -0.01 | 0.1 | 0.100 | 0.972 | WC on Eubacterium hallii | 0.06 | -0.03 | 0.16 | 0.178 | 0.586 | 1 | 2.47044E-09 |
| Eubacterium limosum on WC | -0.03 | -0.08 | 0.03 | 0.331 | 0.972 | WC on Eubacterium limosum | 0.04 | -0.06 | 0.13 | 0.431 | 0.785 | 1 | 7.943E-10 |
| Eubacterium ramulus on WC | -0.01 | -0.07 | 0.04 | 0.643 | 0.972 | WC on Eubacterium ramulus | 0.09 | 0 | 0.17 | 0.039 | 0.302 | 1 | 4.85571E-09 |
| Eubacterium rectale on WC | 0.03 | -0.02 | 0.09 | 0.237 | 0.972 | WC on Eubacterium rectale | -0.02 | -0.11 | 0.07 | 0.695 | 0.907 | 1 | 8.34463E-09 |
| Eubacterium siraeum on WC | 0 | -0.06 | 0.05 | 0.911 | 0.972 | WC on Eubacterium siraeum | 0.01 | -0.08 | 0.1 | 0.814 | 0.915 | 1 | 1.60452E-09 |
| Eubacterium ventriosum on WC | 0 | -0.05 | 0.06 | 0.890 | 0.972 | WC on Eubacterium ventriosum | 0.06 | -0.03 | 0.16 | 0.207 | 0.610 | 1 | 1.15139E-08 |
| Anaerostipes hadrus on WC | 0 | -0.06 | 0.05 | 0.928 | 0.972 | WC on Anaerostipes hadrus | 0.03 | -0.07 | 0.12 | 0.578 | 0.872 | 1 | 6.92637E-09 |
| Anaerostipes unclassified on WC | 0.02 | -0.04 | 0.07 | 0.561 | 0.972 | WC on Anaerostipes unclassified | -0.05 | -0.15 | 0.04 | 0.268 | 0.636 | 1 | 6.71167E-09 |
| Blautia producta on WC | -0.01 | -0.07 | 0.04 | 0.641 | 0.972 | WC on Blautia producta | -0.08 | -0.17 | 0.01 | 0.091 | 0.453 | 1 | 2.03619E-09 |
| Ruminococcus gnavus on WC | -0.05 | -0.1 | 0.01 | 0.100 | 0.972 | WC on Ruminococcus gnavus | -0.03 | -0.11 | 0.06 | 0.560 | 0.862 | 1 | 1.83388E-10 |
| Ruminococcus obeum on WC | 0.03 | -0.03 | 0.08 | 0.337 | 0.972 | WC on Ruminococcus obeum | 0.03 | -0.05 | 0.12 | 0.442 | 0.785 | 1 | 5.35171E-10 |
| Ruminococcus torques on WC | -0.01 | -0.07 | 0.04 | 0.677 | 0.972 | WC on Ruminococcus torques | -0.01 | -0.11 | 0.08 | 0.781 | 0.912 | 1 | 5.25028E-09 |
| Coprococcus catus on WC | -0.01 | -0.07 | 0.04 | 0.680 | 0.972 | WC on Coprococcus catus | 0.03 | -0.06 | 0.11 | 0.549 | 0.855 | 1 | 1.89521E-09 |
| Coprococcus comes on WC | 0.03 | -0.02 | 0.09 | 0.231 | 0.972 | WC on Coprococcus comes | 0.07 | -0.01 | 0.16 | 0.085 | 0.453 | 1 | 9.50976E-09 |
| Coprococcus sp ART55 1 on WC | 0 | -0.06 | 0.05 | 0.874 | 0.972 | WC on Coprococcus sp ART55 1 | -0.13 | -0.22 | -0.04 | 0.004 | 0.120 | 1 | 3.76464E-09 |
| Dorea formicigenerans on WC | 0.02 | -0.03 | 0.08 | 0.387 | 0.972 | WC on Dorea formicigenerans | 0.06 | -0.03 | 0.15 | 0.178 | 0.586 | 1 | 5.95263E-09 |
| Dorea longicatena on WC | -0.01 | -0.06 | 0.05 | 0.794 | 0.972 | WC on Dorea longicatena | 0.03 | -0.06 | 0.13 | 0.450 | 0.785 | 1 | 3.34464E-09 |
| Dorea unclassified on WC | 0 | -0.05 | 0.06 | 0.925 | 0.972 | WC on Dorea unclassified | -0.04 | -0.14 | 0.05 | 0.366 | 0.773 | 1 | 2.52226E-09 |
| Lachnospiraceae bacterium 1 1 57FAA on WC | 0.03 | -0.03 | 0.08 | 0.317 | 0.972 | WC on Lachnospiraceae bacterium 1 1 57FAA | 0.07 | -0.02 | 0.16 | 0.130 | 0.541 | 1 | 1.1695E-09 |
| Lachnospiraceae bacterium 1 4 56FAA on WC | -0.02 | -0.08 | 0.04 | 0.496 | 0.972 | WC on Lachnospiraceae bacterium 1 4 56FAA | 0.08 | -0.01 | 0.17 | 0.068 | 0.386 | 1 | 1.13797E-09 |
| Lachnospiraceae bacterium 2 1 58FAA on WC | -0.02 | -0.08 | 0.03 | 0.436 | 0.972 | WC on Lachnospiraceae bacterium 2 1 58FAA | 0.09 | -0.01 | 0.18 | 0.069 | 0.386 | 1 | 3.58219E-09 |
| Lachnospiraceae bacterium 3 1 46FAA on WC | -0.02 | -0.07 | 0.04 | 0.540 | 0.972 | WC on Lachnospiraceae bacterium 3 1 46FAA | 0.05 | -0.04 | 0.14 | 0.263 | 0.634 | 1 | 3.76043E-09 |
| Lachnospiraceae bacterium 3 1 57FAA CT1 on WC | -0.04 | -0.1 | 0.01 | 0.132 | 0.972 | WC on Lachnospiraceae bacterium 3 1 57FAA CT1 | -0.1 | -0.19 | -0.01 | 0.032 | 0.299 | 1 | 5.95225E-09 |
| Lachnospiraceae bacterium 5 1 57FAA on WC | 0.02 | -0.03 | 0.08 | 0.404 | 0.972 | WC on Lachnospiraceae bacterium 5 1 57FAA | 0.02 | -0.07 | 0.11 | 0.704 | 0.907 | 1 | 1.05467E-09 |
| Lachnospiraceae bacterium 5 1 63FAA on WC | 0.03 | -0.03 | 0.08 | 0.359 | 0.972 | WC on Lachnospiraceae bacterium 5 1 63FAA | 0.02 | -0.07 | 0.11 | 0.640 | 0.894 | 1 | 6.74431E-09 |
| Lachnospiraceae bacterium 7 1 58FAA on WC | 0.01 | -0.05 | 0.06 | 0.760 | 0.972 | WC on Lachnospiraceae bacterium 7 1 58FAA | -0.09 | -0.18 | 0 | 0.057 | 0.359 | 1 | 9.13035E-09 |
| Lachnospiraceae bacterium 8 1 57FAA on WC | -0.03 | -0.08 | 0.03 | 0.370 | 0.972 | WC on Lachnospiraceae bacterium 8 1 57FAA | -0.03 | -0.13 | 0.06 | 0.500 | 0.814 | 1 | 1.94963E-09 |
| Lachnospiraceae bacterium 9 1 43BFAA on WC | -0.03 | -0.09 | 0.02 | 0.251 | 0.972 | WC on Lachnospiraceae bacterium 9 1 43BFAA | 0 | -0.08 | 0.09 | 0.942 | 0.986 | 1 | 4.10492E-09 |
| Roseburia hominis on WC | 0 | -0.05 | 0.06 | 0.867 | 0.972 | WC on Roseburia hominis | 0.07 | -0.02 | 0.16 | 0.135 | 0.550 | 1 | 6.41673E-09 |
| Roseburia intestinalis on WC | 0.03 | -0.03 | 0.09 | 0.303 | 0.972 | WC on Roseburia intestinalis | -0.01 | -0.1 | 0.09 | 0.875 | 0.947 | 1 | 8.78458E-10 |
| Roseburia inulinivorans on WC | -0.02 | -0.08 | 0.03 | 0.389 | 0.972 | WC on Roseburia inulinivorans | 0.07 | -0.02 | 0.16 | 0.127 | 0.541 | 1 | 1.89504E-09 |
| Roseburia unclassified on WC | 0.02 | -0.04 | 0.08 | 0.473 | 0.972 | WC on Roseburia unclassified | -0.05 | -0.15 | 0.04 | 0.259 | 0.632 | 1 | 1.98486E-09 |
| Oscillibacter unclassified on WC | -0.03 | -0.08 | 0.03 | 0.328 | 0.972 | WC on Oscillibacter unclassified | 0.04 | -0.05 | 0.13 | 0.400 | 0.785 | 1 | 1.03241E-08 |
| Clostridium bartlettii on WC | 0.04 | -0.01 | 0.1 | 0.140 | 0.972 | WC on Clostridium bartlettii | -0.02 | -0.12 | 0.07 | 0.612 | 0.894 | 1 | 2.54585E-09 |
| Peptostreptococcaceae noname unclassified on WC | 0.06 | 0 | 0.11 | 0.038 | 0.972 | WC on Peptostreptococcaceae noname unclassified | 0.01 | -0.08 | 0.11 | 0.834 | 0.930 | 1 | 4.90752E-09 |
| Anaerotruncus colihominis on WC | -0.01 | -0.07 | 0.05 | 0.739 | 0.972 | WC on Anaerotruncus colihominis | -0.01 | -0.1 | 0.08 | 0.796 | 0.912 | 1 | 1.1362E-09 |
| Anaerotruncus unclassified on WC | 0 | -0.05 | 0.06 | 0.951 | 0.972 | WC on Anaerotruncus unclassified | -0.05 | -0.15 | 0.04 | 0.259 | 0.632 | 1 | 4.64864E-09 |
| Faecalibacterium prausnitzii on WC | 0.02 | -0.04 | 0.08 | 0.509 | 0.972 | WC on Faecalibacterium prausnitzii | 0.01 | -0.08 | 0.1 | 0.868 | 0.945 | 1 | 2.53078E-09 |
| Ruminococcaceae bacterium D16 on WC | -0.01 | -0.07 | 0.05 | 0.755 | 0.972 | WC on Ruminococcaceae bacterium D16 | 0.01 | -0.07 | 0.1 | 0.757 | 0.912 | 1 | 2.18811E-09 |
| Ruminococcus bromii on WC | 0.06 | 0 | 0.11 | 0.048 | 0.972 | WC on Ruminococcus bromii | -0.05 | -0.14 | 0.04 | 0.246 | 0.632 | 1 | 9.07224E-09 |
| Ruminococcus callidus on WC | 0.02 | -0.04 | 0.07 | 0.566 | 0.972 | WC on Ruminococcus callidus | 0.06 | -0.03 | 0.15 | 0.182 | 0.586 | 1 | 3.41882E-09 |
| Ruminococcus lactaris on WC | 0.01 | -0.05 | 0.06 | 0.777 | 0.972 | WC on Ruminococcus lactaris | -0.07 | -0.15 | 0.02 | 0.125 | 0.541 | 1 | 1.07788E-08 |
| Ruminococcus sp 5 1 39BFAA on WC | 0.04 | -0.02 | 0.09 | 0.183 | 0.972 | WC on Ruminococcus sp 5 1 39BFAA | 0.12 | 0.03 | 0.21 | 0.013 | 0.165 | 1 | 2.44244E-09 |
| Subdoligranulum sp 4 3 54A2FAA on WC | 0.02 | -0.04 | 0.07 | 0.578 | 0.972 | WC on Subdoligranulum sp 4 3 54A2FAA | -0.03 | -0.12 | 0.06 | 0.550 | 0.855 | 1 | 2.1024E-09 |
| Subdoligranulum unclassified on WC | 0.02 | -0.03 | 0.08 | 0.460 | 0.972 | WC on Subdoligranulum unclassified | -0.03 | -0.12 | 0.06 | 0.526 | 0.840 | 1 | 6.84241E-09 |
| Coprobacillus unclassified on WC | -0.01 | -0.07 | 0.04 | 0.638 | 0.972 | WC on Coprobacillus unclassified | 0 | -0.09 | 0.09 | 0.978 | 0.986 | 1 | 7.51909E-10 |
| Clostridium ramosum on WC | 0.01 | -0.05 | 0.06 | 0.852 | 0.972 | WC on Clostridium ramosum | 0.03 | -0.06 | 0.12 | 0.516 | 0.832 | 1 | 8.83357E-09 |
| Eubacterium biforme on WC | 0.01 | -0.04 | 0.07 | 0.668 | 0.972 | WC on Eubacterium biforme | 0.04 | -0.04 | 0.11 | 0.340 | 0.735 | 1 | 1.20675E-08 |
| Holdemania filiformis on WC | 0.02 | -0.03 | 0.08 | 0.392 | 0.972 | WC on Holdemania filiformis | 0.13 | 0.04 | 0.22 | 0.004 | 0.120 | 1 | 7.06583E-09 |
| Holdemania unclassified on WC | 0.01 | -0.04 | 0.07 | 0.677 | 0.972 | WC on Holdemania unclassified | -0.01 | -0.1 | 0.08 | 0.799 | 0.912 | 1 | 3.07345E-09 |
| Acidaminococcus intestini on WC | 0 | -0.06 | 0.06 | 0.967 | 0.972 | WC on Acidaminococcus intestini | 0.12 | 0.03 | 0.21 | 0.007 | 0.146 | 1 | 3.64276E-09 |
| Acidaminococcus unclassified on WC | -0.01 | -0.07 | 0.05 | 0.726 | 0.972 | WC on Acidaminococcus unclassified | 0.06 | -0.03 | 0.14 | 0.200 | 0.599 | 1 | 1.6149E-08 |
| Dialister invisus on WC | 0 | -0.06 | 0.05 | 0.917 | 0.972 | WC on Dialister invisus | -0.06 | -0.15 | 0.04 | 0.239 | 0.632 | 1 | 7.99783E-10 |
| Megamonas funiformis on WC | 0.03 | -0.03 | 0.08 | 0.362 | 0.972 | WC on Megamonas funiformis | 0.09 | 0 | 0.17 | 0.045 | 0.312 | 1 | 6.6582E-10 |
| Megamonas hypermegale on WC | 0 | -0.05 | 0.06 | 0.955 | 0.972 | WC on Megamonas hypermegale | 0.13 | 0.04 | 0.21 | 0.003 | 0.120 | 1 | 1.96238E-09 |
| Megamonas rupellensis on WC | 0 | -0.06 | 0.05 | 0.952 | 0.972 | WC on Megamonas rupellensis | 0.08 | 0 | 0.17 | 0.062 | 0.380 | 1 | 8.95481E-09 |
| Megamonas unclassified on WC | 0.01 | -0.05 | 0.07 | 0.743 | 0.972 | WC on Megamonas unclassified | 0.12 | 0.04 | 0.21 | 0.004 | 0.120 | 1 | 3.90813E-09 |
| Megasphaera micronuciformis on WC | -0.02 | -0.08 | 0.03 | 0.459 | 0.972 | WC on Megasphaera micronuciformis | -0.07 | -0.16 | 0.03 | 0.159 | 0.586 | 1 | 7.09597E-09 |
| Megasphaera unclassified on WC | -0.02 | -0.07 | 0.04 | 0.540 | 0.972 | WC on Megasphaera unclassified | 0.15 | 0.07 | 0.24 | 0.000 | 0.038 | 1 | 2.53321E-09 |
| Veillonella atypica on WC | -0.02 | -0.07 | 0.04 | 0.515 | 0.972 | WC on Veillonella atypica | -0.06 | -0.15 | 0.03 | 0.184 | 0.586 | 1 | 2.33175E-09 |
| Veillonella dispar on WC | -0.02 | -0.08 | 0.03 | 0.458 | 0.972 | WC on Veillonella dispar | -0.03 | -0.12 | 0.05 | 0.436 | 0.785 | 1 | 3.7105E-09 |
| Veillonella parvula on WC | -0.01 | -0.07 | 0.05 | 0.714 | 0.972 | WC on Veillonella parvula | -0.09 | -0.18 | 0 | 0.038 | 0.302 | 1 | 2.70361E-09 |
| Veillonella unclassified on WC | -0.01 | -0.06 | 0.05 | 0.818 | 0.972 | WC on Veillonella unclassified | -0.11 | -0.2 | -0.03 | 0.009 | 0.146 | 1 | 3.00635E-09 |
| Fusobacterium mortiferum on WC | -0.04 | -0.09 | 0.02 | 0.209 | 0.972 | WC on Fusobacterium mortiferum | 0.04 | -0.05 | 0.13 | 0.422 | 0.785 | 1 | 1.00032E-08 |
| Fusobacterium ulcerans on WC | 0.03 | -0.03 | 0.08 | 0.362 | 0.972 | WC on Fusobacterium ulcerans | -0.06 | -0.15 | 0.04 | 0.251 | 0.632 | 1 | 3.25516E-09 |
| Fusobacterium varium on WC | -0.02 | -0.08 | 0.03 | 0.444 | 0.972 | WC on Fusobacterium varium | -0.09 | -0.19 | 0 | 0.046 | 0.312 | 1 | 3.77647E-09 |
| Rothia mucilaginosa on WC | -0.02 | -0.08 | 0.03 | 0.442 | 0.972 | WC on Rothia mucilaginosa | -0.03 | -0.12 | 0.06 | 0.478 | 0.801 | 1 | 3.15971E-09 |
| Burkholderiales bacterium 1 1 47 on WC | -0.03 | -0.08 | 0.03 | 0.319 | 0.972 | WC on Burkholderiales bacterium 1 1 47 | 0.1 | 0.01 | 0.19 | 0.036 | 0.302 | 1 | 3.36941E-09 |
| Oxalobacter formigenes on WC | 0.02 | -0.04 | 0.08 | 0.496 | 0.972 | WC on Oxalobacter formigenes | 0 | -0.09 | 0.09 | 0.980 | 0.986 | 1 | 6.74734E-09 |
| Parasutterella excrementihominis on WC | -0.01 | -0.06 | 0.05 | 0.800 | 0.972 | WC on Parasutterella excrementihominis | 0.08 | -0.01 | 0.17 | 0.092 | 0.453 | 1 | 9.32833E-09 |
| Sutterella wadsworthensis on WC | 0.02 | -0.04 | 0.07 | 0.528 | 0.972 | WC on Sutterella wadsworthensis | 0.03 | -0.05 | 0.11 | 0.477 | 0.801 | 1 | 8.23435E-09 |
| Bilophila unclassified on WC | 0 | -0.05 | 0.06 | 0.934 | 0.972 | WC on Bilophila unclassified | 0.1 | 0 | 0.19 | 0.046 | 0.312 | 1 | 1.82228E-09 |
| Bilophila wadsworthia on WC | 0 | -0.05 | 0.06 | 0.904 | 0.972 | WC on Bilophila wadsworthia | 0.01 | -0.08 | 0.1 | 0.792 | 0.912 | 1 | 1.36894E-09 |
| Desulfovibrio desulfuricans on WC | -0.01 | -0.07 | 0.05 | 0.739 | 0.972 | WC on Desulfovibrio desulfuricans | 0.04 | -0.05 | 0.13 | 0.419 | 0.785 | 1 | 4.36459E-09 |
| Desulfovibrio piger on WC | 0.03 | -0.02 | 0.09 | 0.252 | 0.972 | WC on Desulfovibrio piger | 0 | -0.08 | 0.08 | 0.928 | 0.980 | 1 | 4.6199E-09 |
| Bifidobacterium adolescentis on WC | 0 | -0.05 | 0.06 | 0.953 | 0.972 | WC on Bifidobacterium adolescentis | 0.02 | -0.07 | 0.11 | 0.695 | 0.907 | 1 | 6.11987E-09 |
| Citrobacter freundii on WC | 0.01 | -0.05 | 0.07 | 0.755 | 0.972 | WC on Citrobacter freundii | -0.04 | -0.14 | 0.05 | 0.385 | 0.785 | 1 | 3.3188E-09 |
| Citrobacter unclassified on WC | 0.02 | -0.03 | 0.08 | 0.406 | 0.972 | WC on Citrobacter unclassified | -0.02 | -0.12 | 0.07 | 0.626 | 0.894 | 1 | 4.43044E-09 |
| Enterobacter aerogenes on WC | 0 | -0.06 | 0.05 | 0.960 | 0.972 | WC on Enterobacter aerogenes | 0.02 | -0.07 | 0.12 | 0.635 | 0.894 | 1 | 3.28978E-09 |
| Enterobacter cloacae on WC | 0.06 | 0 | 0.11 | 0.050 | 0.972 | WC on Enterobacter cloacae | -0.04 | -0.13 | 0.06 | 0.447 | 0.785 | 1 | 2.245E-09 |
| Escherichia coli on WC | 0.05 | -0.01 | 0.1 | 0.110 | 0.972 | WC on Escherichia coli | -0.02 | -0.11 | 0.07 | 0.643 | 0.894 | 1 | 2.50758E-09 |
| Escherichia unclassified on WC | 0.04 | -0.02 | 0.1 | 0.158 | 0.972 | WC on Escherichia unclassified | 0 | -0.09 | 0.09 | 0.967 | 0.986 | 1 | 6.42554E-09 |
| Bifidobacterium bifidum on WC | 0.03 | -0.03 | 0.09 | 0.310 | 0.972 | WC on Bifidobacterium bifidum | 0.08 | -0.01 | 0.17 | 0.099 | 0.469 | 1 | 2.85453E-09 |
| Klebsiella pneumoniae on WC | 0.04 | -0.01 | 0.1 | 0.117 | 0.972 | WC on Klebsiella pneumoniae | -0.04 | -0.13 | 0.05 | 0.399 | 0.785 | 1 | 8.10935E-09 |
| Klebsiella unclassified on WC | 0.03 | -0.02 | 0.09 | 0.267 | 0.972 | WC on Klebsiella unclassified | -0.05 | -0.14 | 0.05 | 0.322 | 0.717 | 1 | 1.50956E-09 |
| Bifidobacterium dentium on WC | 0 | -0.06 | 0.05 | 0.869 | 0.972 | WC on Bifidobacterium dentium | -0.01 | -0.1 | 0.07 | 0.738 | 0.912 | 1 | 4.04012E-09 |
| Haemophilus parainfluenzae on WC | -0.01 | -0.07 | 0.04 | 0.617 | 0.972 | WC on Haemophilus parainfluenzae | -0.06 | -0.15 | 0.03 | 0.177 | 0.586 | 1 | 3.15719E-09 |
| Bifidobacterium longum on WC | 0 | -0.06 | 0.06 | 0.981 | 0.981 | WC on Bifidobacterium longum | 0.05 | -0.04 | 0.14 | 0.302 | 0.698 | 1 | 8.43179E-10 |
| Bifidobacterium pseudocatenulatum on WC | -0.03 | -0.09 | 0.02 | 0.234 | 0.972 | WC on Bifidobacterium pseudocatenulatum | 0.12 | 0.03 | 0.21 | 0.009 | 0.146 | 1 | 9.13783E-10 |
| Pyramidobacter piscolens on WC | 0.01 | -0.04 | 0.07 | 0.610 | 0.972 | WC on Pyramidobacter piscolens | 0.02 | -0.08 | 0.11 | 0.717 | 0.912 | 1 | 7.06069E-09 |
| Akkermansia muciniphila on WC | 0.01 | -0.04 | 0.07 | 0.667 | 0.972 | WC on Akkermansia muciniphila | 0.05 | -0.04 | 0.15 | 0.242 | 0.632 | 1 | 5.20031E-09 |
| Scardovia wiggsiae on WC | 0.01 | -0.05 | 0.06 | 0.857 | 0.972 | WC on Scardovia wiggsiae | -0.1 | -0.19 | -0.01 | 0.038 | 0.302 | 1 | 3.49908E-09 |
| Adlercreutzia equolifaciens on WC | 0.01 | -0.05 | 0.06 | 0.829 | 0.972 | WC on Adlercreutzia equolifaciens | 0.16 | 0.07 | 0.24 | 0.000 | 0.038 | 1 | 1.00176E-08 |
| Murine osteosarcoma virus on WC | 0 | -0.06 | 0.05 | 0.907 | 0.972 | WC on Murine osteosarcoma virus | -0.03 | -0.12 | 0.07 | 0.581 | 0.872 | 1 | 1.28066E-08 |
| Atopobium parvulum on WC | -0.04 | -0.09 | 0.02 | 0.201 | 0.972 | WC on Atopobium parvulum | 0.01 | -0.08 | 0.1 | 0.838 | 0.930 | 1 | 5.0064E-09 |
| Collinsella aerofaciens on WC | -0.04 | -0.09 | 0.02 | 0.178 | 0.972 | WC on Collinsella aerofaciens | 0.1 | 0.01 | 0.18 | 0.026 | 0.279 | 1 | 7.71273E-09 |
| Collinsella intestinalis on WC | -0.03 | -0.09 | 0.02 | 0.275 | 0.972 | WC on Collinsella intestinalis | 0.02 | -0.07 | 0.1 | 0.730 | 0.912 | 1 | 1.36339E-08 |
| Collinsella tanakaei on WC | -0.05 | -0.1 | 0.01 | 0.095 | 0.972 | WC on Collinsella tanakaei | 0.11 | 0.03 | 0.2 | 0.007 | 0.146 | 1 | 9.16152E-10 |
| Coriobacteriaceae bacterium phI on WC | -0.04 | -0.1 | 0.01 | 0.147 | 0.972 | WC on Coriobacteriaceae bacterium phI | -0.04 | -0.12 | 0.05 | 0.416 | 0.785 | 1 | 1.04508E-08 |
| Eggerthella lenta on WC | -0.01 | -0.07 | 0.04 | 0.677 | 0.972 | WC on Eggerthella lenta | 0 | -0.09 | 0.09 | 0.959 | 0.986 | 1 | 8.09637E-10 |
| Eggerthella unclassified on WC | 0.01 | -0.05 | 0.06 | 0.754 | 0.972 | WC on Eggerthella unclassified | 0 | -0.09 | 0.09 | 0.970 | 0.986 | 1 | 9.92763E-09 |
| Gordonibacter pamelaeae on WC | 0 | -0.05 | 0.06 | 0.863 | 0.972 | WC on Gordonibacter pamelaeae | 0.1 | 0.01 | 0.18 | 0.030 | 0.297 | 1 | 6.83907E-10 |
| Slackia piriformis on WC | 0.01 | -0.05 | 0.07 | 0.722 | 0.972 | WC on Slackia piriformis | 0.02 | -0.06 | 0.1 | 0.701 | 0.907 | 1 | 2.66952E-09 |
| Bacteroides caccae on WC | 0 | -0.06 | 0.05 | 0.959 | 0.972 | WC on Bacteroides caccae | 0.1 | 0.01 | 0.18 | 0.023 | 0.260 | 1 | 6.11053E-09 |
| Bacteroides cellulosilyticus on WC | 0.01 | -0.04 | 0.07 | 0.652 | 0.972 | WC on Bacteroides cellulosilyticus | -0.02 | -0.12 | 0.07 | 0.636 | 0.894 | 1 | 2.52152E-09 |
| Bacteroides clarus on WC | -0.06 | -0.12 | 0 | 0.034 | 0.972 | WC on Bacteroides clarus | -0.01 | -0.09 | 0.07 | 0.792 | 0.912 | 1 | 4.91469E-09 |
| Bacteroides coprocola on WC | 0.02 | -0.04 | 0.08 | 0.493 | 0.972 | WC on Bacteroides coprocola | -0.05 | -0.13 | 0.03 | 0.188 | 0.586 | 1 | 5.28317E-09 |
| Bacteroides coprophilus on WC | 0.08 | 0.02 | 0.13 | 0.007 | 0.571 | WC on Bacteroides coprophilus | -0.06 | -0.13 | 0.01 | 0.070 | 0.386 | 1 | 1.60832E-09 |

*The cross-lagged path analysis was used to estimate the difference in the abundance of gut microbes (in SD unit of the log-transformed abundance) per 1-SD difference in WC, and the difference in WC (in SD unit) per 1-SD difference in the log-transformed abundance of gut microbes, adjusted for age, sex, smoking status, alcohol status, education, income, physical activity, total energy intake, Bristol stool score and time interval. WC, waist circumference; CI, confidence interval; FDR. false discovery rate; CFI, comparative fit index; SRMR, standardized root mean square residual.

# Table S8. The prospective associations between dietary factors and identified microbes*

| Diet | Taxonomy | Estimate | CI lower | CI upper | SE | *t* value | *p* | FDR |
| --- | --- | --- | --- | --- | --- | --- | --- | --- |
| Fish intake | *Ruminococcus sp 5 1 39BFAA* | 0.36 | 0.16 | 0.55 | 0.1 | 3.58 | <0.001 | 0.019 |
| Red and processed meat intake | *Lachnospiraceae bacterium 3 1 57FAA CT1* | 0.22 | 0.03 | 0.42 | 0.1 | 2.22 | 0.027 | 0.338 |
| Vegetable intake | *Ruminococcus sp 5 1 39BFAA* | 0.22 | 0.03 | 0.42 | 0.1 | 2.23 | 0.026 | 0.338 |
| Fish intake | *Megamonas hypermegale* | 0.21 | 0.03 | 0.39 | 0.09 | 2.32 | 0.021 | 0.338 |
| Fruit intake | *Lachnospiraceae bacterium 3 1 57FAA CT1* | -0.2 | -0.39 | -0.01 | 0.1 | -2.02 | 0.044 | 0.437 |
| Fruit intake | *Adlercreutzia equolifaciens* | -0.18 | -0.36 | 0 | 0.09 | -1.94 | 0.053 | 0.444 |
| Fish intake | *Clostridium hathewayi* | -0.18 | -0.38 | 0.01 | 0.1 | -1.85 | 0.065 | 0.462 |
| Vegetable intake | *Parabacteroides unclassified* | -0.16 | -0.34 | 0.03 | 0.09 | -1.69 | 0.092 | 0.575 |
| Dairy intake | *Ruminococcus sp 5 1 39BFAA* | 0.13 | -0.06 | 0.33 | 0.1 | 1.34 | 0.179 | 0.719 |
| Fish intake | *Megamonas unclassified* | 0.12 | -0.06 | 0.29 | 0.09 | 1.29 | 0.197 | 0.719 |
| Vegetable intake | *Bacteroides caccae* | 0.12 | -0.05 | 0.29 | 0.09 | 1.35 | 0.178 | 0.719 |
| Fish intake | *Bacteroides caccae* | 0.13 | -0.04 | 0.31 | 0.09 | 1.5 | 0.134 | 0.719 |
| Red and processed meat intake | *Bacteroides caccae* | -0.12 | -0.3 | 0.06 | 0.09 | -1.28 | 0.201 | 0.719 |
| Dairy intake | *Bacteroides caccae* | -0.12 | -0.29 | 0.06 | 0.09 | -1.31 | 0.19 | 0.719 |
| Red and processed meat intake | *Parabacteroides unclassified* | 0.09 | -0.1 | 0.28 | 0.1 | 0.92 | 0.356 | 0.858 |
| Vegetable intake | *Clostridium hathewayi* | -0.09 | -0.29 | 0.1 | 0.1 | -0.94 | 0.346 | 0.858 |
| Red and processed meat intake | *Clostridium hathewayi* | -0.09 | -0.29 | 0.12 | 0.1 | -0.84 | 0.4 | 0.858 |
| Fish intake | *Lachnospiraceae bacterium 3 1 57FAA CT1* | -0.08 | -0.27 | 0.11 | 0.1 | -0.83 | 0.404 | 0.858 |
| Dairy intake | *Lachnospiraceae bacterium 3 1 57FAA CT1* | -0.07 | -0.26 | 0.12 | 0.1 | -0.72 | 0.473 | 0.858 |
| Fruit intake | *Lachnospiraceae bacterium 7 1 58FAA* | -0.07 | -0.26 | 0.12 | 0.1 | -0.75 | 0.453 | 0.858 |
| Fish intake | *Lachnospiraceae bacterium 7 1 58FAA* | -0.09 | -0.28 | 0.1 | 0.1 | -0.94 | 0.346 | 0.858 |
| Red and processed meat intake | *Lachnospiraceae bacterium 7 1 58FAA* | 0.1 | -0.09 | 0.3 | 0.1 | 1.04 | 0.299 | 0.858 |
| Fruit intake | *Ruminococcus sp 5 1 39BFAA* | 0.08 | -0.12 | 0.28 | 0.1 | 0.83 | 0.408 | 0.858 |
| Red and processed meat intake | *Ruminococcus sp 5 1 39BFAA* | 0.08 | -0.12 | 0.29 | 0.1 | 0.81 | 0.417 | 0.858 |
| Dairy intake | *Megamonas unclassified* | 0.06 | -0.12 | 0.23 | 0.09 | 0.65 | 0.514 | 0.858 |
| Fruit intake | *Megasphaera unclassified* | -0.08 | -0.26 | 0.11 | 0.1 | -0.78 | 0.433 | 0.858 |
| Fish intake | *Megasphaera unclassified* | -0.08 | -0.26 | 0.11 | 0.09 | -0.84 | 0.403 | 0.858 |
| Dairy intake | *Megasphaera unclassified* | -0.06 | -0.24 | 0.12 | 0.09 | -0.63 | 0.532 | 0.858 |
| Fish intake | *Adlercreutzia equolifaciens* | 0.09 | -0.09 | 0.27 | 0.09 | 0.95 | 0.341 | 0.858 |
| Dairy intake | *Adlercreutzia equolifaciens* | 0.06 | -0.11 | 0.24 | 0.09 | 0.71 | 0.481 | 0.858 |
| Fruit intake | *Bacteroides caccae* | -0.06 | -0.23 | 0.12 | 0.09 | -0.63 | 0.532 | 0.858 |
| Red and processed meat intake | *Adlercreutzia equolifaciens* | 0.06 | -0.13 | 0.24 | 0.1 | 0.59 | 0.558 | 0.873 |
| Fruit intake | *Megamonas hypermegale* | -0.04 | -0.23 | 0.14 | 0.09 | -0.48 | 0.633 | 0.883 |
| Red and processed meat intake | *Megamonas hypermegale* | -0.04 | -0.23 | 0.14 | 0.09 | -0.44 | 0.66 | 0.883 |
| Dairy intake | *Megamonas hypermegale* | 0.04 | -0.13 | 0.22 | 0.09 | 0.48 | 0.635 | 0.883 |
| Vegetable intake | *Megamonas unclassified* | -0.04 | -0.21 | 0.14 | 0.09 | -0.43 | 0.671 | 0.883 |
| Fruit intake | *Megamonas unclassified* | 0.04 | -0.14 | 0.22 | 0.09 | 0.46 | 0.644 | 0.883 |
| Vegetable intake | *Megasphaera unclassified* | 0.05 | -0.14 | 0.24 | 0.09 | 0.54 | 0.593 | 0.883 |
| Red and processed meat intake | *Megasphaera unclassified* | -0.04 | -0.23 | 0.16 | 0.1 | -0.38 | 0.706 | 0.905 |
| Fish intake | *Parabacteroides unclassified* | -0.03 | -0.21 | 0.15 | 0.09 | -0.31 | 0.757 | 0.941 |
| Dairy intake | *Parabacteroides unclassified* | 0.02 | -0.16 | 0.2 | 0.09 | 0.2 | 0.844 | 0.941 |
| Vegetable intake | *Lachnospiraceae bacterium 3 1 57FAA CT1* | -0.03 | -0.22 | 0.16 | 0.1 | -0.26 | 0.792 | 0.941 |
| Dairy intake | *Lachnospiraceae bacterium 7 1 58FAA* | -0.02 | -0.21 | 0.17 | 0.1 | -0.19 | 0.847 | 0.941 |
| Vegetable intake | *Megamonas hypermegale* | 0.02 | -0.16 | 0.2 | 0.09 | 0.23 | 0.815 | 0.941 |
| Red and processed meat intake | *Megamonas unclassified* | -0.02 | -0.2 | 0.16 | 0.09 | -0.2 | 0.841 | 0.941 |
| Fruit intake | *Clostridium hathewayi* | -0.01 | -0.21 | 0.19 | 0.1 | -0.09 | 0.931 | 0.97 |
| Dairy intake | *Clostridium hathewayi* | -0.01 | -0.2 | 0.18 | 0.1 | -0.1 | 0.922 | 0.97 |
| Vegetable intake | *Adlercreutzia equolifaciens* | -0.01 | -0.19 | 0.17 | 0.09 | -0.12 | 0.905 | 0.97 |
| Fruit intake | *Parabacteroides unclassified* | 0 | -0.19 | 0.18 | 0.09 | -0.03 | 0.979 | 0.979 |
| Vegetable intake | *Lachnospiraceae bacterium 7 1 58FAA* | 0 | -0.19 | 0.19 | 0.1 | -0.03 | 0.974 | 0.979 |

*The prospective associations between dietary factors (higher group *vs*. lower group) and identified microbes were assessed using the multivariable linear models, adjusted for age, sex, BMI, smoking status, alcohol status, education, income, physical activity, total energy intake, Bristol stool score, time interval, and corresponding baseline microbe abundance. All phenotypes were standardized into Z-scores. FDR, false discovery rate; CI, confidence interval.

# Table S9. Replication of the associations between baseline BMI and follow-up microbes in the HMP cohort*

| Microbes | β_GNHS_ | β_HMP_ | *P*_HMP_ | β_meta_ | *P*_meta_ | *P*_heterogeneity_ | *I^2^* | Prevalence in GNHS | Prevalence in HMP |
| --- | --- | --- | --- | --- | --- | --- | --- | --- | --- |
| *Adlercreutzia equolifaciens* | 0.14(0.05, 0.23) | 0.13(-0.21, 0.47) | 0.456 | 0.14(0.06, 0.22) | <0.001 | 0.96 | 0 | 0.53 | 0.69 |
| *Parabacteroides unclassified* | -0.12(-0.2, -0.03) | -0.03(-0.29, 0.22) | 0.808 | -0.11(-0.19, -0.04) | 0.003 | 0.519 | 0 | 0.48 | 0.34 |
| *Lachnospiraceae bacterium 3 1 57FAA CT1* | -0.12(-0.21, -0.03) | -0.04(-0.38, 0.31) | 0.843 | -0.11(-0.21, -0.02) | 0.018 | 0.648 | 0 | 0.35 | 0.31 |
| *Lachnospiraceae bacterium 7 1 58FAA* | -0.12(-0.21, -0.03) | -0.3(-0.59, -0.01) | 0.052 | -0.15(-0.29, -0.02) | 0.029 | 0.254 | 23.24 | 0.83 | 0.99 |
| *Clostridium hathewayi* | -0.16(-0.26, -0.07) | 0.01(-0.34, 0.35) | 0.974 | -0.15(-0.24, -0.05) | 0.002 | 0.364 | 0 | 0.81 | 0.4 |
| *Ruminococcus sp 5 1 39BFAA* | 0.12(0.03, 0.21) | -0.1(-0.46, 0.27) | 0.607 | 0.09(-0.07, 0.24) | 0.276 | 0.261 | 20.78 | 0.28 | 0.58 |
| *Megamonas hypermegale* | 0.11(0.02, 0.19) | 0(-0.03, 0.02) | 0.752 | 0.05(-0.06, 0.16) | 0.409 | 0.006 | 86.52 | 0.36 | 0.05 |
| *Megamonas unclassified* | 0.11(0.03, 0.2) | -0.06(-0.24, 0.12) | 0.542 | 0.05(-0.11, 0.21) | 0.558 | 0.096 | 63.89 | 0.52 | 0.06 |
| *Megasphaera unclassified* | 0.14(0.05, 0.22) | -0.12(-0.42, 0.18) | 0.443 | 0.05(-0.19, 0.29) | 0.666 | 0.104 | 62.13 | 0.38 | 0.09 |
| *Bacteroides caccae* | 0.11(0.03, 0.19) | -0.16(-0.32, 0) | 0.062 | -0.01(-0.28, 0.25) | 0.913 | 0.003 | 88.36 | 0.68 | 0.72 |

*Multivariable linear regression models were used to estimate the difference in the abundance of gut microbes (in SD unit of the log-transformed abundance) per 1-SD difference in BMI, adjusted for age, sex, race (white/not white), time interval, and corresponding baseline microbe abundance. The meta-analysis with a random effects model was used to integrate the results from GNHS and HMP cohorts, and the heterogeneity was assessed using *I^2^* and Cochran-Q test.

# Table S10. The association between gut microbes and insulin resistance related phenotypes*

| **Taxonomy** | **Phenotype** | **Estimate** | **CI**  **lower** | **CI**  **upper** | **SE** | ***t* value** | ***p*** | **FDR** |
| --- | --- | --- | --- | --- | --- | --- | --- | --- |
| *Clostridium hathewayi* | HOMA-IR | -0.1 | -0.18 | -0.02 | 0.04 | -2.51 | 0.012 | ***0.03*** |
| *Lachnospiraceae bacterium 3 1 57FAA CT1* | HOMA-IR | -0.13 | -0.22 | -0.05 | 0.04 | -2.99 | 0.003 | ***0.014*** |
| *Megamonas hypermegale* | HOMA-IR | 0.02 | -0.06 | 0.1 | 0.04 | 0.5 | 0.618 | 0.773 |
| *Megamonas unclassified* | HOMA-IR | -0.01 | -0.09 | 0.07 | 0.04 | -0.17 | 0.861 | 0.861 |
| *Bacteroides caccae* | HOMA-IR | -0.04 | -0.12 | 0.05 | 0.04 | -0.85 | 0.394 | 0.657 |
| *Clostridium hathewayi* | HbA1c | -0.01 | -0.07 | 0.05 | 0.03 | -0.25 | 0.801 | 0.929 |
| *Lachnospiraceae bacterium 3 1 57FAA CT1* | HbA1c | -0.02 | -0.08 | 0.04 | 0.03 | -0.57 | 0.568 | 0.929 |
| *Megamonas hypermegale* | HbA1c | 0.07 | 0.01 | 0.13 | 0.03 | 2.15 | 0.032 | 0.12 |
| *Megamonas unclassified* | HbA1c | 0.09 | 0.03 | 0.15 | 0.03 | 2.81 | 0.005 | ***0.037*** |
| *Bacteroides caccae* | HbA1c | -0.03 | -0.1 | 0.03 | 0.03 | -1.04 | 0.297 | 0.892 |
| *Clostridium hathewayi* | Fasting insulin | -0.11 | -0.19 | -0.03 | 0.04 | -2.57 | 0.01 | 0.051 |
| *Lachnospiraceae bacterium 3 1 57FAA CT1* | Fasting insulin | -0.13 | -0.22 | -0.04 | 0.04 | -2.9 | 0.004 | ***0.037*** |
| *Megamonas hypermegale* | Fasting insulin | 0 | -0.08 | 0.08 | 0.04 | 0.09 | 0.929 | 0.929 |
| *Megamonas unclassified* | Fasting insulin | -0.03 | -0.11 | 0.06 | 0.04 | -0.63 | 0.53 | 0.929 |
| *Bacteroides caccae* | Fasting insulin | -0.04 | -0.12 | 0.05 | 0.04 | -0.84 | 0.402 | 0.929 |
| *Clostridium hathewayi* | Fasting glucose | 0 | -0.05 | 0.06 | 0.03 | 0.14 | 0.89 | 0.929 |
| *Lachnospiraceae bacterium 3 1 57FAA CT1* | Fasting glucose | -0.01 | -0.06 | 0.05 | 0.03 | -0.2 | 0.84 | 0.929 |
| *Megamonas hypermegale* | Fasting glucose | 0.01 | -0.05 | 0.07 | 0.03 | 0.35 | 0.727 | 0.929 |
| *Megamonas unclassified* | Fasting glucose | 0.02 | -0.05 | 0.08 | 0.03 | 0.5 | 0.614 | 0.929 |
| *Bacteroides caccae* | Fasting glucose | 0.01 | -0.06 | 0.07 | 0.03 | 0.18 | 0.855 | 0.929 |

*Linear mixed-effect models were used to estimate the difference in insulin resistance related phenotypes (in SD unit) per 1-SD difference in the log-transformed abundance of gut microbes, adjusted for age, sex, smoking status, alcohol status, education, income, physical activity, and total energy intake. Fasting insulin, HOMA-IR, and fasting glucose were log-transformed. HOMA-IR, homeostasis model assessment of insulin resistance; HbA1c, hemoglobin A1c; FDR, false discovery rate; CI, confidence interval.

# Table S11. The prospective associations between dietary factors and insulin resistance related phenotypes*

| Diet | Phenotype | Estimate | CI lower | CI upper | SE | *t* value | *p* | FDR |
| --- | --- | --- | --- | --- | --- | --- | --- | --- |
| Dairy intake | HOMA-IR | 0.29 | 0.01 | 0.57 | 0.14 | 2.03 | 0.044 | 0.44 |
| Dairy intake | Fasting insulin | 0.3 | 0.01 | 0.59 | 0.15 | 2.07 | 0.041 | 0.44 |
| Vegetable intake | HOMA-IR | 0.15 | -0.14 | 0.43 | 0.14 | 1.03 | 0.303 | 0.618 |
| Fruit intake | HOMA-IR | 0.21 | -0.07 | 0.49 | 0.14 | 1.48 | 0.142 | 0.618 |
| Fish intake | HOMA-IR | 0.16 | -0.14 | 0.46 | 0.15 | 1.04 | 0.302 | 0.618 |
| Vegetable intake | Fasting insulin | 0.15 | -0.14 | 0.44 | 0.15 | 1.02 | 0.309 | 0.618 |
| Fruit intake | Fasting insulin | 0.19 | -0.1 | 0.47 | 0.15 | 1.29 | 0.2 | 0.618 |
| Fish intake | Fasting insulin | 0.2 | -0.11 | 0.51 | 0.16 | 1.27 | 0.207 | 0.618 |
| Dairy intake | HbA1c | -0.08 | -0.22 | 0.06 | 0.07 | -1.09 | 0.275 | 0.618 |
| Fish intake | Fasting glucose | -0.09 | -0.24 | 0.06 | 0.08 | -1.18 | 0.239 | 0.618 |
| Red and processed meat intake | Fasting glucose | 0.07 | -0.09 | 0.23 | 0.08 | 0.88 | 0.379 | 0.689 |
| Red and processed meat intake | HOMA-IR | -0.04 | -0.34 | 0.26 | 0.15 | -0.25 | 0.8 | 0.894 |
| Red and processed meat intake | Fasting insulin | -0.04 | -0.35 | 0.27 | 0.16 | -0.24 | 0.81 | 0.894 |
| Vegetable intake | HbA1c | 0.01 | -0.14 | 0.16 | 0.07 | 0.16 | 0.874 | 0.894 |
| Fruit intake | HbA1c | 0.01 | -0.14 | 0.16 | 0.07 | 0.13 | 0.894 | 0.894 |
| Fish intake | HbA1c | -0.02 | -0.17 | 0.13 | 0.08 | -0.3 | 0.764 | 0.894 |
| Red and processed meat intake | HbA1c | -0.04 | -0.2 | 0.11 | 0.08 | -0.54 | 0.589 | 0.894 |
| Vegetable intake | Fasting glucose | 0.02 | -0.13 | 0.17 | 0.08 | 0.23 | 0.815 | 0.894 |
| Fruit intake | Fasting glucose | 0.03 | -0.13 | 0.18 | 0.08 | 0.35 | 0.727 | 0.894 |
| Dairy intake | Fasting glucose | 0.04 | -0.11 | 0.19 | 0.08 | 0.54 | 0.587 | 0.894 |

* The prospective associations between dietary factors (higher group *vs*. lower group) and insulin resistance related phenotypes were assessed using the multivariable linear models, adjusted for age, sex, BMI, smoking status, alcohol status, education, income, physical activity, total energy intake, time interval, and corresponding baseline levels of insulin resistance related phenotypes. Fasting insulin, HOMA-IR, and fasting glucose were log-transformed. All phenotypes were standardized into Z-scores. HOMA-IR, homeostasis model assessment of insulin resistance; HbA1c, hemoglobin A1c; FDR, false discovery rate; CI, confidence interval.

# Table S12. The prospective association between weight group and insulin resistance related phenotypes*

|  | Underweight | | | | | Adiposity | | | | |
| --- | --- | --- | --- | --- | --- | --- | --- | --- | --- | --- |
| Phenotype | Estimate | CI lower | CI upper | *p* | FDR | Estimate | CI lower | CI upper | *p* | FDR |
| HOMA-IR | -0.62 | -1.34 | 0.11 | 0.096 | 0.192 | 0.59 | 0.26 | 0.92 | 0.001 | 0.001 |
| Fasting insulin | -0.74 | -1.48 | 0.01 | 0.052 | 0.192 | 0.61 | 0.27 | 0.94 | <0.001 | 0.001 |
| HbA1c | -0.03 | -0.48 | 0.43 | 0.913 | 0.913 | 0.16 | 0.01 | 0.31 | 0.036 | 0.048 |
| Fasting glucose | -0.21 | -0.64 | 0.22 | 0.338 | 0.451 | 0.15 | 0 | 0.31 | 0.053 | 0.053 |

* The prospective associations between weight group (underweight, normal weight, and adiposity) and insulin resistance were assessed using multivariable linear regression models, adjusted for age, sex, smoking status, alcohol status, education, income, physical activity, total energy intake, time interval, and corresponding baseline levels of insulin resistance related phenotypes. The normal weight was served as the reference group. Adiposity was defined as overweight or obesity. Fasting insulin, HOMA-IR, and fasting glucose were log-transformed. All phenotypes were standardized into Z-scores. HOMA-IR, homeostasis model assessment of insulin resistance; HbA1c, hemoglobin A1c; FDR, false discovery rate; CI, confidence interval.

# Table S13. The association between *Lachnospiraceae bacterium 3 1 57FAA CT1* and PWY-5022 pathway*

|  | **Estimate** | **SE** | ***t*** | ***p*** |
| --- | --- | --- | --- | --- |
| PWY-5022 | 0.12 | 0.03 | 3.48 | <0.001 |

*Linear mixed-effect model was used to estimate the difference in the abundance of PWY-5022 pathway (in SD unit of the log-transformed abundance) per 1-SD difference in the log-transformed abundance of *Lachnospiraceae bacterium 3 1 57FAA CT1*, adjusted for age, sex, smoking status, alcohol status, education, income, physical activity, total energy intake, and Bristol stool score. SE, standard error.

# Table S14. The associations between *Lachnospiraceae bacterium 3 1 57FAA CT1* and other pathways apart from PWY-5022*

| Pathway Name | Pathway Description | Estimate | SE | *t* | *p* | FDR |
| --- | --- | --- | --- | --- | --- | --- |
| HISDEG-PWY | L-histidine degradation I | 0.2 | 0.03 | 6.03 | 1.69E-09 | 3.61E-07 |
| PWY-6700 | queuosine biosynthesis | -0.2 | 0.03 | -5.88 | 4.19E-09 | 4.48E-07 |
| PWY-4981 | L-proline biosynthesis II (from arginine) | 0.19 | 0.03 | 5.64 | 1.68E-08 | 1.20E-06 |
| UNINTEGRATED | UNINTEGRATED | -0.16 | 0.03 | -4.95 | 7.57E-07 | 4.05E-05 |
| PWY-7383 | anaerobic energy metabolism (invertebrates, cytosol) | 0.16 | 0.03 | 4.78 | 1.79E-06 | 7.68E-05 |
| PWY-6151 | S-adenosyl-L-methionine cycle I | -0.15 | 0.03 | -4.3 | 1.67E-05 | 0.000596523 |
| UNMAPPED | UNMAPPED | 0.14 | 0.03 | 4.25 | 2.17E-05 | 0.00066192 |
| TRNA-CHARGING-PWY | tRNA charging | 0.14 | 0.03 | 4.18 | 2.91E-05 | 0.000777293 |
| PWY-5667 | CDP-diacylglycerol biosynthesis I | -0.14 | 0.03 | -4.06 | 4.96E-05 | 0.001061316 |
| PWY0-1319 | CDP-diacylglycerol biosynthesis II | -0.14 | 0.03 | -4.06 | 4.92E-05 | 0.001061316 |
| PWY66-399 | gluconeogenesis III | 0.13 | 0.03 | 3.97 | 7.16E-05 | 0.001393914 |
| PWY-5101 | L-isoleucine biosynthesis II | 0.13 | 0.03 | 3.88 | 0.00010333 | 0.001842713 |
| ENTBACSYN-PWY | enterobactin biosynthesis | 0.13 | 0.03 | 3.67 | 0.000241795 | 0.003980323 |
| PWY-6703 | preQ0 biosynthesis | -0.12 | 0.03 | -3.54 | 0.000399591 | 0.005700825 |
| PWY-7234 | inosine-5'-phosphate biosynthesis III | 0.12 | 0.03 | 3.55 | 0.000390568 | 0.005700825 |
| RHAMCAT-PWY | L-rhamnose degradation I | -0.12 | 0.03 | -3.46 | 0.000532587 | 0.007123352 |
| DAPLYSINESYN-PWY | L-lysine biosynthesis I | 0.11 | 0.03 | 3.44 | 0.000583833 | 0.007349425 |
| PWY-5030 | L-histidine degradation III | 0.12 | 0.03 | 3.36 | 0.000776009 | 0.00922589 |
| PWY-5695 | urate biosynthesis/inosine 5'-phosphate degradation | -0.11 | 0.03 | -3.29 | 0.00099905 | 0.011252455 |
| PWY-2942 | L-lysine biosynthesis III | -0.11 | 0.03 | -3.25 | 0.001151381 | 0.012319776 |
| PANTO-PWY | phosphopantothenate biosynthesis I | -0.11 | 0.03 | -3.17 | 0.001534568 | 0.014927158 |
| PWY-5723 | Rubisco shunt | 0.11 | 0.03 | 3.17 | 0.001527329 | 0.014927158 |
| PWY-5154 | L-arginine biosynthesis III (via N-acetyl-L-citrulline) | 0.11 | 0.03 | 3.14 | 0.001717469 | 0.015345403 |
| PWY-5838 | superpathway of menaquinol-8 biosynthesis I | 0.11 | 0.03 | 3.12 | 0.001792687 | 0.015345403 |
| PWY-7204 | pyridoxal 5'-phosphate salvage II (plants) | 0.11 | 0.03 | 3.13 | 0.001757465 | 0.015345403 |
| PWY-5861 | superpathway of demethylmenaquinol-8 biosynthesis | 0.11 | 0.03 | 3.08 | 0.002095221 | 0.016606564 |
| PWY-5973 | cis-vaccenate biosynthesis | -0.11 | 0.03 | -3.08 | 0.002038271 | 0.016606564 |
| PWY-7663 | gondoate biosynthesis (anaerobic) | -0.1 | 0.03 | -2.99 | 0.002749005 | 0.021010253 |
| GLYCOLYSIS-TCA-GLYOX-BYPASS | superpathway of glycolysis, pyruvate dehydrogenase, TCA, and glyoxylate bypass | 0.1 | 0.03 | 2.86 | 0.004174208 | 0.027637779 |
| HSERMETANA-PWY | L-methionine biosynthesis III | 0.1 | 0.03 | 2.85 | 0.004393603 | 0.027637779 |
| P42-PWY | incomplete reductive TCA cycle | 0.1 | 0.03 | 2.86 | 0.004282562 | 0.027637779 |
| PWY-3841 | folate transformations II | -0.1 | 0.03 | -2.84 | 0.004520198 | 0.027637779 |
| PWY-7228 | superpathway of guanosine nucleotides de novo biosynthesis I | -0.1 | 0.03 | -2.87 | 0.004083469 | 0.027637779 |
| RIBOSYN2-PWY | flavin biosynthesis I (bacteria and plants) | 0.1 | 0.03 | 2.87 | 0.004066685 | 0.027637779 |
| TCA-GLYOX-BYPASS | superpathway of glyoxylate bypass and TCA | 0.1 | 0.03 | 2.89 | 0.003795495 | 0.027637779 |
| PWY-6609 | adenine and adenosine salvage III | -0.1 | 0.03 | -2.81 | 0.004981325 | 0.029611212 |
| PWY-6630 | superpathway of L-tyrosine biosynthesis | 0.09 | 0.03 | 2.74 | 0.006070868 | 0.035112591 |
| PWY-621 | sucrose degradation III (sucrose invertase) | -0.09 | 0.03 | -2.72 | 0.006577224 | 0.037040154 |
| PWY66-400 | glycolysis VI (metazoan) | -0.09 | 0.03 | -2.69 | 0.007144473 | 0.039203006 |
| PWY-5097 | L-lysine biosynthesis VI | -0.09 | 0.03 | -2.61 | 0.009097704 | 0.047338575 |
| PWY-7211 | superpathway of pyrimidine deoxyribonucleotides de novo biosynthesis | -0.09 | 0.03 | -2.6 | 0.009290748 | 0.047338575 |
| PWY-7357 | thiamin formation from pyrithiamine and oxythiamine (yeast) | -0.09 | 0.03 | -2.61 | 0.008980532 | 0.047338575 |
| PWY-6629 | superpathway of L-tryptophan biosynthesis | 0.09 | 0.03 | 2.59 | 0.009681504 | 0.048182368 |

*Linear mixed-effect model was used to estimate the difference in the abundance of other pathways apart from PWY-5022 (in SD unit of the log-transformed abundance) per 1-SD difference in the log-transformed abundance of *Lachnospiraceae bacterium 3 1 57FAA CT1*, adjusted for age, sex, smoking status, alcohol status, education, income, physical activity, total energy intake, and Bristol stool score. SE, standard error.

# Supplementary figures

# Fig. S1. The associations between BMI/long-term weight change and gut microbiota. (A) The temporal relationship between adiposity and α- and β-diversity. The cross-lagged path analysis was used to estimate the difference in α- and β-diversity (in SD unit) per 1-SD difference in BMI, and the difference in BMI (in SD unit) per 1-SD difference in α- and β-diversity, adjusted for age, sex, smoking status, alcohol status, education, income, physical activity, total energy intake, Bristol stool score and time interval. (B) The associations between baseline gut microbes and follow-up adiposity. The cross-lagged path analysis was used to estimate the difference in BMI (in SD unit) per 1-SD difference in the log-transformed abundance of gut microbes, adjusted for age, sex, smoking status, alcohol status, education, income, physical activity, total energy intake, Bristol stool score and time interval. (C) Prospective associations between long-term weight change and α- and β-diversity. Multivariable linear regression models were used to estimate the difference in α- and β-diversity (in SD unit) comparing different weight change patterns (the stable normal group was served as the reference group), adjusted for age, sex, smoking status, alcohol status, education, income, physical activity, total energy intake, Bristol stool score, time interval and corresponding baseline α- and β-diversity. (D) Gut microbes that were affected by the adiposity to normal group compared with the stable normal group. Multivariable linear regression models were used to estimate the difference in the abundance of gut microbes (in SD unit of the log-transformed abundance) comparing the adiposity to normal group with the stable normal group, adjusted for age, sex, smoking status, alcohol status, education, income, physical activity, total energy intake, Bristol stool score, time interval and corresponding baseline microbe abundance. CI, confidence interval; FDR, false discovery rate; PCo, Principal coordinates.

**
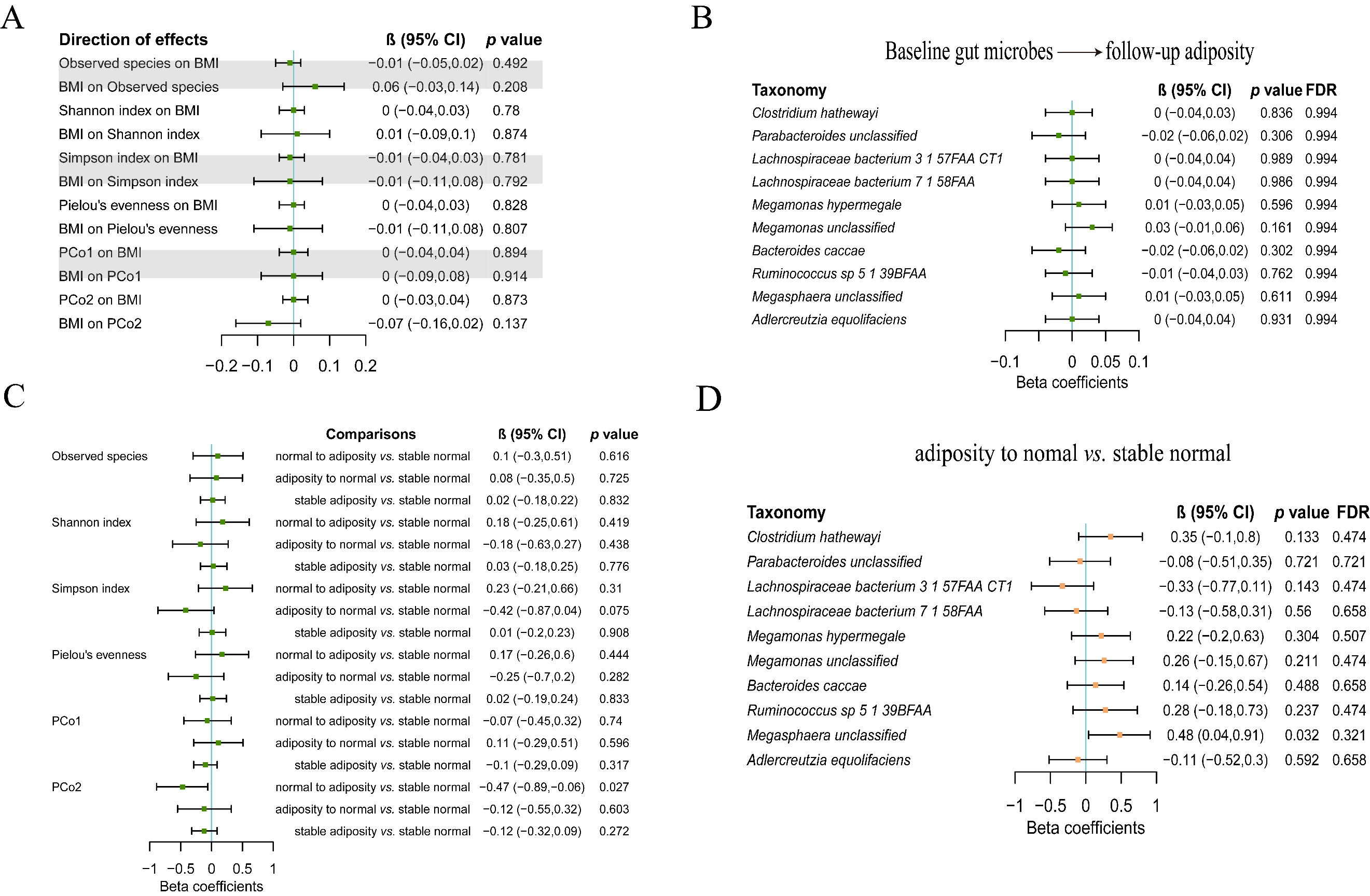
**

# Fig. S2. The associations between BMI/long-term weight change and α-diversity further adjusting for sequencing depths. (A) The temporal relationship between adiposity and α-diversity further adjusting for sequencing depths. The cross-lagged path analysis was used to estimate the difference in α-diversity (in SD unit) per 1-SD difference in BMI, and the difference in BMI (in SD unit) per 1-SD difference in α-diversity, adjusted for age, sex, smoking status, alcohol status, education, income, physical activity, total energy intake, Bristol stool score, time interval and sequencing depths. (B) Prospective associations between long-term weight change and α-diversity further adjusting for sequencing depths. Multivariable linear regression models were used to estimate the difference in α-diversity (in SD unit) comparing different weight change patterns (the stable normal group was served as the reference group), adjusted for age, sex, smoking status, alcohol status, education, income, physical activity, total energy intake, Bristol stool score, time interval, sequencing depths and corresponding baseline α-diversity.

**
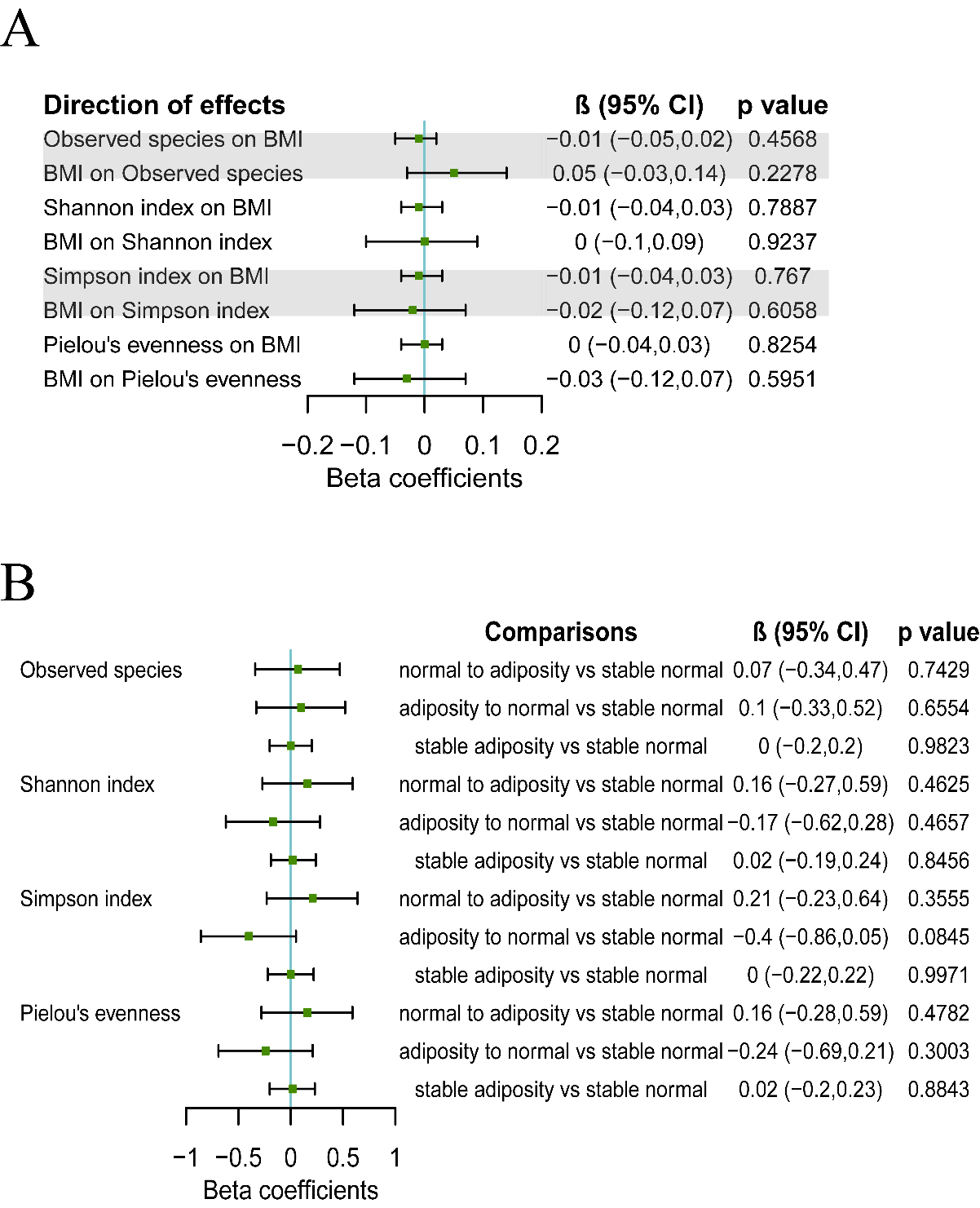
**

# Fig. S3. The correlations among identified BMI-associated microbes. The Spearman correlation analysis was used to examine the correlations among identified BMI-associated microbes

**
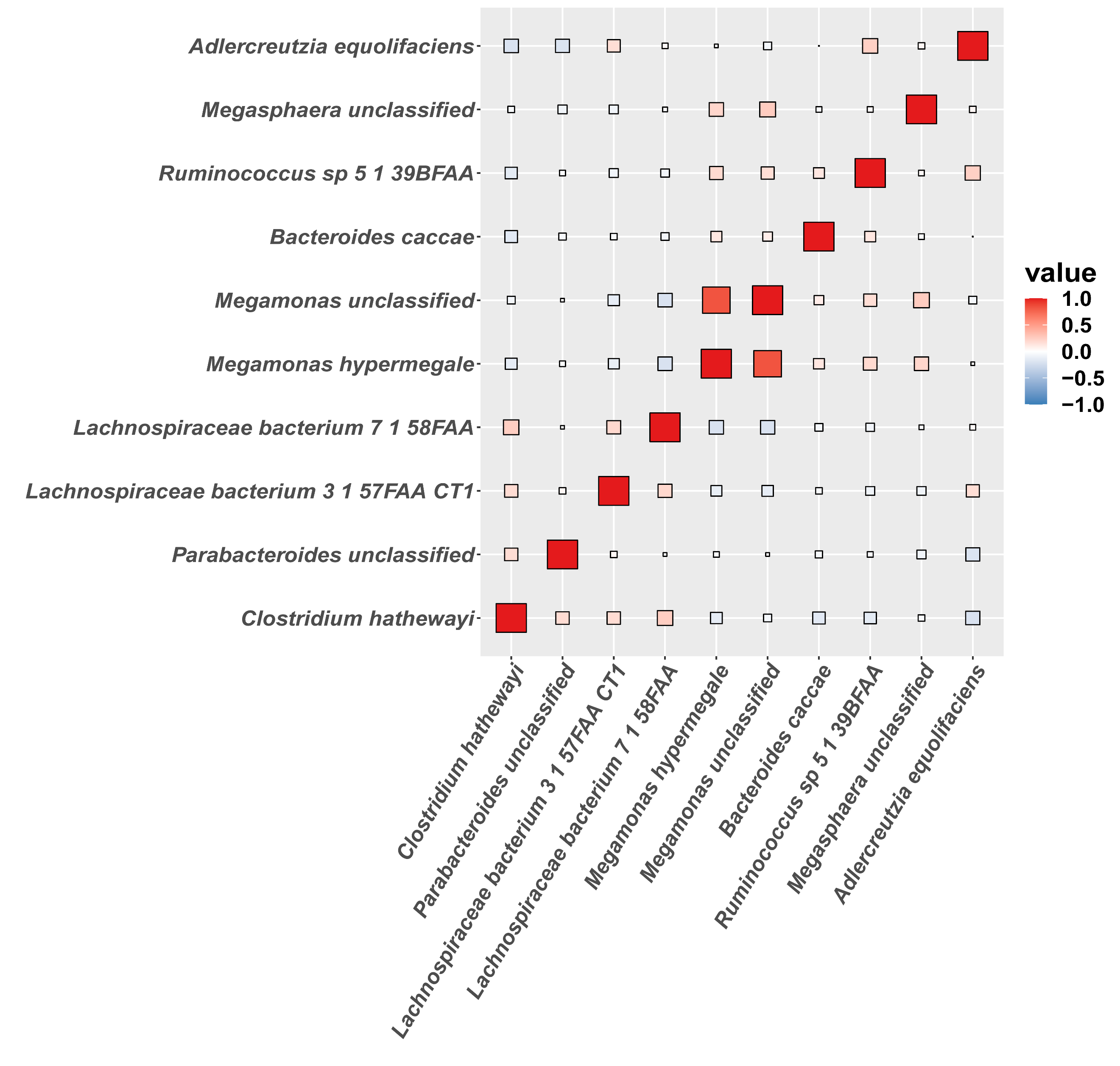
**

# Fig. S4. The correlation between the regression coefficients for females and those for males. Pearson correlation analysis was used to calculate the correlation between the regression coefficients for females and those for males

**
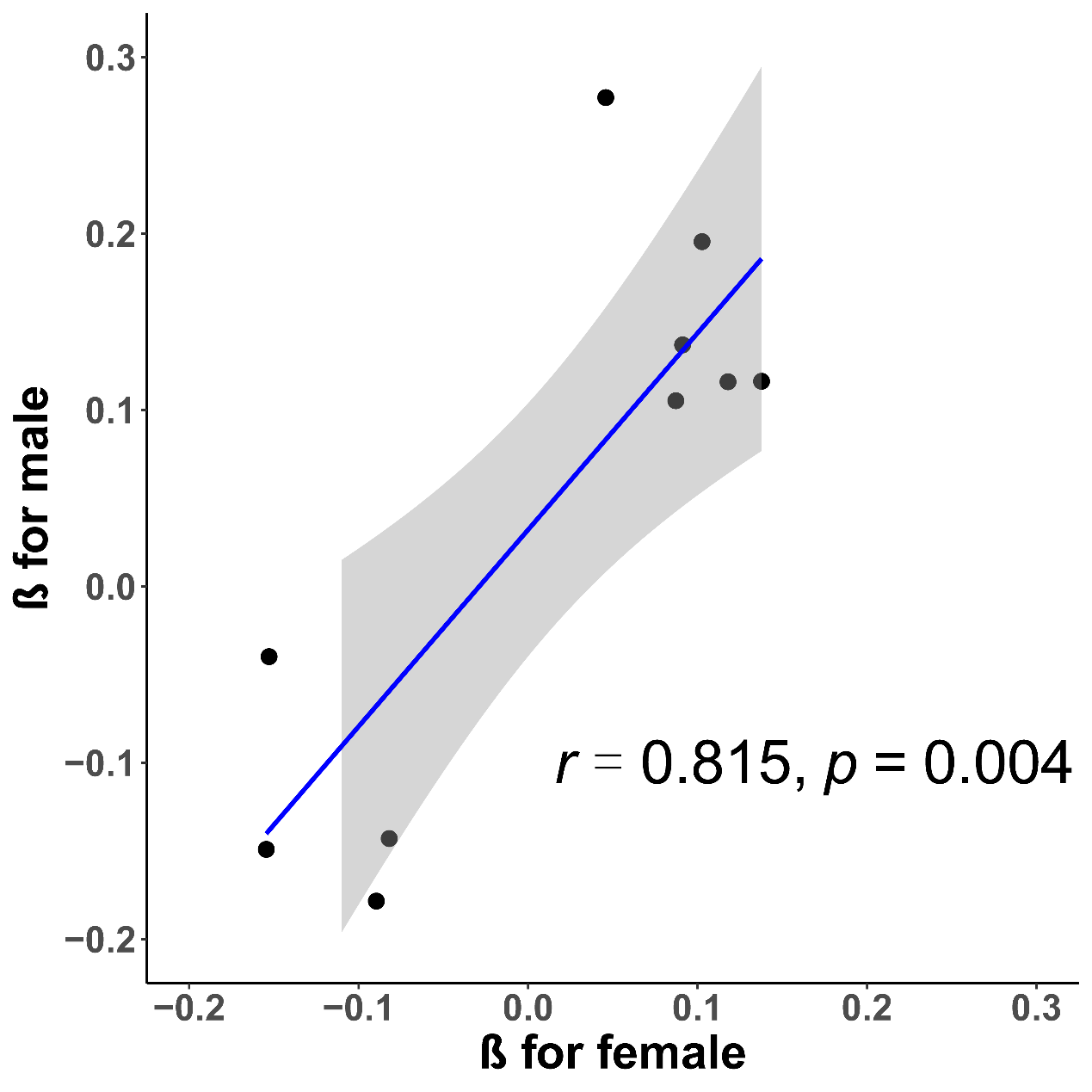
**

# Fig. S5. Global comparison of 1-SD difference of BMI versus WC associated with difference (in SD unit) of (A) all gut microbial features including gut microbes and α- and β-diversity, and (B) ten identified gut microbes. The effect of BMI or WC on each gut microbial feature was obtained by cross-lagged path analysis, adjusted for age, sex, smoking status, alcohol status, education, income, physical activity, total energy intake, Bristol stool score and time interval. The correlation between the regression coefficients for the association of BMI with microbial features and those for the association of WC with microbial features was calculated by Pearson correlation. WC, waist circumference; SD, standard deviation; CI, confidence interval.


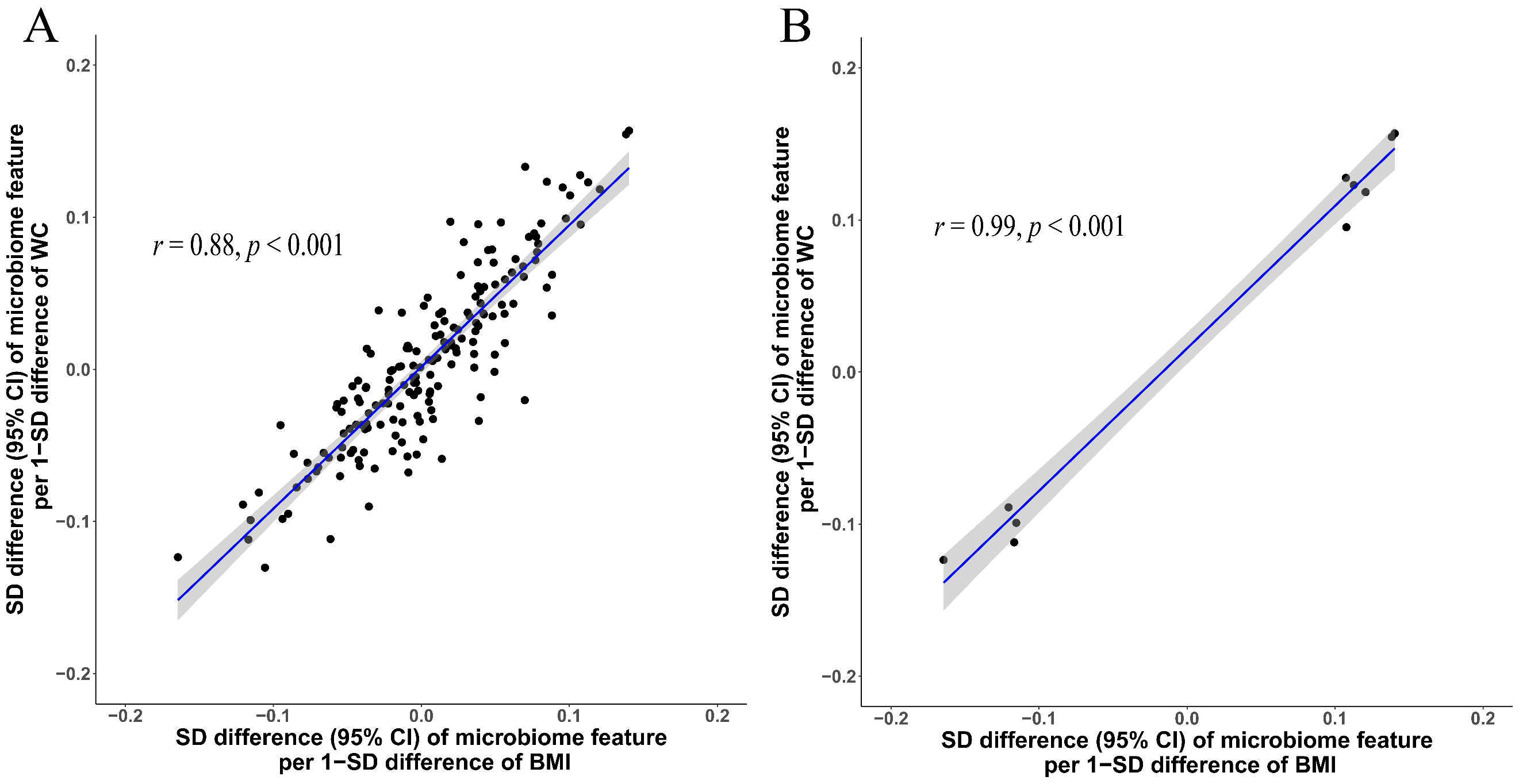

Supplement: Supplementary file 1 — Additional file 1: Table S1. [Characteristics of the study participants between baseline and follow-up]. Table S2. [The temporal relationship between adiposity and α-diversity and β-diversity]. Table S3. [The temporal relationship between adiposity and gut microbes]. Table S4. [The correlation of identified microbes between two time points]. Table S5. [The differences in the associations of BMI with identified gut microbes between females and males]. Table S6. [The temporal relationship between WC and α-diversity and β-diversity]. Table S7. [The temporal relationship between WC and gut microbes]. Table S8. [The prospective associations between dietary factors and identified microbes]. Table S9. [Replication of the associations between baseline BMI and follow-up microbes in the HMP cohort]. Table S10. [The association between gut microbes and insulin resistance related phenotypes]. Table S11. [The prospective associations between dietary factors and insulin resistance related phenotypes]. Table S12. [The prospective association between weight group and insulin resistance related phenotypes]. Table S13. [The association between Lachnospiraceae bacterium 3 1 57FAA CT1 and PWY-5022 pathway]. Table S14. [The associations between Lachnospiraceae bacterium 3 1 57FAA CT1 and other pathways apart from PWY-5022]. Figure S1. [The associations between BMI/long-term weight change and gut microbiota]. Figure S2. [The associations between BMI/long-term weight change and α-diversity further adjusting for sequencing depths]. Figure S3. [The correlations among identified BMI-associated microbes]. Figure S4. [The correlation between the regression coefficients for females and those for males]. Figure S5. [Global comparison of 1-SD difference of BMI versus WC associated with difference (in SD unit) of all gut microbial features and ten identified gut microbes]. [file 12916_2022_2376_MOESM1_ESM.docx]
